# Final E_5_ to E_8_ Steps in the Nitrogenase Mechanism for Nitrogen Fixation

**DOI:** 10.1021/acs.jpcb.4c04331

**Published:** 2024-09-30

**Authors:** Per E. M. Siegbahn

**Affiliations:** Department of Organic Chemistry, Arrhenius Laboratory, Stockholm University, SE-106 91 Stockholm, Sweden

## Abstract

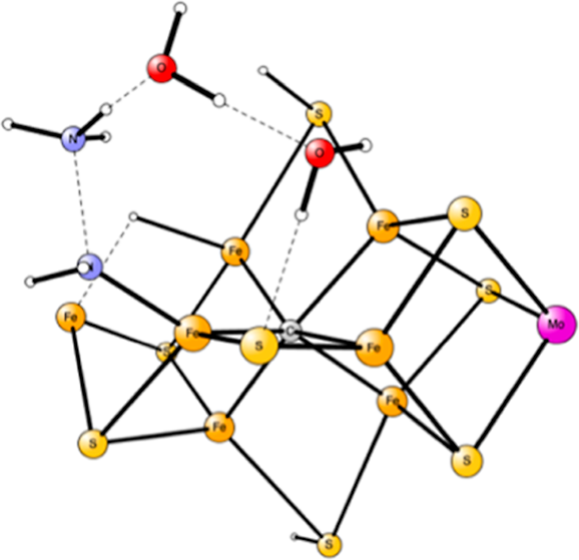

Nitrogenase converts nitrogen in the air to ammonia. It is often regarded as the second most important enzyme in nature after photosystem II. The mechanism for how nitrogenase is able to perform the difficult task of cleaving the strong bond in N_2_ is debated. It is known that for every electron that is donated to N_2_, two ATP are hydrolyzed. In the experimentally suggested mechanism, the activation occurs after four reductions of the ground state, but there is no suggestion for how the enzyme uses the hydrolysis energy to perform catalysis. In the theoretical mechanism, it is suggested that hydrolysis is used to reduce the electron donor. In previous papers, the steps leading to the activation of N_2_ in the so-called E_4_ state has been investigated, using both the experimental and theoretical mechanism, showing that only the theoretical one leads to agreement with EPR observations for E_4_. In the present paper, the four steps following E_4_, leading to the release of two ammonia molecules, are described using the same methodology as used in the previous studies.

## 1 Introduction

**Figure 1:**
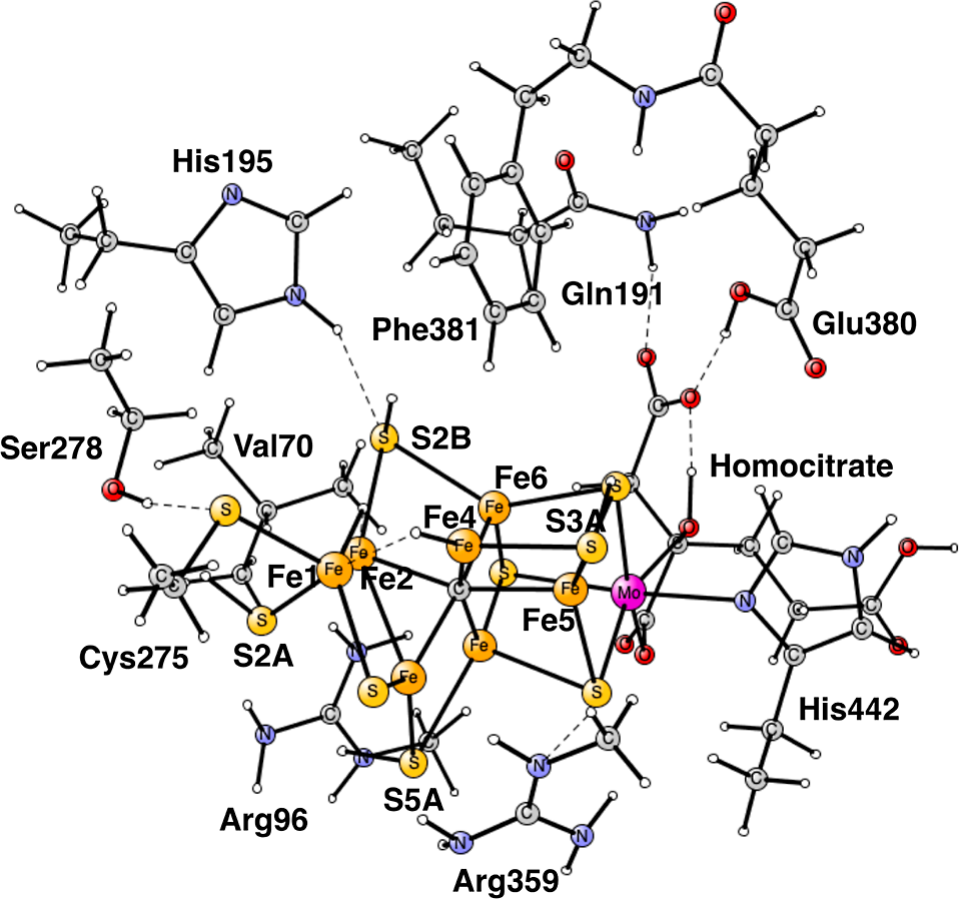
Model used for the present study, showing which amino acids were included. The model is built on the X-ray structure 3U7Q,^2^ and is here shown at the start of E_4_ with two hydrides. The numbering follows the one of the X-ray structure.

Nitrogenases are the only enzymes able to convert nitrogen in the air to biologically useful products. An X-ray structure for the active cofactor was determined already in 1992.^1^ It contains one molybdenum and seven irons connected by sulfide bridges, see Figure 1. In 2011 it was found that there is a carbide in the center of the cofactor.^2^ No X-ray structures in between the ground state and the final state have been determined, and a mechanism for the entire process has therefore been very difficult to determine. It has long been known that N_2_ becomes bound to the cofactor after four reductions of the cofactor.^3^ Kinetic experiments led to the so-called Lowe-Thorneley (LT) scheme. A major breakthrough occurred when details of the E_4_ mechanism was found by the use of EPR.^4−7^ It was found that two hydrides were released as H_2_ concertedly with N_2_ activation. It was suggested that the E_4_ state is the same state as observed after four reductions of the ground state in the LT scheme. However, this conclusion has been criticized by model calculations, which found that the two states are different.^8−10^ While the E_4_ state has two hydrides, calculations suggested that the state obtained after four reductions of the ground state has only one hydride.^11^

A conclusion drawn from the model calculations is that a preactivation with four reductions, A_1_ to A_4_, is required before catalysis starts with the E_0_ state. Furthermore, during the preactivation, a sulfide is lost from the cofactor. A mechanism was suggested for N_2_ activation in the E_4_ state leading to the first protonations of N_2_.^12^ The calculated mechanism is in perfect agreement with the EPR observations.^4−7^ In contrast, for the experimentally suggested structures and mechanism, big errors of 40 kcal/mol in the computational results are required for agreement with the EPR.^11^ Errors of more than 10 kcal/mol have never been seen, using the present methods.^12−15^ The present study is a continuation of the previous one of the E_4_ state. The progress of the N_2_ protonations is followed all the way to the final E_8_ state, including the release of the two ammonia products.

Even though many computational studies of nitrogenase have been published there is not anyone with a similarity to the one in the present paper. There is only one study of the E_5_ to E_8_ states.^16^ However, since no transition energies between the E-states were computed and no water molecules were added for the transition states, any comparison to the results of the present study is not possible.

## 2 Methods

The methods used here are the same as the ones used in the previous study of the E_4_ state,^12^ and in many other studies of redox enzymes.^13^ The accuracy is generally about 3 kcal/mol.^14,15^ A modified form of B3LYP^17^ is used with 15% exact exchange instead of 20%, based on experience from redox reactions involving the first transition row metals.^13−15^ The geometry optimization was done using the unmodified form of B3LYP with a lacvp* basis set. For the final geometries, a large basis set was used with lacv3p+ on the metals and cc-pvtz(-f) basis for the other atoms. Dispersion was accounted for using the D2 method^18^ and solvation effects^19^ were obtained using the lacvp* basis set. The dielectric constant chosen was 4.0 with the radii 1.40 Å.^12^ The Jaguar^19^ and Gaussian^20^ programs were used. The Gaussian program was used for the calculations of Hessians and optimization of the transition states. Jaguar was used in all the other calculations.

An important part of the calculations is to obtain values for the redox energies. Using pH = 7 and a redox potential for the electron donor of −1.5 eV, a reference value of 348.6 kcal/mol is obtained, the same as used for the previous studies of nitrogenase. A large part of the energy from the hydrolysis of two ATP, spent for every electron, leads to the low redox potential. A possible mechanism for saving almost the entire energy of the ATP hydrolysis and use it to help catalysis has been described elsewhere.^8^ In the experimentally suggested mechanism,^4−7^ there is no account for how the main part of the hydrolysis energy could be saved.^21^ A structural change was suggested to occur when the MoFe and Fe proteins bind, but that change would have to involve the cofactor to have any effect on catalysis. No such structural change has been reported on the cofactor even after detailed studies of the E_4_ state.^4−7^

The same model of the active state was used as in the study of the E_4_ state. The following amino acids outside the cofactor were included in the model: His195, Arg96, Arg359, Glu380, Phe381, Gln191, Val70, and Ser278, see Figure 1. These are the amino acids closest to the cofactor. Larger models have been investigated in previous studies^9,10^ but are considered not to be necessary for the present study.

For the binding of a water molecule in the water medium, an empirical value of 14 kcal/mol was used, the same as in all the previous studies. The same value is used for ammonia. The small entropy effect in the step where water comes from the medium and becomes bound is neglected.

## 3 Results

### 3.1 E_5_ State

At the end of the E_4_ state in the previous study,^12^ N_2_H_2_ is binding with one nitrogen bridging to Fe1 and Fe2, while the other nitrogen forms a single bond to Fe4. To form that structure, two water molecules were needed to bridge the proton transfer to N_2_H from S3A (incorrectly termed S1A in the previous paper). An important point, not mentioned in the previous paper, is that N_2_H_2_ has a large radical character with a spin of 0.76. When N_2_H_2_ has been formed, S3A is unprotonated. At the start of E_5_, a (H^+^, e^–^) couple is added with a proton on S3A. The addition is exergonic by −11.8 kcal/mol. The same spin-coupling as in the previous paper was used, with (−2, -4, -7). This means that Fe-atoms 2, 4, and 7 have negative spins, the other metal atoms have positive spins. The protonated sulfides are at this stage the belt sulfides S2B, S3A and S5A. Again, as in E_4_, there is a large radical character on N_2_H_2_ with a spin of 0.81. In the initial attempts for the mechanism in E_5_, the proton was moved directly from S3A to N_2_H_2_. That led to a very high barrier of 18.2 kcal/mol for the proton transfer. The reason is that the long distance between S3A and N_2_H_2_ requires that the bond between nitrogen and Fe1 is broken in the TS, which is very unfavorable. To search for a lower barrier, one water molecule was added, and it was found to bind to Fe4. In that process, the Fe4–N bond is broken. The water binding is endergonic by +2.4 kcal/mol.

**Figure 2:**
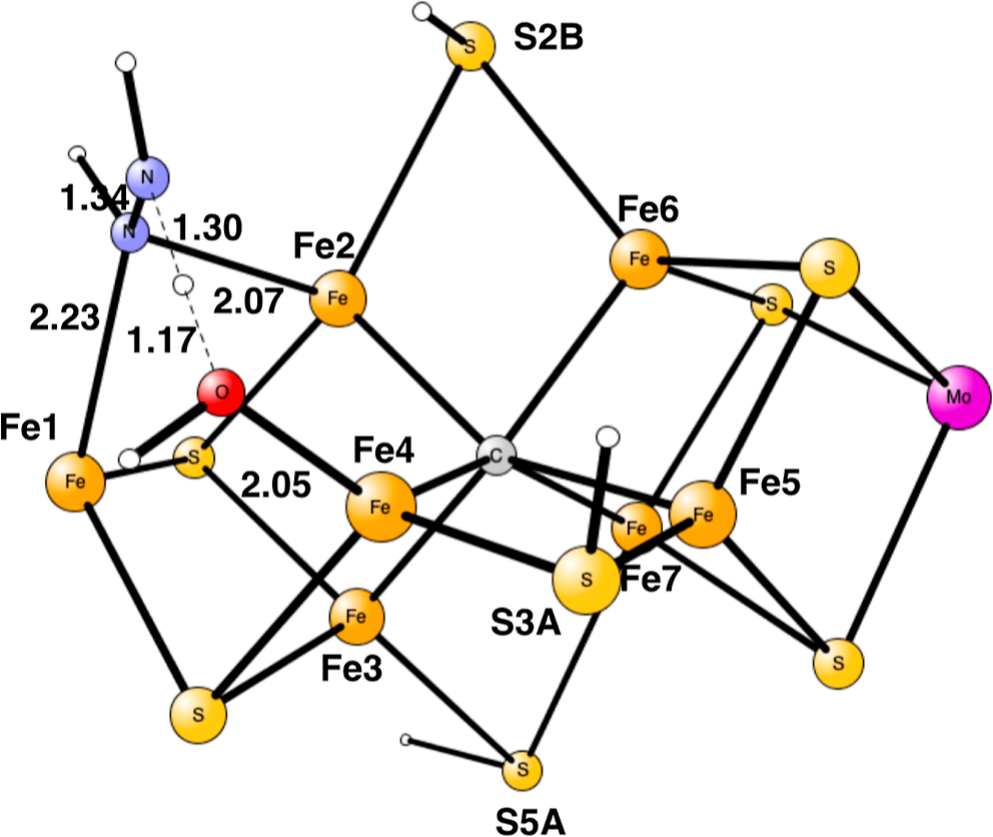
Optimized TS for proton transfer from H_2_O on Fe4 to N_2_H_2_ bound to Fe1 and Fe2 in E_5_. The imaginary frequency is *i*400 cm^–1^. There is a spin of −0.84 on the nitrogens. Distances are given in Å.

**Figure 3:**
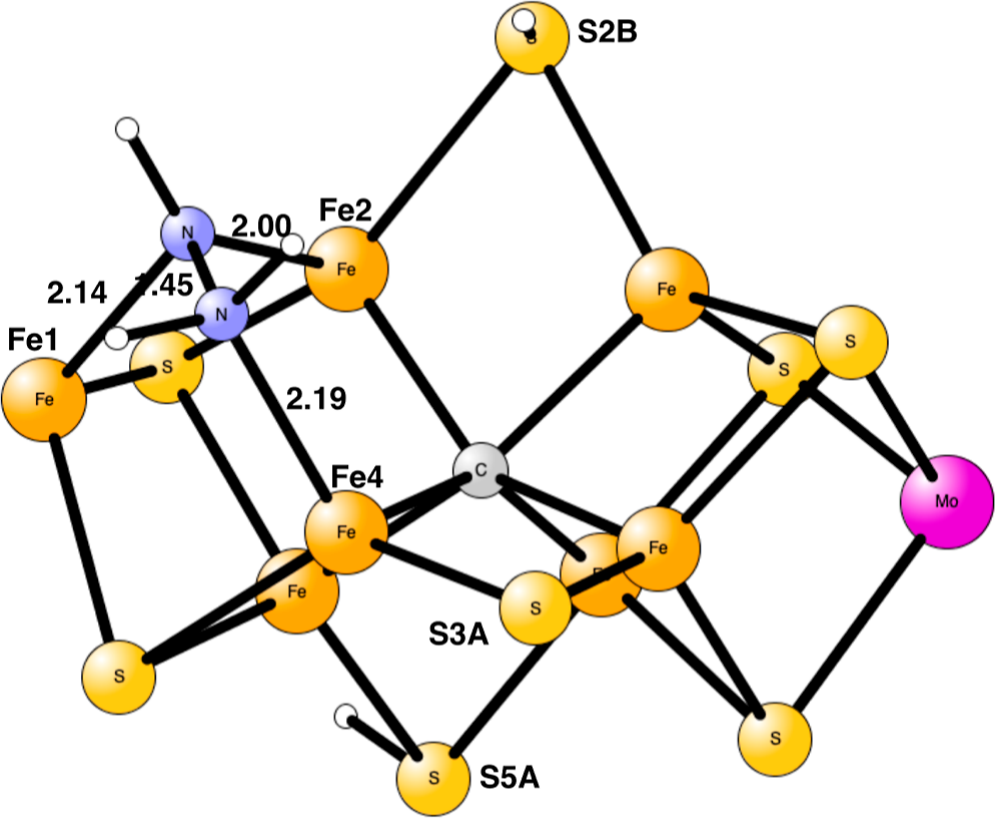
Optimized N_2_H_3_ structure in E_5_. There is no spin on the nitrogens.

In the TS obtained after a full optimization at the lacvp* level, shown in Figure 2, a proton leaves the water with an O–H bond distance of 1.17 Å. The H–N distance is 1.30 Å. After addition of the large basis set effect, solvent, dispersion and zero-point effects, the barrier actually becomes negative. The conclusion is that there is at most a very small barrier for this proton transfer. An exact value for the barrier is unimportant since it is so much lower than the maximum barrier allowed of 20 kcal/mol. Therefore, the total barrier is set to +2.4 kcal/mol from the cost to add water. The nitrogens still have radical character with a total spin of 0.84. The immediate N_2_H_3_ product has the water bound to Fe4 with a bond distance of 2.26 Å, and without a proton on S3A. The transfer of the proton is exergonic by −22.1 kcal/mol, including the cost of +2.4 kcal/mol for adding the water. N_2_H_3_ has at this stage no radical character. Before the E_4_ to E_5_ step is over, the added water leaves and N_2_H_3_ forms a structure where the imid nitrogen bridges Fe1 and Fe2, and the amino part from a bond to Fe4, see Figure 3. The entire E_4_ to E_5_ reaction step, leading to the formation of N_2_H_3_, is exergonic by as much as −37.9 kcal/mol, including the energy gain for the transfer of a (H^+^, e^–^) couple of −11.8 kcal/mol. It is interesting to note that N_2_H_3_ binds much better than N_2_H_2_ does. N_2_H_2_ has a large radical character while N_2_H_3_ does not. The alternative protonation of the nitrogen bridging between Fe1 and Fe2 was also studied. The product is then +3.1 kcal/mol higher than when the nitrogen bound to Fe4 is protonated.

### 3.2 E_6_ State

**Figure 4:**
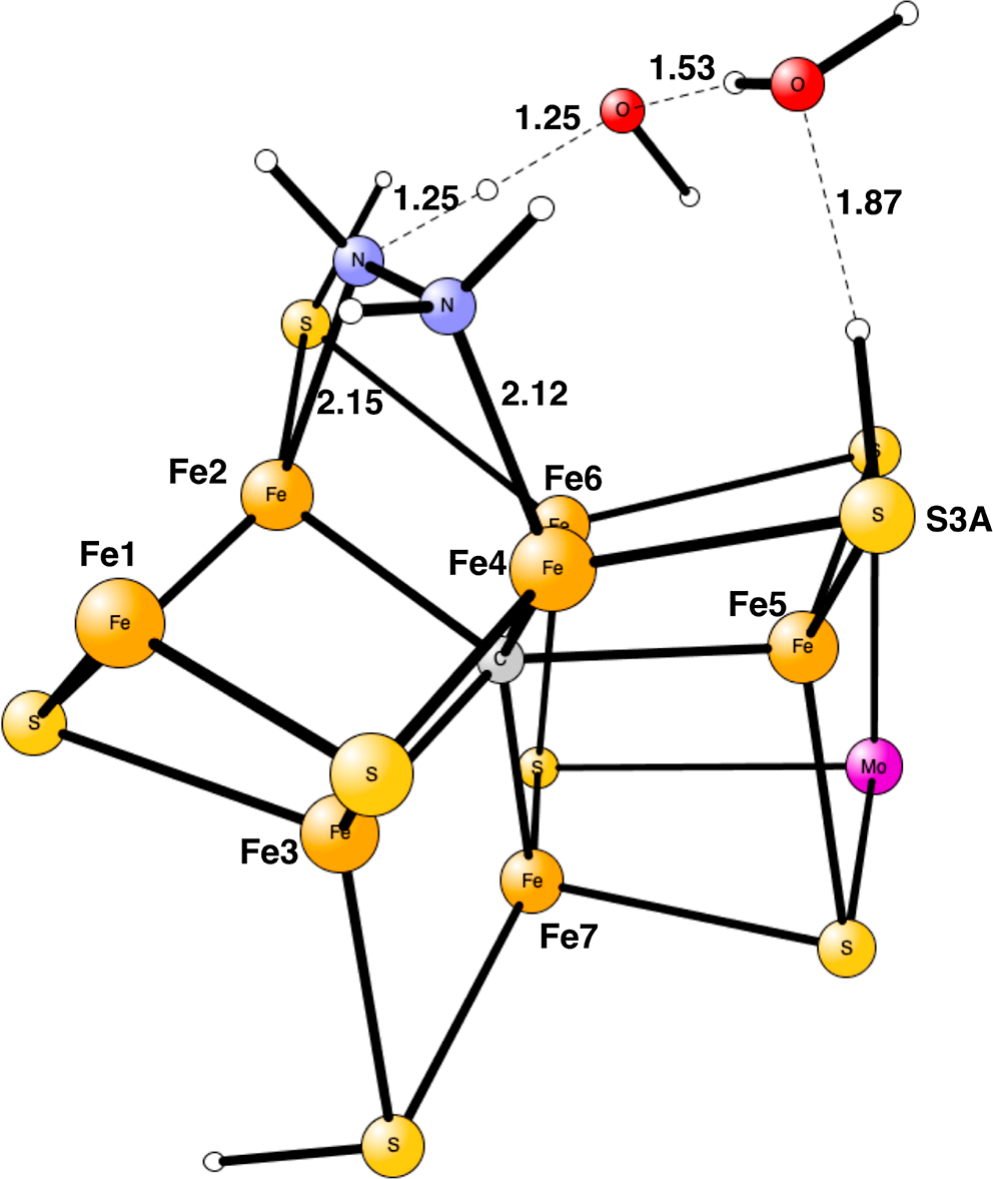
Optimized TS for proton transfer from S3A to N_2_H_3_ in E_6_ using two water molecules. The imaginary frequency is *i*113 cm^–1^.

**Figure 5:**
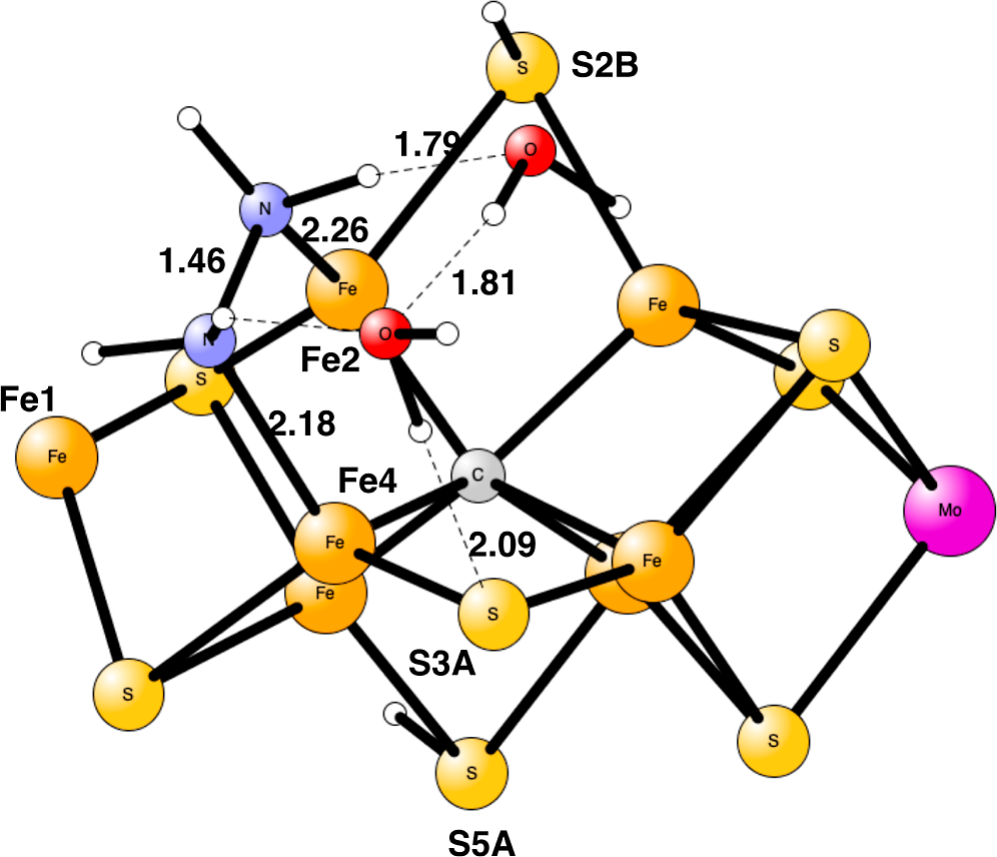
Optimized N_2_H_4_ structure in E_6_. There is no spin on the nitrogens.

At the start of E_6_, a (H^+^, e^–^) couple is added with a proton on S3A, which is endergonic by +1.2 kcal/mol. The same sulfides are now protonated as for E_5_. In order to describe the TS, two waters were added, which is exergonic by as much as −10.6 kcal/mol. The origin of the large gain of adding the two waters is that they form strong hydrogen bonds to the imide nitrogen with a N–H distance of 1.65 Å, and to the proton on S3A with a distance of 1.93 Å. The same spin-coupling as for E_5_ was used. Again, without adding water, the barrier for protonation of the substrate is too high. The simple reason is that the distance between S3A and the imide nitrogen is too long. Just as in the previous study on E_4_, the two water molecules bridges that distance, see Figure 4. After that, the barrier for moving the proton from S3A to N_2_H_3_ is very small, almost zero kcal/mol. As seen in the figure, the transfer of the two protons occurs concertedly in one step. The distance from the imide to the proton is 1.25 Å and from the proton to the water it is also 1.25 Å. Simultaneously, the distance from the first water to the oxygen of the second water is 1.53 Å, which is a significant shortening compared to a normal hydrogen bond distance. From the proton on S3A to the second water the distance is 1.87 Å, which is also much shorter than for a normal hydrogen bond. The efficiency of moving protons over water molecules is striking. The large exergonicity to protonate N_2_H_3_ to N_2_H_4_ is −26.8 kcal/mol, including the loss for protonating S3A of +1.2 kcal/mol and the gain to add the two water molecules of −10.6 kcal/mol. The two water molecules are strongly bound both at the beginning, with −10.6 kcal/mol, and at the end of the E_5_ to E_6_ transition, with −11.9 kcal/mol, and there is no spin on the nitrogens. The product is shown in Figure 5. The barrier is set to +1.2 kcal/mol for the protonation of S3A.

As mentioned above, the starting structure in E_6_ has the amino group of N_2_H_3_ bound to Fe4. If, instead, the amino group would be bound between Fe1 and Fe2, the structure would be +4.0 kcal/mol higher. The protonation of that structure was still investigated and the barrier was again quite low. The optimal product is the same in both cases.

### 3.3 E_7_ State

**Figure 6:**
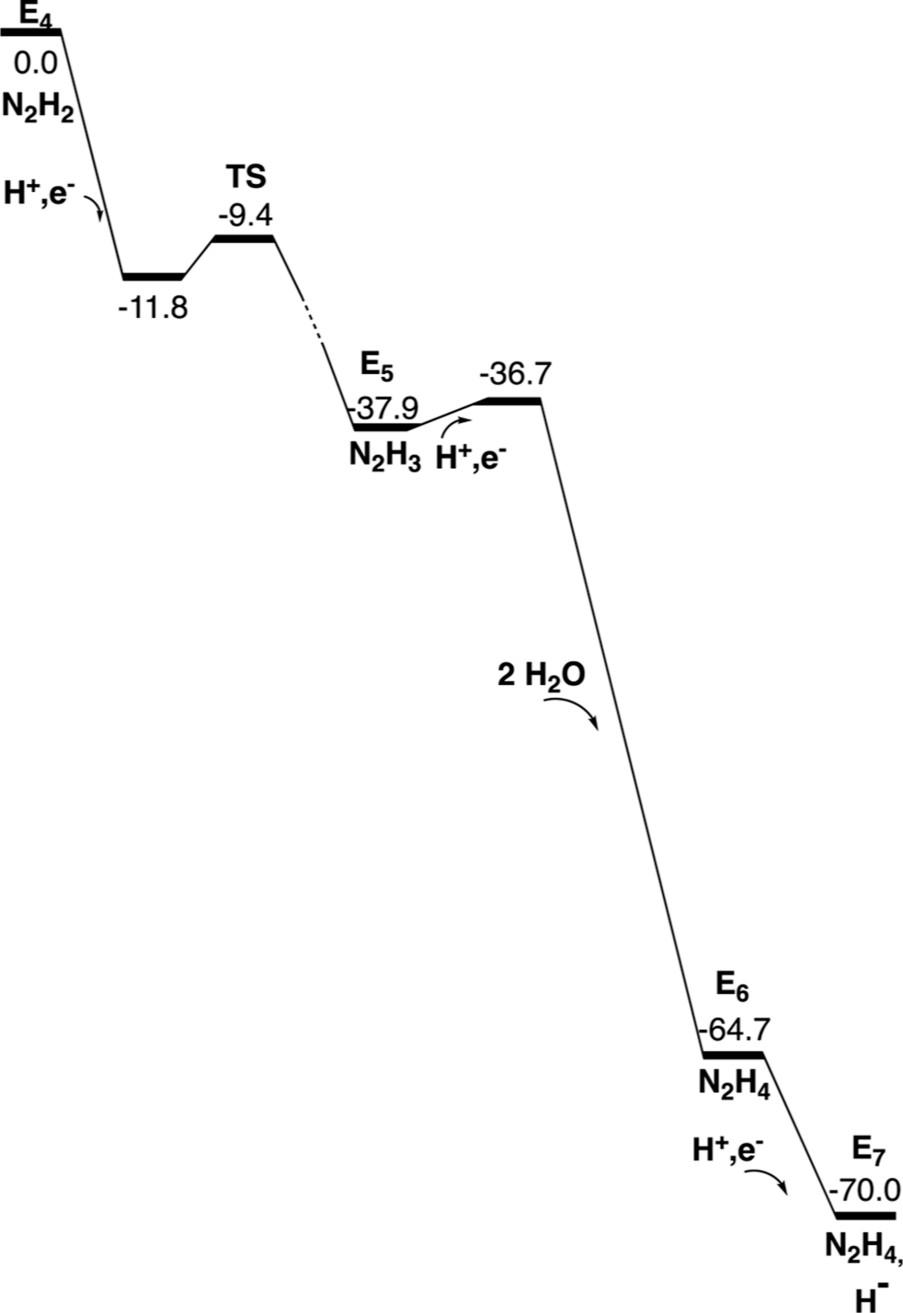
Free energy diagram for the steps from E_4_ to E_7._ Energies in kcal/mol.

In the E_6_ to E_7_ transition, there are a few alternatives for the addition of a (H^+^, e^–^) couple. The proton could either go to S3A as in the previous transitions, or it could form a hydride between Fe1 and Fe2. It could also go to N_2_H_4_ directly from the medium. The by far lowest energy is obtained for the hydride alternative. Again, it was found that the two water molecules are bound. However, they are bound by only 2.2 kcal/mol in E_7_, compared to 11.9 kcal/mol at the end of E_6_. The reason is that in E_7_, there is a competition between the binding of the hydride and the bond between Fe2 and one of the nitrogens in N_2_H_4_. The binding of the hydride forces the Fe2–N bond to be broken. It is 2.26 Å in E_6_ and over 5 Å in E_7_. As the Fe2–N bond increases the N–H bond becomes less polar and the hydrogen bonding to the two waters decreases. The addition of the (H^+^, e^–^) couple is still exergonic by −5.3 kcal/mol. For the other alternatives, adding the proton on S3A is +16.8 kcal/mol higher and adding it to N_2_H_4_ is +30.2 kcal/mol higher. The reason for the high energy for the S3A alternative is that an additional reduction of the metal part of the cofactor will force a Fe(I) contribution. For the same reason forming the N_2_H_5_ ligand is high in energy. The N_2_H_5_ energy goes strongly down if the N–N bond is broken, but the barrier is too high. The free energy diagram for the steps from E_4_ to E_7_ are shown in Figure 6. The very large exergonicity of the transitions are striking. The reason is the low redox potential of the donor. To go from E_5_ to E_7_ such a low redox potential is not needed, but it is absolutely necessary for climbing over the barrier for activating N_2_ in E_4_.

### 3.4 E_8_ State

**Figure 7:**
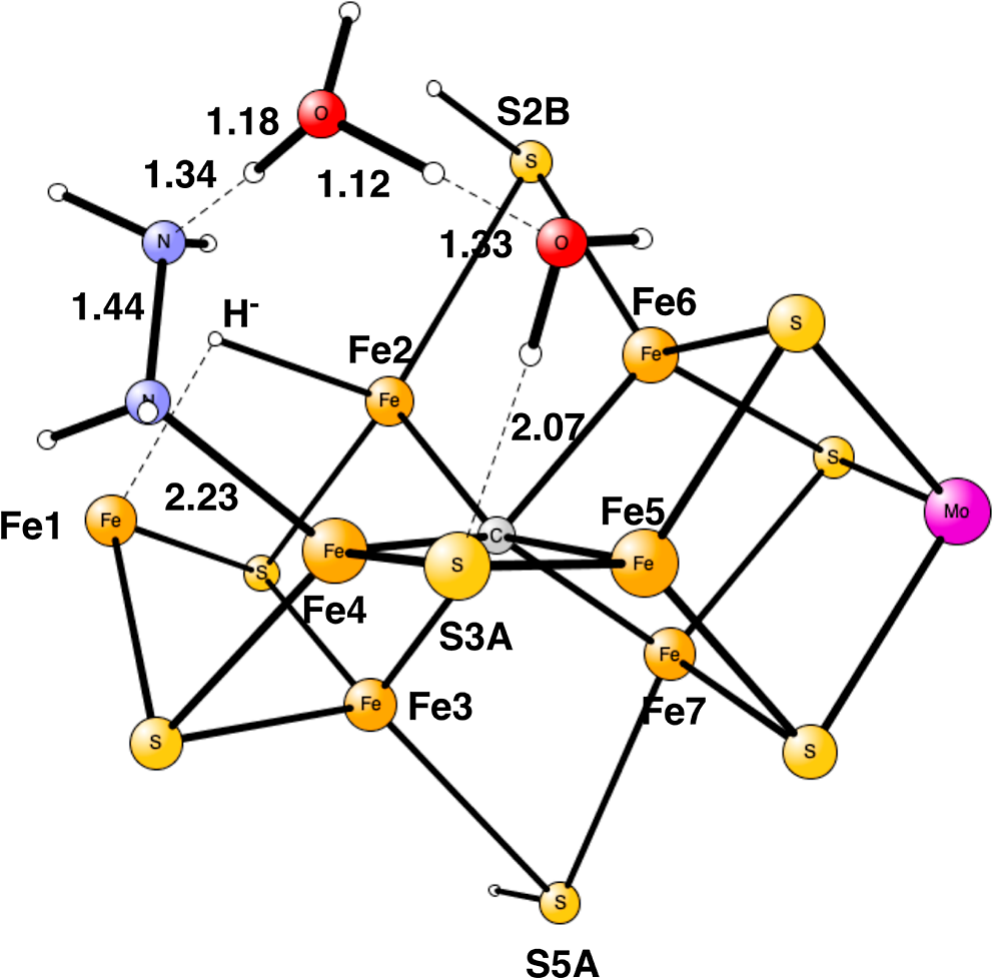
Optimized TS for proton transfer from S3A to N_2_H_4_ in E_8_.

The mechanism in the E_8_ state is the most complicated one for reasons discussed below. It starts out in the same way as in the previous states, with an addition of an (H^+^, e^–^) couple. The proton ends up on S3A as in E_5_ and E_6_. The addition is exergonic by −15.1 kcal/mol. The binding energy of the two waters is exergonic by −4.1 kcal/mol, which means that keeping the two waters from E_7_ has no extra cost. The two water molecules allow a transfer from S3A to N_2_H_4_ in a very similar way as in E_4_ and E_6_. The TS in Figure 7 shows a transient formation of H_5_O_2_. There is a short distance between the nitrogen and a proton in the first water of 1.34 Å, and also short hydrogen bonds between the water molecules. The barrier is rather low with +9.0 kcal/mol. However, the step is endergonic by +7.0 kcal/mol, which is not unexpected for forming a N_2_H_5_ ligand.

**Figure 8:**
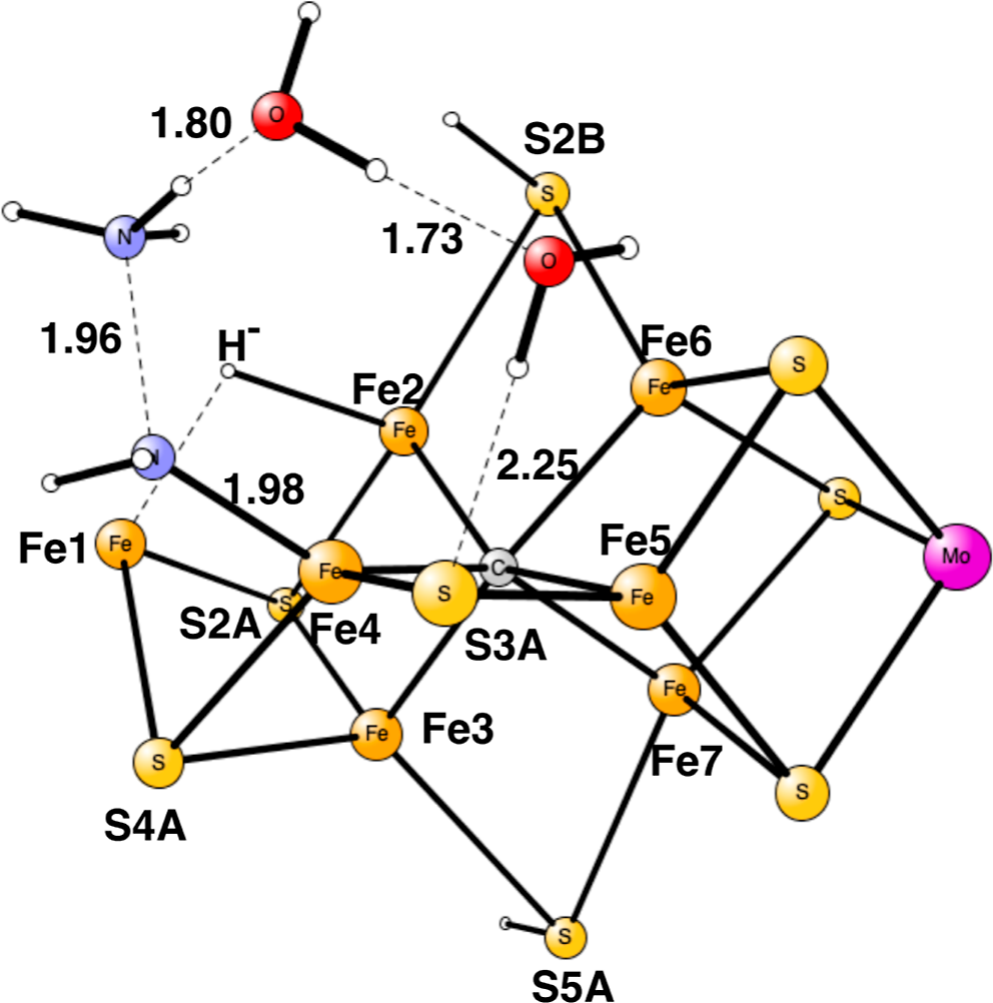
Optimized TS for N_2_ cleavage in E_8_.

**Figure 9:**
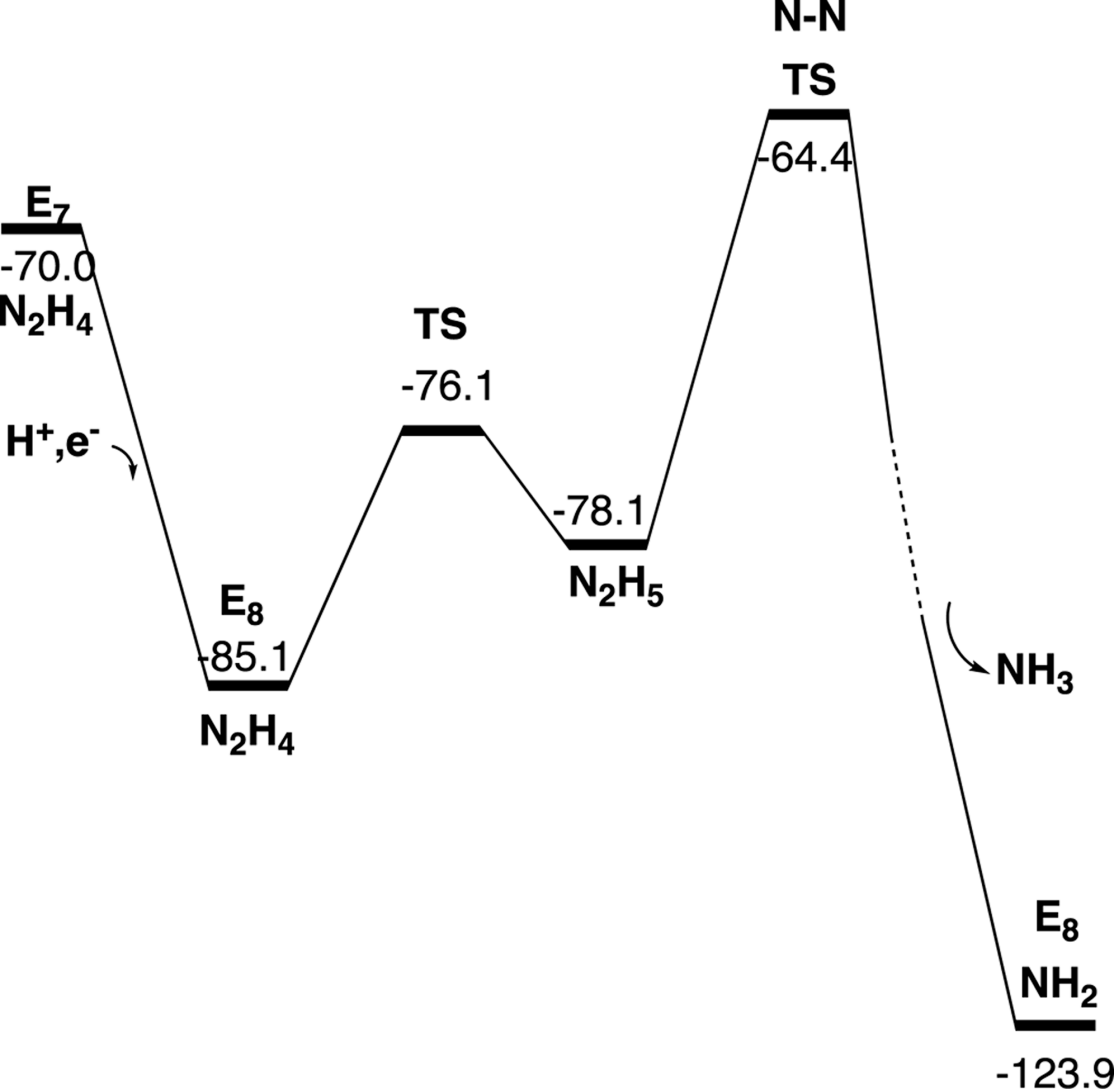
Free energy diagram for the steps from E_7_ to the step where the N–N bond is cleaved in E_8._ Energies in kcal/mol.

In the next step, the N–N bond is cleaved with a transition state shown in Figure 8. The local barrier is only 13.7 kcal/mol, but since the formation of the N_2_H_5_ reactant is endergonic by +7.0 kcal/mol, the total barrier becomes 20.7 kcal/mol. That is too high by 2–3 kcal/mol, which is very common for the present methodology. The reason is probably that multireference effects, not included in DFT, tend to be larger at high energies. It is the lowest barrier for N–N cleavage found in the present study. The N–N distance at the TS is 1.96 Å. There are not any very short hydrogen bonds involving N_2_H_5_ or the waters. The cleavage of the N–N bond is very exergonic. Including the gain of ammonia binding in the water medium, here taken to be the same as for a water in water of −14 kcal/mol, it leads to an exergonicity of −45.8 kcal/mol. From this point on, the water molecules do not participate in the reactions. The free energies for the steps from E_7_ to formation of the first NH_3_ are shown in Figure 9.

The problem to complete the catalytic cycle starts at this point. After the N–N cleavage, one NH_3_ is released and the remaining amino group is bridging between Fe1 and Fe4. All the (H^+^, e^–^) couples have now already been added, which means that the proton on S5A has to move in some way to the amino group. The pathway for that process turned out to be very difficult to find. The first attempts were made to move the proton on S5A to S3A using bridging water molecules. However, adding water molecules in the required region is very endergonic and the barrier for the proton transfer is, therefore, too high.

In the second attempt, the proton on S5A was moved to S2A, since S2A was found in a previous study to be quite easy to move around.^12^ Indeed, the barrier for the proton transfer is very low with only +7.5 kcal/mol. S2A loses its bond to Fe1 in the process. However, barriers for moving the proton further from S2A toward the amino group were all found to be too high.

**Figure 10:**
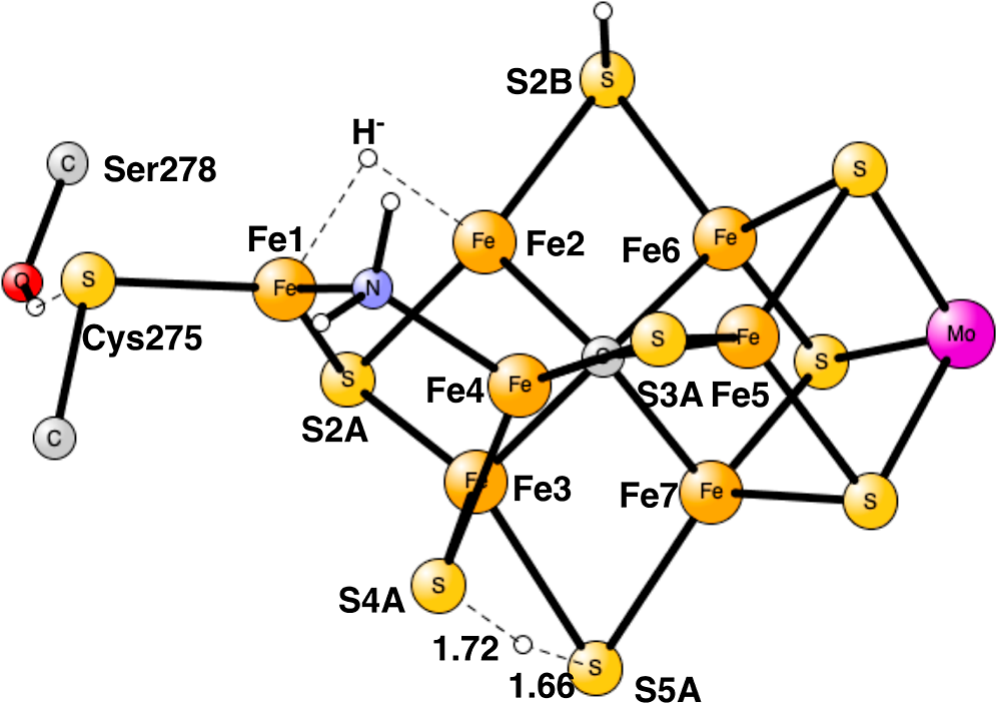
TS for proton transfer from S5A to S4A in E_8_.

In the next attempt, the proton was moved from S5A to S4A, and that turned out to be the best pathway. The first proton transfer, from S5A to S4A, was found to have a barrier of +14.9 kcal/mol, significantly higher than the one for S2A of +7.5 kcal/mol, but it is still sufficiently low. The TS is shown in Figure 10. At the TS, S4A has lost its bond to Fe1. The distances for the proton to S4A and S5A are 1.72 and 1.66 Å, respectively. The proton transfer is quite endergonic by +9.3 kcal/mol.

**Figure 11:**
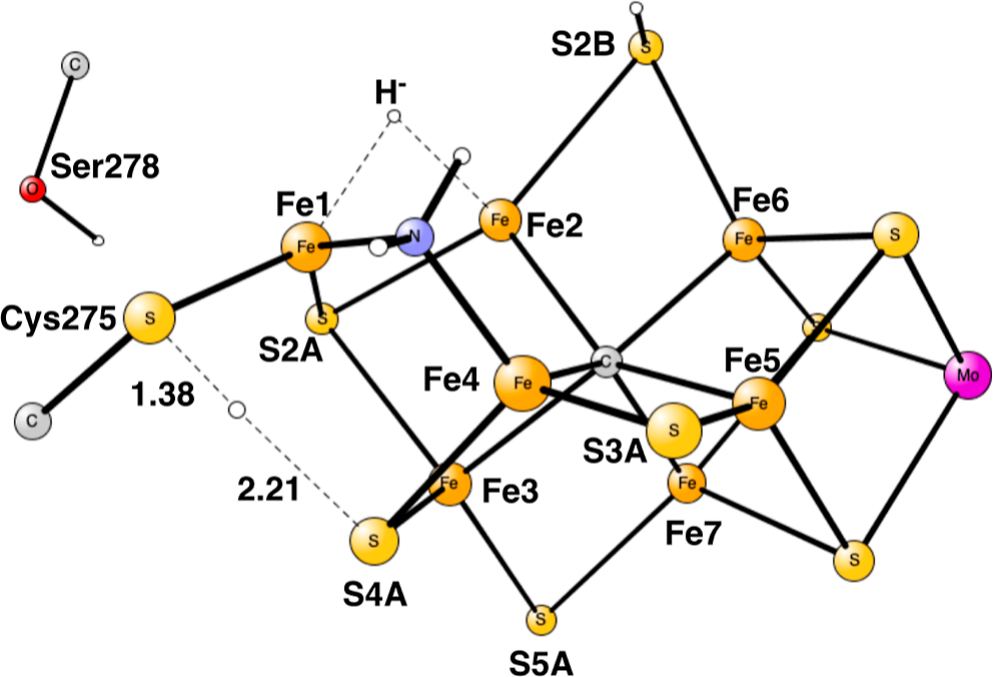
TS for proton transfer from S4A to Cys275 in E_8_.

In the next step, the proton on S4A needs to be moved closer to the amino group. The best pathway found is to move it to Cys275. In the TS, shown in Figure 11, the CysS–H distance is 1.38 Å, while the S4A–H distance is much longer with 2.21 Å, indicating a late TS. The local barrier is very small with all corrections included, only +0.8 kcal/mol. But since the previous step was endergonic by +9.3 kcal/mol, the total barrier with respect to the resting state becomes +10.1 kcal/mol. The product with Cys275–H^+^ is +10.3 kcal/mol above the resting state with all corrections included. It should be clarified that the TS optimization is performed using the lacvp* basis set without solvation and dispersion, which may lead to a higher final energy for the reactant than for the TS. Also, the zero-point energy for the TS is −0.8 kcal/mol lower than for the preceding local reactant.

**Figure 12:**
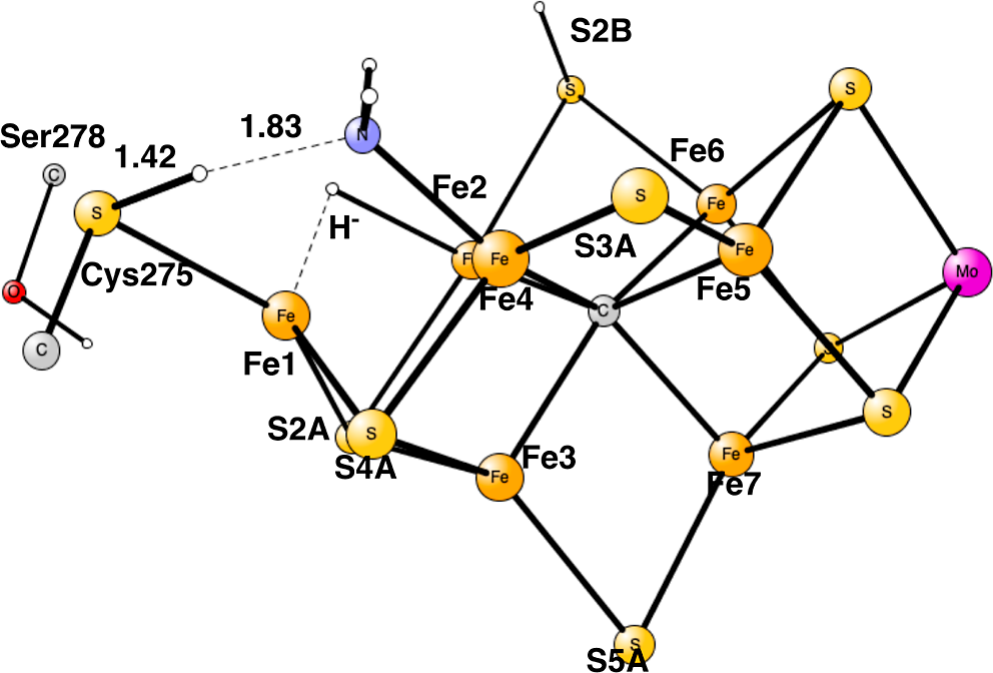
TS for proton transfer from Cys275 to NH_2_ in E_8_.

Only one more step is needed. To move the proton from Cys271 to NH_2_ goes over the TS shown in Figure 12. As seen in the figure, the TS is quite early with an S–H^+^ distance of 1.42 Å compared to a normal S–H^+^ bond of 1.35 Å. Again, the local barrier is quite low with +5.7 kcal/mol. The total barrier from the resting state with S5A–H^+^ is then 16.0 kcal/mol. The product with a bound NH_3_ is 16.7 kcal/mol below the resting state.

The NH_3_ product is very weakly bound by 2.6 kcal/mol compared to being in the water medium. It is bound mainly by dispersion to the cofactor. The binding of NH_3_ in the surrounding water medium is taken to be 14 kcal/mol, see above. NH_3_ should be easily released before the next cycle of catalysis.

**Figure 13:**
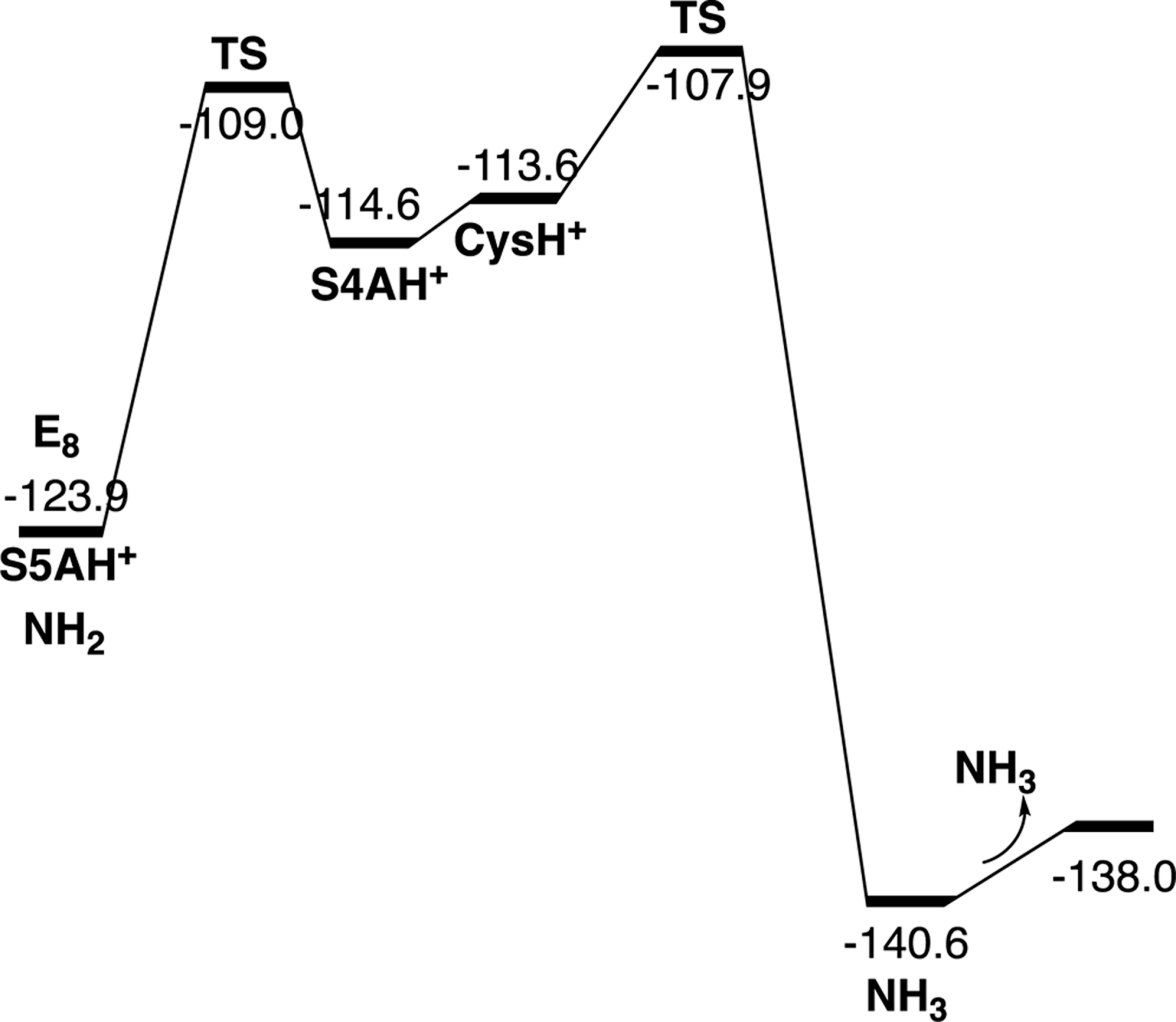
Free energy diagram for the steps in E_8_ from the formation of NH_2_ to the release of the second NH_3_. Energies in kcal/mol.

After the release of the second ammonia, the cofactor is back to the lowest energy E_0_ state, which has a hydride bound between Fe1 and Fe2, and one protonated sulfide, S2B. The free energies for the last steps in E_8_ leading to the formation of the second NH_3_ are shown in Figure 13.

## 4 Conclusions

In a previous study, the E_4_ state^12^ in which N_2_ is activated, was studied. Perfect agreement with the results from EPR measurements were found. In contrast, for the experimentally suggested structure and mechanism, very big errors of 40 kcal/mol were found.^11^ The present study is a continuation of the study of the E_4_ state for the subsequent reduction steps from E_5_ to E_8_. The starting point in E_5_ is the N_2_H_2_ structure obtained at the end of E_4_.

Each transition starts with an addition of a (H^+^, e^–^) couple. Except for the E_7_ state, the added proton was found to be initially placed at S3A. For E_7_, it was found to be most favorable to obtain a hydride bound between Fe1 and Fe2. In all the E-states, it was found that water molecules were needed for the transfer of protons to the substrate, just as was the case in E_4_. In E_5_, the addition of one water was enough to bridge S3A and N_2_H_2_. The water binds temporarily on Fe4. In E_5_, E_6_ and E_8_, two waters had to be added, much like they did in E_4_. The barriers for moving the protons to the substrate are very small, in E_5_ and E_6_ almost nonexisting. Without waters they are, instead, very high, almost impossible to climb. In the E_8_ state, the barriers are larger but not impossible to overcome, all less than 16 kcal/mol.

E_8_ was the most complicated state to describe. The main reason is that all the protons except the one on S2B had to be used to protonate the substrate in order to return to E_0_. To move the proton on S5A was most complicated since it is far from the substrate at that stage. After many attempts a pathway was found and the catalytic steps were completed. The N–N bond was broken in E_8_ and the two ammonia molecules were formed and released afterward in the same transition. The barrier for N–N cleavage was found to be quite high, 20.7 kcal/mol, which is a slight overestimation. Energy diagrams for the transitions from E_4_ to E_8_ are given in Figures 6, 9 and 13. The E_5_ to E_8_ steps are strongly exergonic due to the very low redox potential of the donor, which is required for E_4_ but not for the higher states.

Finally, it should be said that since there are so many details in the mechanism, alternative, better pathways may exist, which have not yet been found. For example, since the cofactor has a high symmetry, symmetry related pathways are certainly possible. However, it is important at the present stage to show that there exists at least one possible low energy path from E_4_ to E_8_.

## Supporting Information Available

Coordinates for all structures (PDF)
