# Supplementary material for: Final E5 to E8 Steps in the Nitrogenase Mechanism for Nitrogen Fixation
Source: J Phys Chem B. 2024 Sep 30;128(40):9699–705. doi: 10.1021/acs.jpcb.4c04331 (PMC11472303; doi:10.1021/acs.jpcb.4c04331)
Supplement: Supplementary file 1 — jp4c04331_si_001.pdf [file jp4c04331_si_001.pdf]

Supporting Information:

The Final E_5 to E_8 Steps in the Nitrogenase Mechanism for Nitrogen Fixation.

Per E. M. Siegbahn*

Department of Organic Chemistry, Arrhenius Laboratory, Stockholm University,

SE-106 91, Stockholm, Sweden. Email:per.siegbahn@su.se

The E_4 structure in **Figure 1.** # means fixed atom

Mo1	16.3411878090	-5.8843224691	53.3532482045
Fe2	9.8545237449	-8.7066672616	55.8026803599
Fe3	11.1270830736	-6.1567857625	55.2253963209
Fe4	11.2975412550	-7.8791910044	53.1939275092
Fe5	12.4004711229	-8.7721346694	55.7826411927
Fe6	14.5828727439	-8.0468881788	54.3097782500
Fe7	13.9710400141	-5.7325498335	55.4475370195
Fe8	13.2849575036	-6.0681983570	52.9644078942
C9	17.6826441859	-2.2589053299	54.3195294106
H10	17.8384919968	-1.2545925665	53.9210344378
H11	16.6046985230	-2.3860228098	54.4827461332
C12	18.1490916089	-3.3294888351	53.3066023869
C13	19.6205941297	-3.0741902320	52.9009842022
H14	20.2451659748	-3.1818351138	53.7935819454
H15	19.6926868002	-2.0373160710	52.5549727621
C16	20.1256658531	-4.0148389529	51.7871779042
H17	19.6151226762	-3.8053958370	50.8451575598
H18	19.9209928568	-5.0510016584	52.0769603817
C19	17.3109458328	-3.4189019885	51.9976756549
O20	16.8868131451	-4.6179283585	51.7026256488
O21	17.1890581818	-2.4195844689	51.2905840319
O22	18.0371388030	-4.6068516786	53.9600244506
C23	12.6703429290	-7.2370369276	54.4920100332
S24	12.3157255346	-4.6567520170	56.8687276896
S25	16.0758574362	-6.7283949943	55.6923376397
S26	9.4625045707	-4.9381587745	53.9288238078
S28	14.4626583560	-10.1118021486	55.6799683710
S29	14.4754472099	-4.1580799740	53.6650878874
S30	10.0380570449	-9.7078565374	53.6279410778
S31	15.0144651125	-7.5224654096	52.0632631740
S32	11.6130691052	-6.5318388603	51.1209386392

H33	13.6351742031#	-2.1902105282#	49.0425559854#
C34	13.2979245689#	-2.0241172350#	50.0550231810#
H35	13.9034573660	-2.6598888494	50.7056694277
H36	13.4458796854	-0.9759837742	50.3384609648
N37	11.8837648438	-2.4084978367	50.1233956643
H38	11.2953624729	-2.0817310269	49.3700215198
C39	11.2184954364	-2.8361532489	51.2147486715
N40	11.7993980766	-3.0468706509	52.3782661061
H41	11.2465556981	-3.5295639622	53.1004721498
H42	12.8174814132	-3.1088642410	52.5167372597
N43	9.8636686496	-2.9870417022	51.1220582283
H44	9.5059159338	-3.2738784432	50.2198237743
H45	9.4353741924	-3.4837118621	51.9147680080
H46	7.9311775757	0.3969127240	59.4142474362
C47	8.3601754768	-1.3536927130	60.6733970028
H48	8.2198839164	-1.5872878048	61.7357893086
H49	7.5979341439	-1.9169598563	60.1227019719
C50	9.7342740982	-1.8208202775	60.2990237029
N51	10.7434350030	-1.8645251239	61.2445204061
C52	10.2173002769	-2.2368975484	59.0790733622
H53	9.7528195690	-2.3761937290	58.1160708509
C54	11.8067447778	-2.3077230738	60.5997409865
H55	12.7794232071	-2.4938842201	61.0333020138
N56	11.5445812744	-2.5368910735	59.2853935335
H57	19.2247459747	-4.4001181294	57.4827423718
H58	16.7207626868	-8.8606035832	50.7823707470
C59	18.3600442894	-7.4209545971	50.6554863816
H60	17.7203439277	-6.5342444729	50.6265155384
H61	19.1973292499	-7.2324642976	49.9691587286
C62	18.9076235659	-7.5720035941	52.0408482857
N63	18.1761048537	-7.2359640103	53.1707608275
C64	20.1357264525	-8.0388899954	52.4430320023
H65	20.9854226467	-8.3939914895	51.8805623874
C66	18.9398448911	-7.4917565622	54.2223093263
H67	18.6892631800	-7.2983533025	55.2536852542
N68	20.1310839156	-7.9892042367	53.8265468999
H69	20.8990905948	-8.1757620497	54.4545171469
H70	6.9632433529	-10.5490125668	56.9226286093
C71	6.1750521330#	-9.8249632781#	56.6900008432#
H72	5.5309558047#	-10.2322288176#	56.0424860987#
H73	5.6932327346#	-9.5371417035#	57.6127207581#
S74	8.1208576459	-7.9022504903	57.1032182752
H75	12.1562091462	-2.9523415094	58.5858874360
H76	18.2516973875	-4.4020611156	54.9525044855
C78	18.3652903901	-2.3659105397	55.7119717248
O79	18.6559616188	-3.5646337195	56.1110449935
O80	18.5502542807	-1.3253137664	56.3655395988
C81	21.6036263071	-3.8760164504	51.5491089153
O82	22.1579211357	-3.4773493276	50.5434515634
O83	22.3208176811#	-4.2658020416#	52.6398596358#

H84	15.7024656862#	-0.1996317006#	61.4912312798#
C85	14.8549934097#	-0.0829911871#	60.8320021209#
H86	13.9165015895#	-0.2867338793#	61.3260937710#
H87	14.8279483830	0.9915817637	60.5848765406
C88	15.0599857654#	-0.8751944034#	59.5441990369#
H89	14.3835077641	-0.4989717751	58.7665435195
H90	14.7960412608	-1.9266333706	59.7063201181
C91	16.5031908365	-0.8295814611	59.0342498621
H92	16.9263363375	0.1792276503	59.1532367752
H93	16.5564164622	-1.0382358650	57.9599109557
C94	17.4223544696	-1.8256625768	59.7544369704
O95	17.0868747364	-2.4202248520	60.7847792022
N96	18.6426525153	-1.9792378187	59.1743279626
H97	19.1605083577	-2.7954550928	59.4787839270
H98	18.7530174722	-1.7209760024	58.1836447215
C99	10.6519926369#	0.0100033143#	53.3460014096#
H100	11.1636988658#	0.9592721141#	53.2872696758#
H101	9.5863320405#	0.1850132805#	53.3579375021#
H102	10.9190941953	-0.5834696714	52.4698598363
C103	11.0702392862	-0.6567050972	54.6716574163
H104	10.7268855377	-1.6990700515	54.6626107529
C105	10.3874199892	0.0520433737	55.8508273284
H106	9.2941105711	-0.0085625901	55.7743167664
H107	10.6617587762	1.1164166532	55.8783296763
H108	10.6800093892	-0.3902336710	56.8090000043
C109	12.5945539536	-0.6513049755	54.8507560345
H110	13.1103480538	-1.1313470011	54.0130682885
H111	12.8894572896	-1.1896090156	55.7581333620
H112	12.9697410042	0.3790919023	54.9313980916
H113	13.4550779485#	-12.2303887427#	53.1883137695#
C114	13.9110016728#	-11.6210552681#	52.4219872140#
H115	14.6576041196	-10.9550734117	52.8624419645
H116	14.3408188816	-12.2308993538	51.6231944423
N117	12.7296192230	-10.8923147133	51.9338144653
H118	11.9410187228	-10.7991659610	52.5954798966
C119	12.6212753307	-10.1560446683	50.8405302031
N120	13.7167480288	-9.8445348858	50.1013882126
H121	13.5281926408	-9.2378159386	49.3113290911
H122	14.5084804678	-9.4951839573	50.6517771371
N123	11.3879907874	-9.7805114819	50.4131415055
H124	10.6406278249	-9.9925322111	51.0801011599
H125	11.3275121517	-8.7969058836	50.1340149323
H126	19.2019475911	-8.2163304887	61.7093235311
C127	19.5978933129#	-7.2298795947#	61.9900500755#
H128	20.6406211045	-7.3641195038	62.2959595225
C129	18.8344260642	-6.6814960099	63.1941988421
O130	19.3879045594	-6.1891041430	64.1746597346
C131	19.4918720166#	-6.2717980482#	60.7990634787#
H132	19.8694283581	-5.2838229886	61.0857811629
H133	18.4386122989	-6.1229671825	60.5398528431

C134	20.2371381416	-6.7782439484	59.5494357355
H135	20.0032393899	-7.8335889829	59.3637796029
H136	21.3241023671	-6.7419869233	59.7148158998
C137	19.9683223809	-6.0624745740	58.2188044511
O138	20.2354348347	-6.5877909236	57.1480801485
O139	19.4567873948	-4.8433600258	58.3627774851
N140	17.4797149232	-6.7629628212	63.0492541790
H141	17.1210565606	-7.0987283314	62.1637624911
C142	16.5368854076#	-6.0058843383#	63.8530414920#
H143	15.8865455666	-6.6972524048	64.4083744961
H144	17.1205535772	-5.4402388817	64.5836011345
C145	15.6691451794	-5.0681382096	62.9801187655
H146	16.2844349341	-4.2474953653	62.5958149885
H147	14.8984296854	-4.6249419249	63.6254221775
C148	15.0285031061	-5.8029697076	61.8160639212
C149	13.9878756299	-6.7249763181	62.0183930931
H150	13.5859771134	-6.8694631672	63.0200441311
C151	15.5168434008	-5.6198177031	60.5116791622
H152	16.2911891507	-4.8764291974	60.3369114161
C153	13.4543795337	-7.4489764885	60.9501748969
H154	12.6401173417	-8.1487283297	61.1207050466
C155	14.9912354408	-6.3498437026	59.4395958371
H156	15.3801018175	-6.1966463631	58.4367901867
C157	13.9604063090	-7.2683739588	59.6584325686
H158	13.5459782377	-7.8339105804	58.8275632984
H159	9.0037560472#	0.7050733335#	60.7752693345#
C160	8.1269803484#	0.1560803014#	60.4649996132#
H161	7.2836720063#	0.4592420217#	61.0677700037#
H268	8.5766101054	-4.5759831878	54.8844187042
H269	18.2592781145#	-9.4987529070#	50.1505401512#
C270	17.5839755707#	-8.6559201270#	50.1520260987#
H271	17.2773626234#	-8.4419892695#	49.1387987548#
H273	23.2543263111	-4.1142159740	52.4015686380
H274	10.4518484760	-5.9043092316	51.4249389337
H275	15.1465914384	-9.5512893930	56.7013887979
H277	12.2829812034	-5.5530729437	57.8783241365
H278	11.1931465340	-9.7873804705	56.5321306707
H279	10.9167755393	-7.4658591505	56.4837484366
C280	6.7703109854	-8.5758097809	56.0541926573
H281	5.9982599804	-7.8042500554	55.9381843529
H282	7.1567885251	-8.7950033546	55.0517510584
H283	7.1493941107#	-5.8354098952#	59.8218942714#
C284	6.9590149674#	-4.8009333484#	59.5000035989#
H285	5.9039260033#	-4.4903549879#	59.5181449819#
H286	7.4684022920	-4.1801929256	60.2526418680
C287	7.5586458473	-4.4488296119	58.1343715581
H288	8.6201961954	-4.7324350866	58.1076955378
H289	7.5129755415	-3.3587528818	58.0098877403
O290	6.8623416785	-4.9995052066	57.0275377134
H291	7.2185473731	-5.9102330485	56.9244449392

The starting E_5 structure with N_2H_2

Energies: E= -8030,392003, solv = -0.206498, disp = --238.55 Z_0 = 901.09

Mo1	16.6391645900	-5.5358724094	53.1081452353
Fe2	9.5291035018	-7.1354975379	55.2494596239
Fe3	11.6924940975	-5.3868596138	55.4389185770
Fe4	11.2801827556	-6.8683709694	53.1131187909
Fe5	12.3653447359	-8.2682682210	55.1418314105
Fe6	14.8201532799	-7.5990387842	53.9834167045
Fe7	14.3467522936	-5.2388256497	55.0435345701
Fe8	13.5031468631	-5.5946454257	52.4926679672
C9	17.8932893443	-1.9820697875	54.2298676648
H10	18.0590132687	-0.9553007590	53.8978074682
H11	16.8102927554	-2.1195014321	54.3388333042
C12	18.4112067121	-2.9912717141	53.1802723000
C13	19.8830141248	-2.6918520416	52.8158146761
H14	20.4979471323	-2.9176814325	53.6919198273
H15	19.9720528310	-1.6218739079	52.6003180120
C16	20.3750498345	-3.4952763107	51.5900993089
H17	20.0205759360	-3.0426357806	50.6628611397
H18	19.9770179881	-4.5141558214	51.6554073687
C19	17.6353937149	-3.0722060030	51.8284647076
O20	17.3081495175	-4.2861859847	51.4764740995
O21	17.5087881293	-2.0595025901	51.1428568979
O22	18.3254556025	-4.2996084999	53.7776251563
C23	12.9231046010	-6.5114595927	54.2022547455
S24	13.1927784729	-3.9370382615	56.7001713031
S25	16.3467814280	-6.4082935412	55.4210013381
S26	9.7419234238	-5.0945561181	53.9993976166
S27	14.3680457156	-9.6809994714	55.2751264667
S28	14.8209670662	-3.7519229820	53.2223618631
S29	10.4171616506	-8.9939310944	53.8525965114
S30	15.2270476387	-7.0947926972	51.7519354681
S31	11.8260658243	-6.1654741904	50.7163868427
H32	13.6351742178#	-2.1902104610#	49.0425559793#
C33	13.2979244126#	-2.0241175125#	50.0550234581#
H34	13.8925283878	-2.6754525474	50.7006756132
H35	13.4829103039	-0.9818881898	50.3391288072
N36	11.8734254690	-2.3592479550	50.1588964583
H37	11.2714832892	-2.0500438607	49.4090238386
C38	11.2456825732	-2.7994403937	51.2651575002
N39	11.8604313473	-2.9795642301	52.4190864506
H40	11.3658195230	-3.5197795198	53.1410606767
H41	12.8831986702	-2.9177451634	52.5371466186
N42	9.8972768572	-3.0098652049	51.2114195455
H43	9.5187521971	-3.2898047823	50.3154932686
H44	9.5183092509	-3.5414547937	52.0047137481
H45	7.9307518705	0.3892129180	59.4126686150

C46	8.3345145422	-1.3518535660	60.6897984160
H47	8.2615979423	-1.5633111444	61.7633855601
H48	7.5209502981	-1.9027677066	60.2022161982
C49	9.6677887732	-1.8491282934	60.2310986150
N50	10.7104854170	-1.9984624732	61.1260195605
C51	10.0893497828	-2.1622775839	58.9592149743
H52	9.5808108603	-2.1932592472	58.0083982016
C53	11.7376644821	-2.3973099604	60.3989772365
H54	12.7265597675	-2.6263156690	60.7707954567
N55	11.4152546040	-2.5059308394	59.0824217142
H56	19.2692465872	-4.3350595630	57.4750686447
H57	16.7458442574	-8.8128119467	50.8212041883
C58	18.4288232157	-7.4105642383	50.5273834246
H59	17.8209394226	-6.5046928916	50.4549179829
H60	19.2230118365	-7.3161642025	49.7733476252
C61	19.0645509102	-7.4551559465	51.8796214087
N62	18.4222228168	-6.9648203663	53.0059071016
C63	20.2890542487	-7.9502303846	52.2571730691
H64	21.0825683464	-8.4114229894	51.6896163639
C65	19.2312195439	-7.1612947733	54.0359325116
H66	19.0493260550	-6.8680738293	55.0583896420
N67	20.3692559819	-7.7636015737	53.6271642928
H68	21.1564808554	-7.9513868549	54.2298535248
H69	6.9576023341	-10.5538225909	56.9280794445
C70	6.1750544256#	-9.8249633458#	56.6900008379#
H71	5.5309554300#	-10.2322282065#	56.0424860875#
H72	5.6932325302#	-9.5371412395#	57.6127205064#
S73	8.1449206566	-7.8815247776	56.9671562997
H74	12.0232586575	-2.8136414595	58.3216650268
H75	18.5183826396	-4.1414057542	54.7871792936
C76	18.5103308112	-2.1545098373	55.6491827036
O77	18.8197964097	-3.3614289067	56.0051735193
O78	18.6271842046	-1.1386743297	56.3583110943
C79	21.8684711926	-3.5935190058	51.5470864781
O80	22.6330468034	-3.1682274237	50.7030862695
O81	22.3208171256#	-4.2658019828#	52.6398596068#
H82	15.7024655258#	-0.1996319937#	61.4912314345#
C83	14.8549937991#	-0.0829907271#	60.8320018999#
H84	13.9165015893#	-0.2867338795#	61.3260937710#
H85	14.8261678791	0.9914058334	60.5839274321
C86	15.0599854193#	-0.8751945501#	59.5441990489#
H87	14.3507924314	-0.5364509354	58.7788039335
H88	14.8471394359	-1.9357828923	59.7190018292
C89	16.4887847223	-0.7672554851	59.0013502931
H90	16.8561800480	0.2672906829	59.0753479753
H91	16.5317260446	-1.0133128460	57.9343449632
C92	17.4771456002	-1.6804036906	59.7370498344
O93	17.1937485071	-2.2535120099	60.7965597735
N94	18.6914710008	-1.7910253763	59.1388760905
H95	19.2605134381	-2.5608054060	59.4707873552

H96	18.7865140956	-1.5457246388	58.1407126816
C97	10.6519926894#	0.0100029132#	53.3460013809#
H98	11.1636988581#	0.9592721262#	53.2872698035#
H99	9.5863320658#	0.1850134369#	53.3579374348#
H100	10.9279284735	-0.5933675554	52.4811408161
C101	11.0764121980	-0.6100749627	54.6913657935
H102	10.7718389726	-1.6649456900	54.7173672572
C103	10.3566916849	0.1170202268	55.8372224653
H104	9.2672269088	0.0073443137	55.7585948184
H105	10.5859371487	1.1923235538	55.8203148598
H106	10.6629244400	-0.2698266901	56.8144052815
C107	12.5965381329	-0.5303285051	54.8823630045
H108	13.1437715577	-1.0234487372	54.0728416437
H109	12.9072213156	-1.0096896722	55.8158615020
H110	12.9220026505	0.5194266127	54.9161846592
H111	13.4550779161#	-12.2303883822#	53.1883140366#
C112	13.9110016834#	-11.6210556829#	52.4219867721#
H113	14.8020686856	-11.1333552545	52.8252806680
H114	14.1530883957	-12.2127009183	51.5349481435
N115	12.8392152498	-10.6662870217	52.1127009472
H116	12.1329339760	-10.4905339899	52.8382502065
C117	12.7098979376	-9.9241076369	51.0195683095
N118	13.7731821950	-9.7394286871	50.1889726297
H119	13.5724588564	-9.1268157411	49.4051088621
H120	14.6108420579	-9.4107454687	50.6800931831
N121	11.4979155214	-9.4238875194	50.6965064608
H122	10.7903742648	-9.5206631699	51.4322190533
H123	11.4919912725	-8.4675710199	50.3210080479
H124	19.2029281678	-8.2171334505	61.7096764553
C125	19.5978935734#	-7.2298792539#	61.9900504166#
H126	20.6404190026	-7.3649002523	62.2964338953
C127	18.8336714453	-6.6887675662	63.1972861936
O128	19.3850816547	-6.2238608714	64.1917184976
C129	19.4918715346#	-6.2717985100#	60.7990632931#
H130	19.8807682702	-5.2884203067	61.0886364506
H131	18.4378664817	-6.1071689423	60.5525828772
C132	20.2128761179	-6.7617869402	59.5290652821
H133	19.9269405202	-7.7970958098	59.3039241087
H134	21.3001533573	-6.7894571383	59.6917220767
C135	19.9971936051	-5.9945392478	58.2116651309
O136	20.3518784016	-6.4725974853	57.1457154433
O137	19.4284457126	-4.7977809127	58.3551328028
N138	17.4787620245	-6.7533787024	63.0413347181
H139	17.1267139510	-7.0562845160	62.1417538182
C140	16.5368852110#	-6.0058845416#	63.8530407145#
H141	15.8405884500	-6.7019023098	64.3429017672
H142	17.1136789233	-5.5064238679	64.6353606966
C143	15.7391832391	-4.9834275742	63.0049165605
H144	16.3913495978	-4.1505843518	62.7217253356
H145	14.9417719128	-4.5729891889	63.6392544572

C146	15.1576264774	-5.6060212725	61.7463069881
C147	14.0968438011	-6.5247113694	61.8054423141
H148	13.6309885179	-6.7479540495	62.7640245460
C149	15.7301772991	-5.3216763237	60.4939945989
H150	16.5210097940	-4.5784646949	60.4318591737
C151	13.6272797083	-7.1495040009	60.6471144400
H152	12.8015804258	-7.8548697861	60.7133688023
C153	15.2669564280	-5.9516685650	59.3331257587
H154	15.7179397087	-5.7264431859	58.3698080103
C155	14.2164323255	-6.8711393622	59.4081015728
H156	13.8568882608	-7.3523712837	58.5026975350
H157	9.0037559684#	0.7050734990#	60.7752692644#
C158	8.1269805690#	0.1560800723#	60.4649995053#
H159	7.2836720279#	0.4592419539#	61.0677700676#
H160	10.5951455335	-6.3416825139	57.8620405692
H161	18.2592784185#	-9.4987526634#	50.1505401189#
C162	17.5839749691#	-8.6559203885#	50.1520269376#
H163	17.2773627430#	-8.4419890639#	49.1387987622#
H164	23.2928603358	-4.2722690770	52.5603979530
H165	10.7283057213	-5.3802254194	50.7383042172
H166	11.6927149159	-8.1857141114	57.9163663769
H167	13.3251588544	-4.7824378826	57.7490396172
C168	6.7808425756	-8.6161995698	55.9902427435
H169	7.1739179780	-8.9231569664	55.0142542166
H170	6.0035738118	-7.8623821096	55.8090314127
N171	11.6895049373	-7.7583925638	56.9862838970
N172	10.9806777836	-6.6080859570	56.9522525246
H173	7.1493940635#	-5.8354099101#	59.8218942514#
C174	6.9590150151#	-4.8009332991#	59.5000036570#
H175	5.9039259990#	-4.4903550016#	59.5181449689#
H176	7.5000258813	-4.1595222725	60.2092598402
C177	7.4486243428	-4.5018279044	58.0792531703
H178	8.5000911369	-4.7973748730	57.9528452763
H179	7.4022477830	-3.4171948776	57.9151859327
O180	6.6346609797	-5.0992674985	57.0878799953
H181	7.0253092967	-5.9907963740	56.9442797364
H182	14.9489578015	-9.1988049357	56.3920426466

The E_5 structure with N_2H_4 and one water
Energies: E= -8106.818468, solv = -0.200322, disp = --248.46 Z_0 = 917.66

Mo1	16.5130486329	-5.7762007136	53.1812700656
Fe2	9.3532059505	-6.7625733100	55.4246206035
Fe3	11.6745157715	-5.2248870748	55.5038200648
Fe4	11.0048591741	-6.5645445073	53.2254263140
Fe5	11.9890484197	-8.2985056566	55.0105008658
Fe6	14.5965219248	-7.7361667227	54.2644821466
Fe7	14.2517300433	-5.2444975446	54.9994223951
Fe8	13.4095566906	-5.8057899710	52.4952499671

C9	17.8019287977	-2.1901994737	54.1389605797
H10	17.9675423087	-1.1796915869	53.7600105234
H11	16.7196141177	-2.3198639589	54.2617310107
C12	18.3117155586	-3.2467198878	53.1331406330
C13	19.7803861410	-2.9637311475	52.7483643422
H14	20.4011588552	-3.1226700240	53.6345222972
H15	19.8575345534	-1.9099466727	52.4595769653
C16	20.2764665532	-3.8435968757	51.5808270474
H17	19.8541954028	-3.5099311087	50.6319081130
H18	19.9568091924	-4.8768750251	51.7595734549
C19	17.5381114427	-3.3862107778	51.7856635045
O20	17.2170413686	-4.6167946373	51.4885105378
O21	17.4128421046	-2.4069595211	51.0529846350
O22	18.2217862435	-4.5285142406	53.7871446550
C23	12.7072220019	-6.5062444116	54.2962627520
S24	13.3247566788	-3.7518098030	56.5987608910
S25	16.1853547110	-6.4962600591	55.5385033299
S26	9.6395500401	-4.7527444575	54.0990435159
S27	13.9139033601	-9.8532959632	55.2757968810
S28	14.7603717429	-3.9405491714	53.0941612694
S29	9.9489189030	-8.6371098675	53.8471336591
S30	15.0867760511	-7.4511893505	51.9933750024
S31	11.7178165597	-6.2716501557	50.7228001322
H32	13.6351743909#	-2.1902105586#	49.0425560530#
C33	13.2979241567#	-2.0241174180#	50.0550233588#
H34	13.8757240311	-2.6924930256	50.6984125493
H35	13.4973306084	-0.9870306991	50.3487580495
N36	11.8685785124	-2.3395159951	50.1345102627
H37	11.2806958220	-2.0044897122	49.3845746845
C38	11.2208302958	-2.7617333577	51.2366667350
N39	11.8260205967	-2.9786346339	52.3861189638
H40	11.2941878836	-3.4708282037	53.1197877544
H41	12.8470031577	-2.9754727247	52.5065777288
N42	9.8655128379	-2.9205236037	51.1792504812
H43	9.4721974383	-3.1699850284	50.2810402722
H44	9.4650035985	-3.4369689966	51.9734429278
H45	7.9259854547	0.4022716034	59.4163446883
C46	8.3322713300	-1.3554794881	60.6628050602
H47	8.3391589140	-1.5801299641	61.7357860762
H48	7.4758734746	-1.8898967238	60.2326265118
C49	9.6198861619	-1.8646639703	60.0946158170
N50	10.6999823042	-2.1272711446	60.9147468048
C51	9.9639342967	-2.0819270186	58.7788125518
H52	9.4099420196	-1.9988258920	57.8565748906
C53	11.6733488899	-2.4980253347	60.1034380007
H54	12.6719313415	-2.7867244694	60.4011167371
N55	11.2807057055	-2.4844330737	58.8007784228
H56	19.2478159469	-4.3859958345	57.4432925008
H57	16.7312662484	-8.8344317476	50.7994390121
C58	18.4229864967	-7.4388202831	50.5992339760

H59	17.8326387076	-6.5209937852	50.5455279977
H60	19.2543870232	-7.3203025767	49.8900147735
C61	18.9923937638	-7.5690863826	51.9761403743
N62	18.2707013765	-7.2102638428	53.1044467873
C63	20.2193888071	-8.0373316574	52.3783715211
H64	21.0626940658	-8.4102424720	51.8177561803
C65	19.0371645937	-7.4517937472	54.1564070190
H66	18.7904005367	-7.2367235183	55.1847289622
N67	20.2224260925	-7.9663050992	53.7620484069
H68	20.9946805597	-8.1451425987	54.3867201112
H69	6.9363729123	-10.5734028411	56.9433537218
C70	6.1750544637#	-9.8249629141#	56.6900007368#
H71	5.5309554532#	-10.2322283189#	56.0424861352#
H72	5.6932325284#	-9.5371413206#	57.6127205306#
S73	8.0942733513	-7.8718400119	57.0884439560
H74	11.8554086246	-2.7286541752	57.9909785275
H75	18.4176976167	-4.3256373093	54.7887085667
C76	18.4284837982	-2.3013547384	55.5605079715
O77	18.7428076573	-3.4918470176	55.9634647640
O78	18.5474031842	-1.2594396262	56.2295443127
C79	21.7692017670	-3.8470727224	51.4700668583
O80	22.4584800655	-3.5452685862	50.5151804167
O81	22.3208169703#	-4.2658019664#	52.6398596085#
H82	15.7024655314#	-0.1996319838#	61.4912314292#
C83	14.8549938681#	-0.0829907253#	60.8320019100#
H84	13.9165015893#	-0.2867338795#	61.3260937710#
H85	14.8248295655	0.9913701636	60.5824757810
C86	15.0599853431#	-0.8751945719#	59.5441990159#
H87	14.3368026981	-0.5477482867	58.7868035286
H88	14.8613834824	-1.9381940344	59.7237858254
C89	16.4798028592	-0.7504957210	58.9772742881
H90	16.8336341865	0.2892706228	59.0488485332
H91	16.5036292013	-0.9930078455	57.9089406021
C92	17.4952361708	-1.6523332741	59.6924749477
O93	17.2637240155	-2.1839406412	60.7853758607
N94	18.6759607164	-1.8004174400	59.0375169770
H95	19.2602000149	-2.5561937633	59.3750486985
H96	18.7310730642	-1.5928319661	58.0283579770
C97	10.6519927198#	0.0100028950#	53.3460013173#
H98	11.1636988504#	0.9592721312#	53.2872698160#
H99	9.5863320703#	0.1850134616#	53.3579374512#
H100	10.9254608084	-0.5918717841	52.4810830835
C101	11.1076397691	-0.5940653629	54.6883977977
H102	10.7934508122	-1.6443587459	54.7482598211
C103	10.4376330354	0.1699930224	55.8402272599
H104	9.3448266458	0.0709931000	55.8037345503
H105	10.6769096147	1.2419151032	55.7861799745
H106	10.7756091234	-0.1934973907	56.8158380249
C107	12.6356878517	-0.5232716002	54.8198504529
H108	13.1459261458	-1.0637530963	54.0157198093

H109	12.9788426510	-0.9570880983	55.7637190773
H110	12.9725455918	0.5231014743	54.7860109981
H111	13.4550779886#	-12.2303883529#	53.1883141030#
C112	13.9110015070#	-11.6210556544#	52.4219866800#
H113	14.8183558873	-11.1434077441	52.7974478110
H114	14.1046527234	-12.1902479027	51.5099438341
N115	12.8205651673	-10.6544958110	52.1915690476
H116	12.1929200007	-10.4938724584	52.9785580960
C117	12.5633398463	-9.9337292600	51.1057855741
N118	13.5364967746	-9.7607231941	50.1674569937
H119	13.2580706893	-9.1485821063	49.4071621389
H120	14.4135684102	-9.4279793931	50.5826604641
N121	11.3244436691	-9.4528152648	50.9039819187
H122	10.6921077284	-9.4858356697	51.7162698145
H123	11.2713334739	-8.5310578321	50.4583113159
H124	19.2103626542	-8.2190987230	61.7063284853
C125	19.5978935294#	-7.2298792423#	61.9900504514#
H126	20.6399237156	-7.3590500082	62.3006984210
C127	18.8278946565	-6.6985012178	63.1969014731
O128	19.3750777885	-6.2613302870	64.2065473461
C129	19.4918714467#	-6.2717985673#	60.7990632593#
H130	19.8550334474	-5.2799818245	61.0929976159
H131	18.4390849311	-6.1299395752	60.5337998415
C132	20.2452706694	-6.7502224289	59.5424213601
H133	20.0145634034	-7.8027723927	59.3365091710
H134	21.3312878441	-6.7175477307	59.7139317733
C135	19.9967310681	-6.0211577214	58.2093657759
O136	20.3240238198	-6.5282871048	57.1476836379
O137	19.4373455033	-4.8181958983	58.3337774501
N138	17.4745429405	-6.7413295031	63.0264718563
H139	17.1243866138	-7.0196523388	62.1182608167
C140	16.5368852292#	-6.0058844857#	63.8530407178#
H141	15.8243385881	-6.7071101073	64.3106673702
H142	17.1145668907	-5.5456658084	64.6584672927
C143	15.7609634676	-4.9404655537	63.0401724715
H144	16.4321760408	-4.1154274966	62.7785651037
H145	14.9746687220	-4.5292595576	63.6879560653
C146	15.1632034167	-5.5170113469	61.7696573219
C147	14.0834163826	-6.4147411593	61.8109414454
H148	13.6141860366	-6.6479514112	62.7656962904
C149	15.7406813106	-5.2176193347	60.5243637988
H150	16.5465649596	-4.4896409590	60.4761620974
C151	13.6012735974	-7.0072122701	60.6413381485
H152	12.7609483018	-7.6956601981	60.6868092677
C153	15.2677651155	-5.8187360243	59.3523428150
H154	15.7294558876	-5.5915633595	58.3950070679
C155	14.2000390750	-6.7193452323	59.4094181341
H156	13.8294148464	-7.1795553928	58.4993536884
H157	9.0037559587#	0.7050735042#	60.7752692824#
C158	8.1269806329#	0.1560799722#	60.4649994229#

H159	7.2836720304#	0.4592419331#	61.0677700818#
H160	10.0459075383	-4.8512889937	57.4129410331
H161	18.2592785494#	-9.4987525584#	50.1505400596#
C162	17.5839746725#	-8.6559205372#	50.1520273012#
H163	17.2773628729#	-8.4419889799#	49.1387987407#
H164	23.2858270480	-4.2342258383	52.5017446440
H165	10.8151753957	-5.2764753361	50.6026706053
H166	11.3262426967	-5.8048089290	58.8150930849
H167	13.4653109348	-4.5553019208	57.6791562569
C168	6.8290193069	-8.6371679763	56.0074705990
H169	7.3063944595	-8.9573324286	55.0756404825
H170	6.0704430188	-7.8865885673	55.7501978825
N171	11.2075590369	-6.3671560482	57.9652352687
N172	10.4755331900	-5.7050439358	57.0485356234
H173	7.1493940348#	-5.8354098945#	59.8218943186#
C174	6.9590153890#	-4.8009335781#	59.5000035441#
H175	5.9039260034#	-4.4903549838#	59.5181449197#
H176	7.5128796400	-4.1526527142	60.1916635692
C177	7.3597331989	-4.4783114450	58.0616108497
H178	8.4071914644	-4.7228294302	57.8835900482
H179	7.2491958804	-3.3980618517	57.9033398034
O180	6.5291625357	-5.1123449512	57.1060567445
H181	6.8834391528	-6.0270154188	57.0364120724
H182	13.9564641517	-9.5993787464	56.6010492873
O183	11.3532421852	-8.8143466017	57.0176859396
H184	11.3773049989	-7.9255887254	57.5218844594
H185	10.3934778545	-9.0099454708	56.9718140024

The TS structure in E_5 in **Figure 2**

Energies: E= -8106.821140, solv = -0.201958, disp = --244.77, Z_0 = 915.17

Mo1	16.7646460000	-5.4813270000	53.2458810000
Fe2	9.6441040000	-7.0578350000	55.5349060000
Fe3	11.9203160000	-5.4156930000	55.6633440000
Fe4	11.4433250000	-6.9693340000	53.4500050000
Fe5	12.6349830000	-8.3892640000	55.4616320000
Fe6	15.0122660000	-7.6442290000	54.1269100000
Fe7	14.5669950000	-5.3310040000	55.2920350000
Fe8	13.6081860000	-5.6293900000	52.7807710000
C9	17.9708600000	-1.9004030000	54.4572450000
H10	18.1123980000	-0.8683940000	54.1306540000
H11	16.8937410000	-2.0554400000	54.5996790000
C12	18.4639450000	-2.8881610000	53.3791040000
C13	19.9232400000	-2.5764350000	52.9747190000
H14	20.5655940000	-2.8209910000	53.8262950000
H15	20.0070180000	-1.5023830000	52.7789020000
C16	20.3710450000	-3.3546210000	51.7146770000
H17	20.0037610000	-2.8646810000	50.8115010000
H18	19.9531390000	-4.3656540000	51.7585660000

C19	17.6427130000	-2.9337250000	52.0572990000
O20	17.3464710000	-4.1401250000	51.6565060000
O21	17.4498700000	-1.8938200000	51.4315190000
O22	18.4046580000	-4.2102000000	53.9495790000
C23	13.1023220000	-6.6173540000	54.5190790000
S24	13.4362480000	-3.9639870000	56.9272150000
S25	16.5803030000	-6.4739330000	55.5350390000
S26	9.9601100000	-5.0941100000	54.1785330000
S27	14.6410840000	-9.8308970000	55.2132380000
S28	14.8872010000	-3.7733700000	53.4758940000
S29	10.5342670000	-9.0096050000	54.2065310000
S30	15.3352290000	-7.0540370000	51.9035140000
S31	11.9427200000	-6.2345780000	51.0096140000
H32	13.6351740000	-2.1902110000	49.0425560000
C33	13.2979240000	-2.0241170000	50.0550230000
H34	13.9325770000	-2.6328360000	50.7052570000
H35	13.4338920000	-0.9689520000	50.3194870000
N36	11.8958400000	-2.4304890000	50.1789460000
H37	11.2797710000	-2.2120890000	49.4096670000
C38	11.2993770000	-2.8642690000	51.3043830000
N39	11.9364210000	-2.9862000000	52.4534810000
H40	11.4567110000	-3.4959990000	53.2080180000
H41	12.9543170000	-2.8995620000	52.5616150000
N42	9.9596070000	-3.1250980000	51.2808910000
H43	9.5731760000	-3.4338280000	50.3981510000
H44	9.6167080000	-3.6562060000	52.0909880000
H45	7.9294160000	0.3965000000	59.4144240000
C46	8.3397460000	-1.3564750000	60.6773790000
H47	8.2281080000	-1.5825620000	61.7444960000
H48	7.5490550000	-1.9060210000	60.1527020000
C49	9.6921950000	-1.8471720000	60.2629010000
N50	10.7101810000	-1.9716320000	61.1887010000
C51	10.1523630000	-2.1855010000	59.0094060000
H52	9.6721900000	-2.2296720000	58.0438290000
C53	11.7611950000	-2.3782480000	60.5004890000
H54	12.7413070000	-2.5905870000	60.9047880000
N55	11.4769240000	-2.5204870000	59.1774390000
H56	19.4151270000	-4.2335850000	57.5655420000
H57	16.7407500000	-8.8215170000	50.8138860000
C58	18.4165910000	-7.4125060000	50.5511530000
H59	17.7883650000	-6.5188370000	50.5227600000
H60	19.1923960000	-7.2774040000	49.7837390000
C61	19.0849660000	-7.4852900000	51.8876410000
N62	18.5614250000	-6.8700540000	53.0136900000
C63	20.2621030000	-8.1022110000	52.2378680000
H64	20.9715910000	-8.6753520000	51.6605180000
C65	19.3974920000	-7.1015900000	54.0155170000
H66	19.2982420000	-6.7500220000	55.0321260000
N67	20.4373690000	-7.8513680000	53.5876110000
H68	21.2136640000	-8.1302520000	54.1682030000

H69	6.9276970000	-10.5807740000	56.9493840000
C70	6.1750540000	-9.8249630000	56.6900010000
H71	5.5309550000	-10.2322280000	56.0424860000
H72	5.6932330000	-9.5371410000	57.6127210000
S73	8.1260950000	-7.8964340000	57.1135200000
H74	12.1150170000	-2.8150910000	58.4366150000
H75	18.6045580000	-4.0699990000	54.9620820000
C76	18.6387300000	-2.0884950000	55.8477870000
O77	18.9441610000	-3.3046670000	56.1743280000
O78	18.7964110000	-1.0906470000	56.5705650000
C79	21.8604670000	-3.4754760000	51.6332490000
O80	22.6165990000	-2.9726330000	50.8255730000
O81	22.3208170000	-4.2658020000	52.6398600000
H82	15.7024660000	-0.1996320000	61.4912310000
C83	14.8549940000	-0.0829910000	60.8320020000
H84	13.9165020000	-0.2867340000	61.3260940000
H85	14.8298700000	0.9924840000	60.5885800000
C86	15.0599850000	-0.8751950000	59.5441990000
H87	14.3953070000	-0.4894930000	58.7611750000
H88	14.7797000000	-1.9223300000	59.7020550000
C89	16.5034870000	-0.8589810000	59.0404270000
H90	16.9385450000	0.1487730000	59.1061340000
H91	16.5527190000	-1.1234520000	57.9777710000
C92	17.4124200000	-1.8345070000	59.7997720000
O93	17.0140010000	-2.5128390000	60.7521560000
N94	18.6947640000	-1.8741270000	59.3451930000
H95	19.2223090000	-2.6862510000	59.6440020000
H96	18.8781360000	-1.5550790000	58.3830480000
C97	10.6519930000	0.0100030000	53.3460010000
H98	11.1636990000	0.9592720000	53.2872700000
H99	9.5863320000	0.1850130000	53.3579370000
H100	10.9257080000	-0.5896480000	52.4783510000
C101	11.0742830000	-0.6260260000	54.6870620000
H102	10.7583500000	-1.6780550000	54.7019160000
C103	10.3617250000	0.0923290000	55.8446020000
H104	9.2714870000	-0.0153500000	55.7730330000
H105	10.5925000000	1.1673060000	55.8375280000
H106	10.6759600000	-0.3041030000	56.8157310000
C107	12.5962110000	-0.5639260000	54.8811300000
H108	13.1430410000	-1.0326770000	54.0565150000
H109	12.9039790000	-1.0749250000	55.7996200000
H110	12.9298650000	0.4816120000	54.9488500000
H111	13.4550780000	-12.2303880000	53.1883140000
C112	13.9110020000	-11.6210560000	52.4219870000
H113	14.8404060000	-11.1847800000	52.7942130000
H114	14.0789850000	-12.1941370000	51.5058760000
N115	12.8563240000	-10.6217090000	52.2035710000
H116	12.1917780000	-10.4597550000	52.9694940000
C117	12.6619910000	-9.8626360000	51.1339040000
N118	13.6761910000	-9.6426930000	50.2545710000

H119	13.4434150000	-8.9872520000	49.5157150000
H120	14.5520440000	-9.3725710000	50.7058240000
N121	11.4310340000	-9.3757180000	50.8835090000
H122	10.7756100000	-9.4866250000	51.6627020000
H123	11.4221050000	-8.4045260000	50.5452340000
H124	19.2155120000	-8.2217670000	61.7094470000
C125	19.5978940000	-7.2298790000	61.9900500000
H126	20.6414040000	-7.3517390000	62.2995850000
C127	18.8321900000	-6.6878790000	63.1934210000
O128	19.3857940000	-6.2187630000	64.1848360000
C129	19.4918710000	-6.2717990000	60.7990630000
H130	19.7916060000	-5.2661720000	61.1156200000
H131	18.4446950000	-6.1814510000	60.4905610000
C132	20.3258080000	-6.6969900000	59.5783580000
H133	20.1568510000	-7.7532450000	59.3385580000
H134	21.3986070000	-6.6089030000	59.8073960000
C135	20.0903590000	-5.9350320000	58.2660150000
O136	20.3656210000	-6.4326870000	57.1862920000
O137	19.6071950000	-4.7064070000	58.4374630000
N138	17.4798720000	-6.7422890000	63.0339140000
H139	17.1227370000	-7.0735990000	62.1466440000
C140	16.5368850000	-6.0058840000	63.8530410000
H141	15.9241980000	-6.7074410000	64.4380680000
H142	17.1206380000	-5.4081090000	64.5579770000
C143	15.6141350000	-5.1098570000	62.9902930000
H144	16.1880320000	-4.2695910000	62.5846930000
H145	14.8392090000	-4.6950810000	63.6490030000
C146	14.9895190000	-5.8937660000	61.8516750000
C147	14.0005990000	-6.8603200000	62.0932040000
H148	13.6039360000	-6.9810880000	63.1003570000
C149	15.4736060000	-5.7435800000	60.5416670000
H150	16.2068020000	-4.9664790000	60.3357780000
C151	13.5284780000	-7.6738110000	61.0618520000
H152	12.7616760000	-8.4179270000	61.2652270000
C153	15.0087990000	-6.5648370000	59.5074860000
H154	15.4015530000	-6.4412310000	58.5006430000
C155	14.0417230000	-7.5405500000	59.7673510000
H156	13.6757360000	-8.1801780000	58.9700600000
H157	9.0037560000	0.7050740000	60.7752690000
C158	8.1269810000	0.1560800000	60.4649990000
H159	7.2836720000	0.4592420000	61.0677700000
H160	10.1831480000	-5.1338420000	57.5564390000
H161	18.2592790000	-9.4987530000	50.1505400000
C162	17.5839750000	-8.6559210000	50.1520270000
H163	17.2773630000	-8.4419890000	49.1387990000
H164	23.2914220000	-4.2731800000	52.5451700000
H165	10.8032900000	-5.5191350000	51.1123780000
H166	11.3544890000	-6.1626520000	59.0638530000
H167	13.5097450000	-4.8198560000	57.9748290000
C168	6.8573700000	-8.6433630000	56.0174190000

H169	7.3394830000	-8.9766150000	55.0915850000
H170	6.1142670000	-7.8815720000	55.7480070000
N171	11.2803800000	-6.6529350000	58.1719370000
N172	10.6502470000	-5.9711460000	57.2042320000
H173	7.1493940000	-5.8354100000	59.8218940000
C174	6.9590150000	-4.8009340000	59.5000040000
H175	5.9039260000	-4.4903550000	59.5181450000
H176	7.4986670000	-4.1587930000	60.2103400000
C177	7.4168200000	-4.4754560000	58.0779700000
H178	8.4711660000	-4.7379160000	57.9419360000
H179	7.3352140000	-3.3910090000	57.9247980000
O180	6.6181670000	-5.0880250000	57.0858980000
H181	6.9874530000	-5.9957830000	56.9929070000
H182	15.2384060000	-9.4780590000	56.3729370000
O183	11.9350900000	-8.8778410000	57.3258830000
H184	11.6375290000	-7.8585300000	57.8251110000
H185	11.1034150000	-9.3682120000	57.2001590000

The N_2H_3 product structure in E_5 with one water
Energies: E= -8106.860552, solv = -0.200526, disp = --249.85, Z_0 = 921.03

Mo1	16.4948931686	-5.8358548457	53.2112477305
Fe2	9.4427180961	-6.6645076412	55.8195366842
Fe3	11.8388298225	-5.2738633271	55.7982264848
Fe4	10.8729391845	-6.4078546348	53.5707688466
Fe5	11.9499634899	-8.3391740453	55.0841916559
Fe6	14.4433544679	-7.7286204961	54.3609561081
Fe7	14.3535709621	-5.1868908979	54.9888935226
Fe8	13.2940106307	-5.9517801610	52.5186117027
C9	17.7869662518	-2.2174564476	54.1502920195
H10	17.9594112691	-1.2122141917	53.7608312741
H11	16.7035072274	-2.3389410630	54.2724080900
C12	18.2947579469	-3.2892254745	53.1591396148
C13	19.7656110713	-3.0146043902	52.7719074774
H14	20.3863607024	-3.1801598397	53.6567336419
H15	19.8491785308	-1.9598098127	52.4881875417
C16	20.2565045794	-3.8902726180	51.5995085932
H17	19.8219601669	-3.5607875270	50.6546083714
H18	19.9470264272	-4.9265874452	51.7793817100
C19	17.5218814842	-3.4421325847	51.8153561160
O20	17.2107098663	-4.6798188486	51.5276612045
O21	17.3804119899	-2.4715328147	51.0762168964
O22	18.1946581522	-4.5613515413	53.8269044472
C23	12.7326918394	-6.5225866697	54.4014936615
S24	13.5577155396	-3.8101938083	56.7910224250
S25	16.1675901792	-6.5944455968	55.5202579369
S26	9.7773486581	-4.5515592511	54.3813849256
S27	13.7891783177	-9.7215369495	55.2794658233
S28	14.7357096845	-4.0418242282	53.0730901959

S29	9.8095624103	-8.4116094778	53.9569393389
S30	15.0669038555	-7.5097795496	52.0971309152
S31	11.9640509773	-5.9691206744	50.4440553206
H32	13.6351744575#	-2.1902105946#	49.0425560811#
C33	13.2979240424#	-2.0241173959#	50.0550233341#
H34	13.8910391868	-2.6856715353	50.6913900285
H35	13.4902309597	-0.9834026916	50.3400483199
N36	11.8748602516	-2.3465408175	50.1553147279
H37	11.3058330464	-2.2440802742	49.3288587956
C38	11.2512501754	-2.7904982915	51.2570775431
N39	11.8624804725	-2.8973218277	52.4219040048
H40	11.3628978692	-3.3844862971	53.1809209464
H41	12.8802017811	-2.8821865044	52.5058199030
N42	9.9076662348	-3.0319949844	51.2030346924
H43	9.5640581496	-3.4128438304	50.3302281477
H44	9.5380269743	-3.5123773879	52.0313520890
H45	7.9281527583	0.4039692792	59.4162133692
C46	8.3370331797	-1.3561340534	60.6596609070
H47	8.2874847309	-1.5865668467	61.7305955743
H48	7.5107964465	-1.8961050815	60.1813830131
C49	9.6572632962	-1.8567879770	60.1614434544
N50	10.7240653327	-1.9989296107	61.0283003650
C51	10.0410909101	-2.1946183968	58.8825863532
H52	9.5085273466	-2.2281335026	57.9445532834
C53	11.7263765241	-2.4214479788	60.2813574667
H54	12.7229961810	-2.6531484719	60.6301462509
N55	11.3689079287	-2.5485236289	58.9755135511
H56	19.2297318729	-4.3981062593	57.4467424509
H57	16.7304316603	-8.8309579032	50.8018882917
C58	18.4242468877	-7.4387613075	50.5949179814
H59	17.8347745380	-6.5205908774	50.5430926865
H60	19.2533039278	-7.3209554876	49.8831034153
C61	18.9990846788	-7.5706690168	51.9688819179
N62	18.2803474233	-7.2258382258	53.1035517474
C63	20.2320895287	-8.0304441631	52.3616880874
H64	21.0752690627	-8.3919061554	51.7936492675
C65	19.0556987812	-7.4673275208	54.1499437255
H66	18.8183358196	-7.2612820663	55.1819897419
N67	20.2426975568	-7.9670396925	53.7451480716
H68	21.0177205088	-8.1513226455	54.3648272283
H69	6.9196449720	-10.5888876564	56.9508075468
C70	6.1750545782#	-9.8249628556#	56.6900006930#
H71	5.5309554436#	-10.2322283382#	56.0424861570#
H72	5.6932325222#	-9.5371413005#	57.6127205212#
S73	8.0892345767	-7.9539497976	57.2717720397
H74	11.9604431006	-2.8842268399	58.2158022022
H75	18.3922057162	-4.3449236759	54.8273039547
C76	18.4081535006	-2.3169594672	55.5756935764
O77	18.7155344580	-3.5074292644	55.9878114460
O78	18.5288828115	-1.2726085510	56.2377634542

C79	21.7481496762	-3.8869130195	51.4672233081
O80	22.4196859687	-3.6143131223	50.4915035876
O81	22.3208169154#	-4.2658019603#	52.6398596067#
H82	15.7024655320#	-0.1996319825#	61.4912314285#
C83	14.8549938909#	-0.0829907195#	60.8320019102#
H84	13.9165015894#	-0.2867338794#	61.3260937710#
H85	14.8266435848	0.9914913128	60.5839944842
C86	15.0599853240#	-0.8751945838#	59.5441990048#
H87	14.3508115352	-0.5314604813	58.7811059848
H88	14.8380341317	-1.9342789860	59.7180903179
C89	16.4862443862	-0.7826727887	58.9918657189
H90	16.8772636193	0.2411150535	59.0916637245
H91	16.5109169894	-0.9993824474	57.9178395818
C92	17.4617861261	-1.7408028016	59.6876928266
O93	17.1853408357	-2.3294705919	60.7391967954
N94	18.6627051246	-1.8703298368	59.0620340894
H95	19.2165358758	-2.6617915277	59.3685955599
H96	18.7330875999	-1.6291329851	58.0627646661
C97	10.6519926485#	0.0100027786#	53.3460013420#
H98	11.1636988803#	0.9592721209#	53.2872699108#
H99	9.5863320776#	0.1850135109#	53.3579373831#
H100	10.9264678931	-0.5926901370	52.4828398138
C101	11.0714384009	-0.6101332380	54.6947465068
H102	10.7448030427	-1.6572044750	54.7295080353
C103	10.3693879070	0.1347080639	55.8406348049
H104	9.2781757377	0.0443684404	55.7638858920
H105	10.6182022911	1.2057261626	55.8213788682
H106	10.6691527475	-0.2569806588	56.8184648038
C107	12.5938923690	-0.5582446073	54.8838722051
H108	13.1294953087	-1.0618521385	54.0724215122
H109	12.8988004157	-1.0379989812	55.8197436524
H110	12.9414154942	0.4846813751	54.9134629425
H111	13.4550780037#	-12.2303883805#	53.1883140901#
C112	13.9110014648#	-11.6210555987#	52.4219867088#
H113	14.8671983404	-11.2270262062	52.7767656260
H114	14.0249707485	-12.1801129835	51.4894991573
N115	12.9090101981	-10.5700921108	52.2573427682
H116	12.5570967117	-10.2116071506	53.1530533621
C117	12.6775015049	-9.8228383740	51.1878746450
N118	13.5428910318	-9.8545200809	50.1272177527
H119	13.3038387247	-9.1874129368	49.4006929157
H120	14.5129506035	-9.7444393758	50.4146586907
N121	11.5479204934	-9.1098210848	51.0951408226
H122	10.9324199841	-9.1053149433	51.9172972314
H123	11.6298960622	-8.1726640761	50.6646497511
H124	19.1870186589	-8.2123657464	61.7176271835
C125	19.5978936888#	-7.2298791318#	61.9900505892#
H126	20.6414640396	-7.3748181113	62.2885965643
C127	18.8478352503	-6.6564550278	63.1932287395
O128	19.4088676741	-6.1016007869	64.1344634464

C129	19.4918713166#	-6.2717986569#	60.7990631995#
H130	19.8885951496	-5.2904144019	61.0836395015
H131	18.4372534178	-6.1069783155	60.5578074678
C132	20.2075547617	-6.7796762480	59.5325281568
H133	19.9292845038	-7.8216132328	59.3300173522
H134	21.2962557811	-6.7945111206	59.6880592985
C135	19.9725772507	-6.0405940268	58.2045580233
O136	20.3039530840	-6.5395250313	57.1398875993
O137	19.4127200383	-4.8384509730	58.3344477477
N138	17.4923871590	-6.7886653706	63.0868927687
H139	17.1331257290	-7.1705522883	62.2208372098
C140	16.5368852094#	-6.0058845833#	63.8530406332#
H141	15.8706433639	-6.6838904428	64.4050212354
H142	17.1071251034	-5.4295459184	64.5855721662
C143	15.6932236227	-5.0764243249	62.9402234880
H144	16.3001762350	-4.2213635435	62.6242570879
H145	14.8598114296	-4.6823724868	63.5374744107
C146	15.1787481761	-5.7994228555	61.7066648776
C147	14.2119219443	-6.8151327609	61.7972196074
H148	13.7475307180	-7.0318513025	62.7578207530
C149	15.7428885536	-5.5248982466	60.4494738893
H150	16.4629596785	-4.7148012752	60.3592303928
C151	13.8370642247	-7.5517660683	60.6704992032
H152	13.0813887808	-8.3278188664	60.7594902238
C153	15.3784021831	-6.2694273167	59.3212837194
H154	15.8430623673	-6.0656552466	58.3600487689
C155	14.4294244171	-7.2918353871	59.4288826524
H156	14.1635735652	-7.8793668984	58.5535230317
H157	9.0037559792#	0.7050734707#	60.7752692839#
C158	8.1269805381#	0.1560798565#	60.4649993419#
H159	7.2836720709#	0.4592419692#	61.0677701202#
H160	10.1167494635	-5.0569345299	57.6878901381
H161	18.2592785905#	-9.4987525253#	50.1505400523#
C162	17.5839745543#	-8.6559205700#	50.1520273975#
H163	17.2773629280#	-8.4419889606#	49.1387987279#
H164	23.2832929929	-4.2376453803	52.4841145798
H165	10.7101403367	-5.7655446787	50.9213309537
H166	12.1293567625	-6.2230777563	58.5955664297
H167	13.9045600884	-4.6714172462	57.7769583274
C168	6.8729898223	-8.6298885248	56.0699712905
H169	7.3931642393	-8.9104957730	55.1503759885
H170	6.1438233592	-7.8505007084	55.8151909938
N171	11.1869660386	-6.5678204647	58.4041407320
N172	10.6438063964	-5.8272786733	57.2799843512
H173	7.1493940425#	-5.8354098864#	59.8218943402#
C174	6.9590154876#	-4.8009334129#	59.5000034201#
H175	5.9039260041#	-4.4903549843#	59.5181449678#
H176	7.5141555291	-4.1535625366	60.1906410702
C177	7.3740691392	-4.4914789110	58.0634998902
H178	8.4273265380	-4.7267499415	57.8988280812

H179	7.2508890768	-3.4173506574	57.8790884324
O180	6.5575143462	-5.1512067040	57.1074926175
H181	6.8653813215	-6.0814776129	57.1209207952
H182	11.3017649825	-10.1486547048	56.8773399612
O183	11.0531236087	-9.2135504937	56.9666553857
H184	11.2963451835	-7.5346627059	58.1009935146
H185	10.0634048331	-9.1398158400	56.9777239392

The N_2H_3 product structure in E_5 without water in **Figure 3**
Energies: E= -8030.437801, solv = -0.207113, disp = --239.23, Z_0 = 905.38

Mo1	16.5883252696	-5.7167419343	53.2159071171
Fe2	9.3786160506	-6.9231488431	55.3922804842
Fe3	11.7543031284	-5.4940915058	55.6111457348
Fe4	11.1663098259	-6.7880180005	53.2122967696
Fe5	12.2410912749	-8.4428129610	55.1224556724
Fe6	14.6475719795	-7.6750794082	54.2959835839
Fe7	14.3725072555	-5.1569729602	55.0738643400
Fe8	13.5008995593	-5.7942176515	52.5611760319
C9	17.8655430080	-2.1211457692	54.2103802828
H10	18.0344456022	-1.1043928364	53.8501211202
H11	16.7830364081	-2.2470226339	54.3339469884
C12	18.3680259085	-3.1619320870	53.1832612506
C13	19.8288051379	-2.8627107830	52.7797072926
H14	20.4629685652	-3.0413076707	53.6526045777
H15	19.8995601656	-1.8020443301	52.5159218648
C16	20.3083622821	-3.7128877575	51.5827043780
H17	19.9073749815	-3.3286021046	50.6436215279
H18	19.9510642488	-4.7406046634	51.7165247495
C19	17.5708750110	-3.2897741986	51.8492634057
O20	17.2586567462	-4.5208614556	51.5400398808
O21	17.4109682132	-2.3012326463	51.1365241624
O22	18.2974679838	-4.4523250036	53.8173230741
C23	12.9030203537	-6.5711022094	54.2931132525
S24	13.3085760703	-4.0619738879	56.9147420496
S25	16.2903948443	-6.4778203255	55.5185772071
S26	9.8381049308	-4.8968421371	54.1392358036
S27	14.1397815716	-9.6912680632	55.2583653018
S28	14.8236753404	-3.8848703348	53.2136254430
S29	10.1339573063	-8.8061737456	53.9076202082
S30	15.2096743915	-7.3654992770	52.0157773986
S31	11.8292648158	-6.2475731746	50.7806554508
H32	13.6351742281#	-2.1902104606#	49.0425559827#
C33	13.2979243752#	-2.0241175392#	50.0550234853#
H34	13.8857356068	-2.6881542162	50.6932417225
H35	13.4944937619	-0.9862542281	50.3475902828

N36	11.8724132645	-2.3508000941	50.1524905411
H37	11.2670043142	-1.9992190694	49.4242444963
C38	11.2507401883	-2.8063567640	51.2580815948
N39	11.8790709162	-3.0320866435	52.3928725415
H40	11.3791587936	-3.5692087725	53.1220596738
H41	12.8964565609	-2.9684775709	52.5011330736
N42	9.8998829177	-2.9991905199	51.2195962561
H43	9.4988916843	-3.2571081716	50.3271698613
H44	9.5243702976	-3.5254092023	52.0192791532
H45	7.9311923201	0.3906850156	59.4131852495
C46	8.3382120266	-1.3540835738	60.6869370098
H47	8.2325982187	-1.5697959631	61.7571524195
H48	7.5428943267	-1.9067585491	60.1727883355
C49	9.6855627590	-1.8518310959	60.2672319823
N50	10.7196717231	-1.9357310612	61.1814775372
C51	10.1251450208	-2.2400533313	59.0218312116
H52	9.6298949574	-2.3303970512	58.0677084100
C53	11.7558113757	-2.3787769232	60.4937529369
H54	12.7412831015	-2.5811224818	60.8902094199
N55	11.4511472739	-2.5688992700	59.1825048419
H56	19.2863258958	-4.3775313787	57.4525694027
H57	16.7354699948	-8.8360516526	50.8035452827
C58	18.4123192173	-7.4241443789	50.5819679332
H59	17.8031352213	-6.5180569861	50.5391510174
H60	19.2226291126	-7.2924186383	49.8509479832
C61	19.0202984300	-7.5359967238	51.9432463523
N62	18.3558573082	-7.1154884278	53.0847029459
C63	20.2391206123	-8.0470587879	52.3164451977
H64	21.0439189344	-8.4740243925	51.7382169606
C65	19.1466521803	-7.3642605999	54.1177057122
H66	18.9502854322	-7.1208604211	55.1507119276
N67	20.2932902540	-7.9395023878	53.6958953456
H68	21.0697673389	-8.1613772129	54.3008184401
H69	6.9627563156	-10.5485430541	56.9282882083
C70	6.1750546855#	-9.8249632704#	56.6900008794#
H71	5.5309553995#	-10.2322281183#	56.0424860625#
H72	5.6932324892#	-9.5371412255#	57.6127204806#
S73	8.0048575209	-7.8027546015	57.0690904067
H74	12.0645519215	-2.9307705338	58.4529635520
H75	18.4978153344	-4.2610585443	54.8215662162
C76	18.4903634200	-2.2593539811	55.6306811760
O77	18.8101289583	-3.4578938387	56.0071624414
O78	18.5991937900	-1.2345830363	56.3254028290
C79	21.8012329581	-3.7670576451	51.4869226693
O80	22.5129505560	-3.4446837018	50.5556409456
O81	22.3208170196#	-4.2658019652#	52.6398595917#
H82	15.7024655256#	-0.1996319939#	61.4912314347#
C83	14.8549938302#	-0.0829907164#	60.8320018982#
H84	13.9165015893#	-0.2867338795#	61.3260937710#
H85	14.8279392593	0.9917102056	60.5854208676

C86	15.0599853807#	-0.8751945640#	59.5441990407#
H87	14.3701101977	-0.5112356436	58.7727663626
H88	14.8108001875	-1.9295056647	59.7089483811
C89	16.4955844213	-0.8131734655	59.0141226677
H90	16.9100762860	0.2000717299	59.1247365382
H91	16.5324503043	-1.0239340634	57.9392532034
C92	17.4382558478	-1.7996524905	59.7161373278
O93	17.1206659753	-2.4146733133	60.7409772305
N94	18.6570285123	-1.9239486994	59.1265127708
H95	19.1924815245	-2.7321260289	59.4219585787
H96	18.7611454813	-1.6500966406	58.1385278378
C97	10.6519927042#	0.0100028542#	53.3460013208#
H98	11.1636988619#	0.9592721258#	53.2872698297#
H99	9.5863320713#	0.1850134695#	53.3579374500#
H100	10.9189917794	-0.5794852580	52.4684396505
C101	11.0721509212	-0.6552205104	54.6725686683
H102	10.7314285119	-1.6989015179	54.6702528722
C103	10.3868755216	0.0540283783	55.8507319877
H104	9.2940045328	-0.0146794650	55.7758243481
H105	10.6550891672	1.1201373468	55.8753547879
H106	10.6836447153	-0.3841374586	56.8097633498
C107	12.5965679608	-0.6373508004	54.8509929978
H108	13.1182851915	-1.1124235745	54.0139730757
H109	12.8980149746	-1.1677785350	55.7606820228
H110	12.9627402941	0.3967053971	54.9271934800
H111	13.4550779727#	-12.2303882919#	53.1883141421#
C112	13.9110015598#	-11.6210557510#	52.4219866149#
H113	14.7874268856	-11.1143882823	52.8309101484
H114	14.1428420961	-12.2019492734	51.5252202756
N115	12.8142062418	-10.6820512039	52.1385018480
H116	12.1940614942	-10.4728715717	52.9240823186
C117	12.6244482676	-9.9457246559	51.0545602542
N118	13.6391673640	-9.7736338211	50.1607309672
H119	13.4146753869	-9.1359219839	49.4044583816
H120	14.5166415224	-9.5046474550	50.6097774355
N121	11.3982288880	-9.4451153009	50.7987997016
H122	10.7387597515	-9.5018916076	51.5874046680
H123	11.3785788868	-8.5046135522	50.3928256494
H124	19.1858464913	-8.2119225773	61.7181603569
C125	19.5978936762#	-7.2298790990#	61.9900505053#
H126	20.6417031551	-7.3754802107	62.2873860583
C127	18.8496160880	-6.6536492615	63.1931412196
O128	19.4103920034	-6.0916694517	64.1302142852
C129	19.4918713806#	-6.2717986729#	60.7990632002#
H130	19.8869254012	-5.2900055722	61.0840492172
H131	18.4372716564	-6.1090465446	60.5557563670
C132	20.2126349523	-6.7816312900	59.5363762637
H133	19.9410921713	-7.8254564299	59.3368514926
H134	21.3010551086	-6.7874789936	59.6960600645
C135	19.9755437932	-6.0473947409	58.2072830765

O136	20.2658241240	-6.5682177054	57.1415146894
O137	19.4683891240	-4.8225090598	58.3388254109
N138	17.4942913033	-6.7889032576	63.0896095282
H139	17.1351385337	-7.1816649614	62.2285144358
C140	16.5368852328#	-6.0058845917#	63.8530407082#
H141	15.8898077517	-6.6816286494	64.4303669474
H142	17.1069623526	-5.4031193211	64.5641305436
C143	15.6652278402	-5.1100686329	62.9304823489
H144	16.2518105631	-4.2481633168	62.5955517925
H145	14.8282373680	-4.7235224863	63.5276731482
C146	15.1559182338	-5.8609574141	61.7106063739
C147	14.2083326397	-6.8927669212	61.8206044894
H148	13.7524948851	-7.1044428054	62.7864583773
C149	15.7096651414	-5.5953201511	60.4463891917
H150	16.4132341413	-4.7726702988	60.3413954609
C151	13.8503268693	-7.6588100207	60.7075725827
H152	13.1232429098	-8.4603191763	60.8170933773
C153	15.3597643927	-6.3656632986	59.3314168304
H154	15.8144644935	-6.1659743479	58.3646823864
C155	14.4380137423	-7.4095601092	59.4617002979
H156	14.2078056268	-8.0300071108	58.5984466957
H157	9.0037559974#	0.7050734413#	60.7752692846#
C158	8.1269804479#	0.1560798783#	60.4649994519#
H159	7.2836720528#	0.4592420476#	61.0677700552#
H160	10.4102506237	-6.3733604779	57.8145473071
H161	18.2592784963#	-9.4987526010#	50.1505400692#
C162	17.5839749087#	-8.6559204429#	50.1520271367#
H163	17.2773627230#	-8.4419890622#	49.1387987686#
H164	23.2880008521	-4.2661884732	52.5141570707
H165	10.8757455998	-5.2971469552	50.7133729567
H166	10.6807718564	-8.6476727368	57.3835146325
H167	13.5791994222	-5.0104195065	57.8402358684
C168	6.7634245923	-8.6095915423	56.0020899019
H169	7.2405988286	-8.9091843154	55.0624441707
H170	5.9657966275	-7.8953434168	55.7583297014
N171	11.4162595557	-7.9916868865	57.0988882118
N172	10.8354393805	-6.6733863900	56.9351252672
H173	7.1493939836#	-5.8354099142#	59.8218942854#
C174	6.9590151926#	-4.8009332401#	59.5000036209#
H175	5.9039259960#	-4.4903550103#	59.5181449454#
H176	7.4977934071	-4.1652062345	60.2168186044
C177	7.4425998185	-4.4741838294	58.0833246778
H178	8.4770072270	-4.8125378121	57.9279899763
H179	7.4420526618	-3.3834706054	57.9582611875
O180	6.5857104293	-4.9915118164	57.0817180226
H181	6.9062477979	-5.9083042176	56.9289836951
H182	12.1442838007	-7.9777120557	57.8151714694

The N_2H_3 starting structure in E_6 with without water

Energies: E= -8030.997626, solv = -0.196030, disp = --245.20, Z_0 = 910.30

Mo1	16.6234407100	-5.5272560808	53.1405143237
Fe2	9.3250215704	-6.6320759806	55.6139404942
Fe3	11.7023942234	-5.1346997466	55.6183904803
Fe4	11.0488742403	-6.3334430096	53.2488978146
Fe5	12.2447358989	-7.9526689714	55.1678595143
Fe6	14.7510313000	-7.5047223066	54.0887097679
Fe7	14.2916599448	-5.0522639146	54.9179670377
Fe8	13.3951438664	-5.7265013864	52.3343771320
C9	17.8623324627	-2.0014928314	54.2165218000
H10	17.9949502809	-0.9733713976	53.8737378372
H11	16.7837593807	-2.1753967113	54.3233205802
C12	18.4180874403	-3.0055005987	53.1822305397
C13	19.8893949885	-2.6757742566	52.8388330183
H14	20.4981649532	-2.9154062761	53.7151521323
H15	19.9683931795	-1.6000058953	52.6517535780
C16	20.4037776454	-3.4393560536	51.5953550318
H17	20.0612435630	-2.9582215171	50.6794404116
H18	20.0062516048	-4.4597245356	51.6225140532
C19	17.6693911248	-3.1177780065	51.8166534148
O20	17.3604198649	-4.3418448249	51.4836978001
O21	17.5542437346	-2.1193605692	51.1090525556
O22	18.3445152701	-4.3110749522	53.7896417219
C23	12.7980202958	-6.3013991586	54.2475842883
S24	13.3665515921	-3.7005505788	56.6845297809
S25	16.2573890577	-6.2894047008	55.4759248434
S26	9.6148521508	-4.6819000282	54.3708147676
S27	14.0914820835	-9.5610422757	55.3506957529
S28	14.8148969117	-3.7818318393	53.0366773032
S29	10.1412967013	-8.7544473719	54.1549363062
S30	15.2490691927	-7.1519352479	51.8699832604
S31	11.4504857515	-6.1379024715	50.9668518235
H32	13.6351742069#	-2.1902104791#	49.0425559787#
C33	13.2979243399#	-2.0241175466#	50.0550235653#
H34	13.9519807795	-2.5971442644	50.7202455823
H35	13.3740882352	-0.9571628762	50.3005083645
N36	11.9101037745	-2.4899016124	50.1428800056
H37	11.3497378639	-2.4789579286	49.3024911570
C38	11.2677826877	-2.8883419971	51.2460361194
N39	11.8323226023	-2.8477240125	52.4404410270
H40	11.3554350337	-3.3515341600	53.1907378728
H41	12.8516777746	-2.7878892351	52.5528613738
N42	9.9478705441	-3.2468809791	51.1215994249
H43	9.8508312890	-3.9805541672	50.4189656964
H44	9.5803469178	-3.6247829485	52.0007438891
H45	7.9294896359	0.3897630546	59.4133108767
C46	8.3265194018	-1.3538295238	60.6910269003
H47	8.2921991765	-1.5576322326	61.7679702127

H48	7.4842587534	-1.8945919270	60.2406588700
C49	9.6320557899	-1.8818648781	60.1858505267
N50	10.6836742392	-2.1089068571	61.0531157264
C51	10.0176772650	-2.1617566789	58.8937446655
H52	9.4944351994	-2.1302546292	57.9511210375
C53	11.6808244553	-2.5177030396	60.2895169267
H54	12.6696345371	-2.7920048704	60.6307044381
N55	11.3298880894	-2.5631823713	58.9772432242
H56	19.2544726650	-4.3174278612	57.4853368098
H57	16.7368608506	-8.8308967262	50.8061592182
C58	18.4218208969	-7.4260615115	50.5775398999
H59	17.8220004686	-6.5150187740	50.5152434397
H60	19.2393610826	-7.3138326386	49.8511664367
C61	19.0142108679	-7.5183053059	51.9484021773
N62	18.3736946204	-6.9992462435	53.0612808768
C63	20.1978610798	-8.0861575162	52.3541747097
H64	20.9797915566	-8.5857046823	51.8035131913
C65	19.1425696592	-7.2449634662	54.1096214446
H66	18.9528075891	-6.9421775282	55.1278473664
N67	20.2545675707	-7.9118260236	53.7269453450
H68	21.0142926976	-8.1487265177	54.3473366559
H69	6.9348977666	-10.5754928606	56.9408183981
C70	6.1750548248#	-9.8249631846#	56.6900007877#
H71	5.5309553636#	-10.2322281265#	56.0424861033#
H72	5.6932325284#	-9.5371411777#	57.6127204862#
S73	8.1417248548	-7.9691248669	57.1548603637
H74	11.9236455965	-2.8452925048	58.1937918867
H75	18.5169775277	-4.1391302946	54.7968366207
C76	18.4834108129	-2.1412591751	55.6374320033
O77	18.7961430146	-3.3376935925	56.0224705731
O78	18.6025546634	-1.1097195142	56.3240704096
C79	21.8965080655	-3.5311675788	51.5778401103
O80	22.6822413900	-3.0527224633	50.7821135535
O81	22.3208169442#	-4.2658019617#	52.6398596010#
H82	15.7024655187#	-0.1996320066#	61.4912314413#
C83	14.8549938543#	-0.0829906873#	60.8320018841#
H84	13.9165015893#	-0.2867338795#	61.3260937710#
H85	14.8232140057	0.9910247350	60.5815645187
C86	15.0599853617#	-0.8751945887#	59.5441990514#
H87	14.3331904110	-0.5560678161	58.7874332098
H88	14.8719959516	-1.9401873623	59.7240973635
C89	16.4776295447	-0.7309574203	58.9766771420
H90	16.7968366908	0.3217733114	59.0028642146
H91	16.5129383485	-1.0161875275	57.9195444567
C92	17.5241754502	-1.5655695726	59.7278852678
O93	17.3007544216	-2.0899754516	60.8268173472
N94	18.7225747309	-1.6671542710	59.1000488136
H95	19.3416602606	-2.3790842947	59.4681524356
H96	18.7925437917	-1.4625959727	58.0899539477
C97	10.6519926700#	0.0100028032#	53.3460013467#

H98	11.1636988811#	0.9592721168#	53.2872698518#
H99	9.5863320763#	0.1850135028#	53.3579374136#
H100	10.9321452661	-0.6017893677	52.4923392158
C101	11.0764274249	-0.5853691549	54.7050132876
H102	10.7779527766	-1.6403862083	54.7551292861
C103	10.3488110296	0.1568711497	55.8361862781
H104	9.2606024535	0.0332968372	55.7591691639
H105	10.5657707784	1.2344059408	55.7978337171
H106	10.6602407356	-0.2075382263	56.8209562019
C107	12.5961501377	-0.4879761450	54.8990414380
H108	13.1502312244	-1.0067280822	54.1099445565
H109	12.9089564108	-0.9251250316	55.8523791662
H110	12.9134259326	0.5649770497	54.8906588704
H111	13.4550779738#	-12.2303882532#	53.1883141735#
C112	13.9110015444#	-11.6210557941#	52.4219865616#
H113	14.8163061232	-11.1458214912	52.8075160546
H114	14.1145733705	-12.1972129708	51.5159965486
N115	12.8234881075	-10.6579348552	52.1866034686
H116	12.1474884357	-10.5574729273	52.9386018027
C117	12.6598829854	-9.7985098114	51.1756516127
N118	13.6898621219	-9.5317894780	50.3344173650
H119	13.4650857519	-8.7990592045	49.6674963323
H120	14.5566347368	-9.2854981554	50.8203069724
N121	11.4590361704	-9.2538807069	50.9461382831
H122	10.7326029713	-9.4935306381	51.6174982962
H123	11.4544126280	-8.2148397000	50.7458398660
H124	19.2004023808	-8.2166858232	61.7115706712
C125	19.5978936406#	-7.2298791114#	61.9900505340#
H126	20.6402488478	-7.3669058921	62.2960994050
C127	18.8359902761	-6.6844840464	63.1963889456
O128	19.3864558831	-6.2198772391	64.1910673999
C129	19.4918713789#	-6.2717986867#	60.7990632100#
H130	19.8787913815	-5.2876780771	61.0885273341
H131	18.4371160689	-6.1081232085	60.5550967251
C132	20.2080062623	-6.7539534074	59.5247744958
H133	19.9459385393	-7.7968333267	59.3080557410
H134	21.2978015912	-6.7505717756	59.6737767371
C135	19.9495184482	-5.9966176348	58.2088234640
O136	20.2361189812	-6.5012220588	57.1347953934
O137	19.4324477405	-4.7783817060	58.3629952497
N138	17.4805683363	-6.7478200282	63.0393938741
H139	17.1307950764	-7.0505136659	62.1394564575
C140	16.5368852577#	-6.0058845605#	63.8530406918#
H141	15.8216588646	-6.7066362596	64.3070267553
H142	17.1068400422	-5.5438068753	64.6626113291
C143	15.7635836662	-4.9388130195	63.0343179650
H144	16.4118631277	-4.0769370938	62.8452221868
H145	14.9231264262	-4.5866203903	63.6478488246
C146	15.2757127492	-5.4844841694	61.7039311931
C147	14.2916010158	-6.4833593142	61.6319608160

H148	13.7886038799	-6.8130306914	62.5394647996
C149	15.8958531840	-5.0640395359	60.5156253581
H150	16.6310855060	-4.2647120101	60.5568448825
C151	13.9617079100	-7.0721739751	60.4074309467
H152	13.2080568337	-7.8557970864	60.3748194919
C153	15.5715935577	-5.6534760910	59.2900534641
H154	16.0715270054	-5.3407212736	58.3776198809
C155	14.6130679691	-6.6684601439	59.2366684638
H156	14.4039600046	-7.1412930557	58.2802946053
H157	9.0037560240#	0.7050733933#	60.7752692938#
C158	8.1269804050#	0.1560799474#	60.4649993725#
H159	7.2836720886#	0.4592420427#	61.0677701079#
H160	10.5037997705	-5.9870449016	57.9502532934
H161	18.2592785481#	-9.4987525595#	50.1505400650#
C162	17.5839748191#	-8.6559205050#	50.1520271819#
H163	17.2773627187#	-8.4419890476#	49.1387987730#
H164	23.2948654431	-4.2622438550	52.5881913049
H165	9.5784100791	-9.3104672160	55.2555924886
H166	10.9761825818	-8.2791442138	57.6095678842
H167	13.6395258101	-4.5326069532	57.7164605712
C168	6.8313282303	-8.6196276002	56.0467606153
H169	7.2657875892	-8.8941038500	55.0787044586
H170	6.0868557980	-7.8370214158	55.8568984393
N171	11.5885719842	-7.5518218521	57.2235038184
N172	10.8406810579	-6.3200701273	57.0436831849
H173	7.1493940418#	-5.8354098993#	59.8218942987#
C174	6.9590151903#	-4.8009333192#	59.5000035735#
H175	5.9039259907#	-4.4903550254#	59.5181448964#
H176	7.5039128916	-4.1575133949	60.2035202644
C177	7.4414614925	-4.5132188689	58.0773936784
H178	8.5028377141	-4.7722594485	57.9572517386
H179	7.3551079149	-3.4394620194	57.8755178418
O180	6.6462720521	-5.1683811943	57.0996012394
H181	6.9714110496	-6.0939101433	57.0919318426
H182	14.6643745946	-9.1988485509	56.5178903830
H183	12.3592834216	-7.4070110489	57.8771840215

The N_2H_3 starting structure in E_6 with two waters
Energies: E= -8183.862887, solv = -0.208813, disp = --256.85, Z_0 = 942.52

Mo1	16.3889043741	-5.8480564528	53.2515498074
Fe2	8.9152156717	-7.2014027978	54.6056962586
Fe3	11.2494066681	-5.7651852240	55.4718257644
Fe4	10.9857169248	-6.8748908275	52.9600700974
Fe5	11.8494997550	-8.5307065122	54.9006042542
Fe6	14.4650700939	-7.8644651189	54.2104957108
Fe7	13.9457843881	-5.3995802859	55.0816936267
Fe8	13.3389813253	-5.8657583404	52.4980228971
C9	17.7505846367	-2.2288637362	54.1126664937

H10	17.9338424106	-1.2410307848	53.6858809314
H11	16.6669431441	-2.3321446942	54.2494925967
C12	18.2282853715	-3.3388480218	53.1542974958
C13	19.7071162071	-3.1187396565	52.7637886036
H14	20.3206965126	-3.2379438358	53.6613658800
H15	19.8091204318	-2.0853736593	52.4145062222
C16	20.1921599370	-4.0760859339	51.6565385426
H17	19.7186261763	-3.8445023539	50.7010945230
H18	19.9259394943	-5.1024028833	51.9351994685
C19	17.4396684707	-3.4807266087	51.8193898607
O20	17.0553564367	-4.6986958696	51.5522028965
O21	17.3434598121	-2.5130773410	51.0666872555
O22	18.0918755054	-4.5903187390	53.8597898675
C23	12.5552870943	-6.7319889256	54.1739094128
S24	12.6458255270	-4.0758019185	56.6026064782
S25	15.9632902718	-6.5986088940	55.6002505131
S26	9.4913183995	-5.0925062726	53.8366530618
S27	13.7737440851	-10.0152035607	55.1734628974
S28	14.6239007047	-4.0165645430	53.2714480978
S29	9.9123477863	-9.0090318841	53.4333555297
S30	15.0341318189	-7.4978648224	51.9730695850
S31	11.7530472311	-6.3265485325	50.6144923938
H32	13.6351742803#	-2.1902106846#	49.0425560370#
C33	13.2979241946#	-2.0241172990#	50.0550235752#
H34	13.8638922619	-2.6995107993	50.7017472136
H35	13.5021422425	-0.9901000023	50.3543260444
N36	11.8628258774	-2.3274339665	50.1223827602
H37	11.2857547028	-1.9619010907	49.3781336654
C38	11.1918284763	-2.8115945237	51.1844096401
N39	11.7625588203	-3.0763646530	52.3422800754
H40	11.2275589740	-3.6265566567	53.0254608430
H41	12.7802796554	-3.0598039547	52.5005297233
N42	9.8409415506	-2.9838943311	51.0721579853
H43	9.4855740382	-3.2052920983	50.1510243907
H44	9.4141519284	-3.5351826921	51.8250963433
H45	7.9310894666	0.3935244544	59.4136565230
C46	8.3415061989	-1.3539493108	60.6812942570
H47	8.2227440126	-1.5798260602	61.7477657735
H48	7.5606043880	-1.9104377686	60.1494076164
C49	9.7042070337	-1.8203914206	60.2787814312
N50	10.7162683576	-1.9185767872	61.2157359836
C51	10.1883357090	-2.1245030270	59.0272630804
H52	9.7230543511	-2.1856891547	58.0561480149
C53	11.7875138337	-2.2806085310	60.5349286620
H54	12.7666511967	-2.4778617102	60.9463422247
N55	11.5226087288	-2.4090801471	59.2074074927
H56	19.3479975994	-4.3425482054	57.4609612549
H57	16.7238696902	-8.8543884279	50.7849303897
C58	18.3977663752	-7.4477905149	50.6579544034
H59	17.8024378031	-6.5329210181	50.6095061933

H60	19.2565387950	-7.3043522757	49.9872834571
C61	18.9108316920	-7.6164731708	52.0555365294
N62	18.1530668135	-7.2741259515	53.1666562952
C63	20.1204265564	-8.1002130682	52.4925686998
H64	20.9813083669	-8.4660311914	51.9543414293
C65	18.8824517921	-7.5378256823	54.2390428305
H66	18.6005541406	-7.3369625458	55.2612881259
N67	20.0778145785	-8.0524788930	53.8764993186
H68	20.8290037519	-8.2399666781	54.5241682946
H69	7.0118896192	-10.4986712661	56.8946487233
C70	6.1750549373#	-9.8249630355#	56.6900008260#
H71	5.5309553967#	-10.2322280894#	56.0424860470#
H72	5.6932324613#	-9.5371413192#	57.6127204953#
S73	7.9421426384	-7.6138460950	56.6863563362
H74	12.1544999792	-2.7078823712	58.4692451766
H75	18.2900643478	-4.3540663825	54.8518964940
C76	18.3935290218	-2.2910063705	55.5261073720
O77	18.6571657779	-3.4684595513	55.9955265233
O78	18.5726402584	-1.2142119890	56.1229351852
C79	21.6786102499	-4.0323989367	51.4643200153
O80	22.2958627209	-3.8369462463	50.4355106695
O81	22.3208169402#	-4.2658019525#	52.6398596093#
H82	15.7024655276#	-0.1996319904#	61.4912314328#
C83	14.8549940146#	-0.0829906635#	60.8320018961#
H84	13.9165015893#	-0.2867338795#	61.3260937711#
H85	14.8184842761	0.9909440246	60.5798420836
C86	15.0599852418#	-0.8751946635#	59.5441990242#
H87	14.2575250978	-0.6568728697	58.8275521844
H88	15.0226951930	-1.9492907076	59.7430984183
C89	16.4120266723	-0.5299609393	58.8796292813
H90	16.4634415961	0.5600985038	58.7301985527
H91	16.4794242491	-0.9707957774	57.8794906171
C92	17.6679976680	-0.9202376952	59.6807826428
O93	17.7318284751	-0.9078965995	60.9163117471
N94	18.7379711328	-1.2518584262	58.9182302461
H95	19.5746461256	-1.5264427790	59.4146405800
H96	18.7220448913	-1.2982393314	57.8873950717
C97	10.6519927804#	0.0100027657#	53.3460013419#
H98	11.1636988498#	0.9592721337#	53.2872698538#
H99	9.5863320748#	0.1850134940#	53.3579374138#
H100	10.9194472937	-0.5825118849	52.4697228680
C101	11.0905626744	-0.6409195971	54.6716844924
H102	10.7466588776	-1.6835319065	54.6946685594
C103	10.4343125819	0.0956797033	55.8487987910
H104	9.3395316862	0.0334403652	55.7984558571
H105	10.7094539150	1.1602686972	55.8438049235
H106	10.7496355211	-0.3222108158	56.8101918320
C107	12.6184767827	-0.6271999711	54.8138872851
H108	13.1168183775	-1.1345904783	53.9811311970
H109	12.9371106626	-1.1331498411	55.7303098570

H110	12.9907057345	0.4069145686	54.8476214544
H111	13.4550781601#	-12.2303881871#	53.1883143368#
C112	13.9110012715#	-11.6210558751#	52.4219863518#
H113	14.7505907588	-11.0620245142	52.8377494146
H114	14.2234128637	-12.2241881097	51.5644933834
N115	12.7964819144	-10.7452661486	52.0244587470
H116	12.0711841751	-10.5813349091	52.7242956406
C117	12.6755711285	-10.0510072412	50.8999203415
N118	13.7535858580	-9.8955124659	50.0830112359
H119	13.5733364831	-9.3005359204	49.2814100466
H120	14.5821720202	-9.5673342050	50.5881276289
N121	11.4679293273	-9.5799711845	50.5281185276
H122	10.7410874548	-9.6258464703	51.2547017785
H123	11.4711892873	-8.6518249145	50.0963261592
H124	19.2565510644	-8.2309004411	61.6869828635
C125	19.5978936649#	-7.2298790763#	61.9900506464#
H126	20.6357996243	-7.3283128540	62.3247821410
C127	18.7874947155	-6.7756880640	63.2000043888
O128	19.2927333738	-6.5461580786	64.2967202391
C129	19.4918712053#	-6.2717987136#	60.7990631885#
H130	19.8139162280	-5.2682467037	61.0995177348
H131	18.4465651629	-6.1579390949	60.4957563105
C132	20.2960552399	-6.7193745384	59.5587237411
H133	20.1347149042	-7.7851411827	59.3595602013
H134	21.3741773632	-6.6108730780	59.7500516794
C135	20.0129587474	-6.0147930221	58.2184092903
O136	20.2107063068	-6.5790841381	57.1530213425
O137	19.5885333674	-4.7581205710	58.3433518654
N138	17.4482501011	-6.6782553920	62.9513947872
H139	17.1467565434	-6.7266795594	61.9870617672
C140	16.5368852988#	-6.0058845142#	63.8530404803#
H141	15.5870837356	-6.5549563974	63.8581375480
H142	16.9725827949	-6.0726404977	64.8530470121
C143	16.2707512233	-4.5292699861	63.4700954478
H144	17.1797072889	-3.9442477806	63.6503291943
H145	15.4915422543	-4.1393082422	64.1392798728
C146	15.8608290114	-4.4212540451	62.0194443569
C147	14.5976712368	-4.8659276625	61.5974724309
H148	13.8566790269	-5.1659035064	62.3371264860
C149	16.7894856800	-4.0239424340	61.0502493872
H150	17.7488501133	-3.6223730644	61.3608456806
C151	14.2931985198	-4.9589183722	60.2391835999
H152	13.3321990241	-5.3499387172	59.9253575012
C153	16.4903614878	-4.1216087422	59.6880922231
H154	17.2288624621	-3.8245886561	58.9521067734
C155	15.2493460754	-4.6076298648	59.2794484681
H156	15.0330982457	-4.7113080094	58.2230362911
H157	9.0037560115#	0.7050733853#	60.7752693435#
C158	8.1269805173#	0.1560798734#	60.4649991829#
H159	7.2836720973#	0.4592419889#	61.0677701467#

H160	10.6224620882	-6.7586480224	57.9570660250
H161	18.2592786276#	-9.4987524959#	50.1505400260#
C162	17.5839748336#	-8.6559205037#	50.1520273562#
H163	17.2773626761#	-8.4419890513#	49.1387987853#
H164	23.2723478036	-4.2171437142	52.4311086982
H165	10.7816103531	-5.3939212813	50.5131516536
H166	10.0459155959	-8.6704659040	56.8643845073
H167	12.7654908359	-4.8620380408	57.6926883318
C168	6.6102923035	-8.5908759214	55.9020762954
H169	6.9389076894	-8.9272871028	54.9093772014
H170	5.7360190422	-7.9461236529	55.7454785536
N171	11.0411042716	-8.4178401458	56.8623191459
N172	11.2028126821	-6.9894011207	57.1440306780
H173	7.1493939907#	-5.8354098990#	59.8218943297#
C174	6.9590151423#	-4.8009333248#	59.5000035572#
H175	5.9039259940#	-4.4903550172#	59.5181449610#
H176	7.4902602910	-4.1762138998	60.2323816692
C177	7.4511446261	-4.4371246872	58.0939122826
H178	8.4514631217	-4.8464307803	57.8987706605
H179	7.5370719343	-3.3442489220	58.0348503786
O180	6.5362243856	-4.8236360780	57.0802415314
H181	6.8398334458	-5.7068855397	56.7828749940
H182	12.7525461817	-7.0467470398	57.7115345516
H183	13.5798458110	-8.8497775335	58.3985777866
O184	13.6710456246	-7.2412092991	58.1355458956
H185	11.5218325532	-8.9883509373	57.5650405045
H186	14.3320839985	-7.0537290600	57.4423355372
O187	13.3866155720	-9.8374027501	58.4173089846
H188	14.0981624739	-10.2222012215	58.9503398025
H189	13.7989877127	-9.8731546015	56.5297471725

The N_2H_3 TS structure in E_6 with with two waters in **Figure 4**
Energies: E= -8183.860752, solv = -0.208126, disp = --256.18, Z_0 = 940.67

Mo1	16.3652220000	-5.8546690000	53.2647350000
Fe2	8.8890520000	-7.2551240000	54.5424150000
Fe3	11.2113270000	-5.8081060000	55.4118190000
Fe4	10.9793670000	-6.9462520000	52.9325740000
Fe5	11.8254830000	-8.6069370000	54.8700470000
Fe6	14.4569690000	-7.8914260000	54.2122370000
Fe7	13.9166000000	-5.4460440000	55.1057020000
Fe8	13.3238410000	-5.8952320000	52.5055630000
C9	17.7244150000	-2.2214460000	54.1533940000
H10	17.9039780000	-1.2360340000	53.7196530000
H11	16.6413790000	-2.3278300000	54.2939390000
C12	18.1995980000	-3.3340910000	53.2021360000
C13	19.6826610000	-3.1270700000	52.8179870000
H14	20.2923070000	-3.2567480000	53.7170200000
H15	19.7970640000	-2.0935540000	52.4728750000

C16	20.1593960000	-4.0863470000	51.7096750000
H17	19.6701310000	-3.8632840000	50.7601300000
H18	19.9053060000	-5.1129700000	51.9973890000
C19	17.4093590000	-3.4624660000	51.8709320000
O20	17.0397680000	-4.6797420000	51.5831120000
O21	17.2926550000	-2.4807860000	51.1403780000
O22	18.0503770000	-4.5833930000	53.9068480000
C23	12.5203400000	-6.7854630000	54.1686490000
S24	12.5546070000	-4.1539300000	56.6193720000
S25	15.9385760000	-6.6162910000	55.6022990000
S26	9.4623330000	-5.1544070000	53.7706120000
S27	13.7654840000	-10.0318190000	55.2204840000
S28	14.5809610000	-4.0435300000	53.2900010000
S29	9.8973970000	-9.0805770000	53.4103130000
S30	15.0225770000	-7.5203640000	51.9760680000
S31	11.7568410000	-6.3724100000	50.6004920000
H32	13.6351740000	-2.1902110000	49.0425560000
C33	13.2979240000	-2.0241170000	50.0550240000
H34	13.8610130000	-2.6993050000	50.7044130000
H35	13.4990200000	-0.9900290000	50.3554330000
N36	11.8612940000	-2.3289550000	50.1085430000
H37	11.2944520000	-1.9717090000	49.3528110000
C38	11.1768750000	-2.8295430000	51.1538300000
N39	11.7315430000	-3.0920710000	52.3203430000
H40	11.1927050000	-3.6475580000	52.9945450000
H41	12.7463360000	-3.0681340000	52.4932180000
N42	9.8309550000	-3.0174890000	51.0175360000
H43	9.4921190000	-3.2353720000	50.0896760000
H44	9.3965350000	-3.5750170000	51.7601900000
H45	7.9315500000	0.3887830000	59.4123960000
C46	8.3527990000	-1.3539980000	60.6921860000
H47	8.2012520000	-1.5739750000	61.7559020000
H48	7.5934060000	-1.9200080000	60.1399580000
C49	9.7305670000	-1.8238910000	60.3394890000
N50	10.7187470000	-1.8872770000	61.3051420000
C51	10.2457640000	-2.1854420000	59.1147500000
H52	9.8055320000	-2.2896060000	58.1350500000
C53	11.8056910000	-2.2838970000	60.6691380000
H54	12.7725750000	-2.4708340000	61.1140240000
N55	11.5730080000	-2.4698370000	59.3417580000
H56	19.3443070000	-4.3347390000	57.4801010000
H57	16.7217740000	-8.8596700000	50.7813570000
C58	18.3908830000	-7.4483060000	50.6716930000
H59	17.7930050000	-6.5349110000	50.6232450000
H60	19.2543400000	-7.2984030000	50.0085240000
C61	18.8950760000	-7.6202750000	52.0732440000
N62	18.1369610000	-7.2643640000	53.1803260000
C63	20.0991440000	-8.1120260000	52.5181780000
H64	20.9597910000	-8.4868010000	51.9852800000
C65	18.8611000000	-7.5262480000	54.2570750000

H66	18.5777530000	-7.3186250000	55.2775880000
N67	20.0531650000	-8.0528820000	53.9011640000
H68	20.7993250000	-8.2444590000	54.5533520000
H69	7.0158910000	-10.4946070000	56.8908510000
C70	6.1750550000	-9.8249630000	56.6900010000
H71	5.5309550000	-10.2322280000	56.0424860000
H72	5.6932320000	-9.5371410000	57.6127200000
S73	7.9421860000	-7.6046810000	56.6492830000
H74	12.2216040000	-2.7888510000	58.6285010000
H75	18.2474270000	-4.3495280000	54.8992210000
C76	18.3786990000	-2.2792240000	55.5587060000
O77	18.6354650000	-3.4549770000	56.0344750000
O78	18.5755590000	-1.1999970000	56.1435770000
C79	21.6416020000	-4.0303630000	51.4858470000
O80	22.2276150000	-3.8222210000	50.4416030000
O81	22.3208170000	-4.2658020000	52.6398600000
H82	15.7024660000	-0.1996320000	61.4912310000
C83	14.8549940000	-0.0829910000	60.8320020000
H84	13.9165020000	-0.2867340000	61.3260940000
H85	14.8178730000	0.9911790000	60.5804410000
C86	15.0599850000	-0.8751950000	59.5441990000
H87	14.2547540000	-0.6610960000	58.8293350000
H88	15.0296090000	-1.9503320000	59.7443450000
C89	16.4074390000	-0.5217990000	58.8755150000
H90	16.4459550000	0.5670090000	58.7135370000
H91	16.4798930000	-0.9734600000	57.8806090000
C92	17.6662470000	-0.8876440000	59.6826810000
O93	17.7246120000	-0.8674000000	60.9178270000
N94	18.7444740000	-1.2050490000	58.9276050000
H95	19.5808950000	-1.4685420000	59.4302280000
H96	18.7317770000	-1.2649070000	57.8978570000
C97	10.6519930000	0.0100030000	53.3460010000
H98	11.1636990000	0.9592720000	53.2872700000
H99	9.5863320000	0.1850130000	53.3579370000
H100	10.9188710000	-0.5795570000	52.4666020000
C101	11.0865880000	-0.6563840000	54.6676550000
H102	10.7426470000	-1.6994230000	54.6739300000
C103	10.4259490000	0.0592000000	55.8563490000
H104	9.3310350000	0.0019310000	55.8005320000
H105	10.7050250000	1.1225560000	55.8750380000
H106	10.7359740000	-0.3802580000	56.8108420000
C107	12.6143160000	-0.6469190000	54.8200100000
H108	13.1179440000	-1.1386040000	53.9810290000
H109	12.9248540000	-1.1713800000	55.7294870000
H110	12.9883230000	0.3854850000	54.8765430000
H111	13.4550780000	-12.2303880000	53.1883140000
C112	13.9110010000	-11.6210560000	52.4219860000
H113	14.7347830000	-11.0424940000	52.8437590000
H114	14.2455050000	-12.2266920000	51.5744450000
N115	12.7880020000	-10.7685250000	52.0028750000

H116	12.0497440000	-10.6155040000	52.6907990000
C117	12.6711220000	-10.0835650000	50.8731750000
N118	13.7535090000	-9.9278410000	50.0630660000
H119	13.5770160000	-9.3402030000	49.2554320000
H120	14.5791160000	-9.5966930000	50.5704620000
N121	11.4625820000	-9.6250120000	50.4867730000
H122	10.7306680000	-9.6770890000	51.2061410000
H123	11.4642300000	-8.6963580000	50.0558800000
H124	19.2521500000	-8.2297660000	61.6885710000
C125	19.5978940000	-7.2298790000	61.9900510000
H126	20.6366140000	-7.3309440000	62.3216440000
C127	18.7926360000	-6.7696990000	63.1996910000
O128	19.3029520000	-6.5378010000	64.2930710000
C129	19.4918710000	-6.2717990000	60.7990630000
H130	19.8081350000	-5.2670980000	61.1013530000
H131	18.4471650000	-6.1624020000	60.4918290000
C132	20.3016300000	-6.7161430000	59.5641460000
H133	20.1514180000	-7.7839140000	59.3681460000
H134	21.3783920000	-6.5923170000	59.7541400000
C135	19.9999660000	-6.0160670000	58.2282830000
O136	20.1609470000	-6.5907180000	57.1628510000
O137	19.5995010000	-4.7523790000	58.3570700000
N138	17.4546010000	-6.6689820000	62.9526870000
H139	17.1499890000	-6.7285990000	61.9900780000
C140	16.5368850000	-6.0058850000	63.8530400000
H141	15.5922150000	-6.5632500000	63.8508290000
H142	16.9687390000	-6.0749420000	64.8550600000
C143	16.2522420000	-4.5305930000	63.4803590000
H144	17.1457810000	-3.9255030000	63.6706100000
H145	15.4578760000	-4.1640290000	64.1453710000
C146	15.8534870000	-4.4403750000	62.0271830000
C147	14.6332080000	-4.9849250000	61.5983090000
H148	13.9097440000	-5.3315050000	62.3355800000
C149	16.7657910000	-3.9922260000	61.0662720000
H150	17.6925590000	-3.5238900000	61.3836370000
C151	14.3578780000	-5.1340870000	60.2404460000
H152	13.4549800000	-5.6311690000	59.9082670000
C153	16.4914870000	-4.1333760000	59.7027460000
H154	17.2159280000	-3.7961460000	58.9695360000
C155	15.2997910000	-4.7265220000	59.2892140000
H156	15.1071820000	-4.8745320000	58.2327190000
H157	9.0037560000	0.7050730000	60.7752690000
C158	8.1269810000	0.1560800000	60.4649990000
H159	7.2836720000	0.4592420000	61.0677700000
H160	10.5065180000	-6.8487100000	57.9045110000
H161	18.2592790000	-9.4987520000	50.1505400000
C162	17.5839750000	-8.6559210000	50.1520270000
H163	17.2773630000	-8.4419890000	49.1387990000
H164	23.2647780000	-4.2060020000	52.4019180000
H165	10.7738050000	-5.4513330000	50.5012230000

H166	10.0211880000	-8.7343860000	56.8221410000
H167	12.7381260000	-4.9651330000	57.6876360000
C168	6.5950770000	-8.5872840000	55.8967500000
H169	6.9021820000	-8.9231390000	54.8964950000
H170	5.7171900000	-7.9438740000	55.7580780000
N171	11.0183570000	-8.4965050000	56.8317200000
N172	11.1598750000	-7.0744100000	57.1475340000
H173	7.1493940000	-5.8354100000	59.8218940000
C174	6.9590150000	-4.8009330000	59.5000040000
H175	5.9039260000	-4.4903550000	59.5181450000
H176	7.4877270000	-4.1783360000	60.2361560000
C177	7.4587760000	-4.4300870000	58.0987820000
H178	8.4588090000	-4.8424910000	57.9072910000
H179	7.5500300000	-3.3372660000	58.0478930000
O180	6.5485480000	-4.8035740000	57.0759440000
H181	6.8500140000	-5.6835550000	56.7680710000
H182	12.3116710000	-7.0488390000	57.6376840000
H183	13.4456120000	-8.7000800000	58.4037100000
O184	13.4217870000	-7.1846150000	58.1915120000
H185	11.4955250000	-9.0650250000	57.5393990000
H186	14.1144670000	-7.0096650000	57.5309360000
O187	13.3343980000	-9.7264340000	58.4063960000
H188	14.0887290000	-10.0627470000	58.9119510000
H189	13.7410850000	-9.8532950000	56.5818310000

The N_2H_4 product structure in E_6 with two waters in **Figure 5**
Energies: E= -8183.895595, solv = -0.210424, disp = --256.66, Z_0 = 946.39

Mo1	16.3651371802	-5.8545877315	53.2604384481
Fe2	8.9137481273	-7.1892401824	54.6286005182
Fe3	11.2746766529	-5.7473816785	55.4102587795
Fe4	10.9653399221	-6.8964539087	53.0003549247
Fe5	11.8810445429	-8.6526943704	54.8980916857
Fe6	14.4401692857	-7.9518118361	54.2439337777
Fe7	13.9496085068	-5.4543293469	55.0992387522
Fe8	13.3214319600	-5.8869438994	52.5199693544
C9	17.7508079413	-2.2413823274	54.1462092238
H10	17.9392683517	-1.2527127243	53.7236617817
H11	16.6664150746	-2.3395681869	54.2825398335
C12	18.2211166381	-3.3496671658	53.1816349251
C13	19.7019699070	-3.1377082850	52.7931955622
H14	20.3146861704	-3.2705040518	53.6895521584
H15	19.8124000634	-2.1017911508	52.4540491981
C16	20.1795143546	-4.0875824177	51.6765940669
H17	19.6983556122	-3.8505821200	50.7262897293
H18	19.9158100698	-5.1160379224	51.9502899856
C19	17.4300849730	-3.4774242100	51.8453016789
O20	17.0371331093	-4.6890154698	51.5673205541
O21	17.3404914331	-2.5010590567	51.1019166796

O22	18.0756677783	-4.6034715877	53.8790604980
C23	12.5195973632	-6.7466001343	54.2022443462
S24	12.6255832055	-4.1212362483	56.6135841417
S25	15.9531575644	-6.6216486004	55.6088922638
S26	9.4872952501	-5.0764315753	53.8791119440
S27	13.7369845300	-9.9873501138	55.1925248724
S28	14.5941161915	-4.0263351435	53.2953550469
S29	9.8572483726	-9.0106884243	53.4738823586
S30	15.0060606343	-7.5165655909	51.9922209003
S31	11.7073460995	-6.3352618245	50.6542345753
H32	13.6351742804#	-2.1902106818#	49.0425560366#
C33	13.2979241974#	-2.0241173044#	50.0550235736#
H34	13.8556519190	-2.7080329302	50.6999979324
H35	13.5133829654	-0.9937654523	50.3587207226
N36	11.8602146930	-2.3123761064	50.1206021185
H37	11.2850795088	-1.9312109758	49.3826680094
C38	11.1875519536	-2.8030790632	51.1780386540
N39	11.7551495400	-3.0814256561	52.3334536128
H40	11.2167508998	-3.6359548274	53.0099258356
H41	12.7741478308	-3.0758257574	52.4982347460
N42	9.8341401168	-2.9646391542	51.0610872183
H43	9.4843666742	-3.1938297712	50.1395740949
H44	9.4014465975	-3.5101425172	51.8129218262
H45	7.9306278587	0.3953060687	59.4141289874
C46	8.3416660853	-1.3539498854	60.6763499164
H47	8.2435557684	-1.5813864420	61.7445951393
H48	7.5502243756	-1.9088660520	60.1584324735
C49	9.6971096345	-1.8149180257	60.2455752666
N50	10.7268955182	-1.9133582594	61.1626076434
C51	10.1620206086	-2.0956361796	58.9813205898
H52	9.6802772288	-2.1450229383	58.0175544889
C53	11.7906509249	-2.2500054116	60.4577913749
H54	12.7791250348	-2.4393480780	60.8496521203
N55	11.5037820310	-2.3626182102	59.1330589558
H56	19.3377221969	-4.3505145404	57.4625981937
H57	16.7220397719	-8.8558304912	50.7828835589
C58	18.3916489452	-7.4459806625	50.6637767747
H59	17.7926186269	-6.5334332119	50.6148963335
H60	19.2541586509	-7.2972817549	49.9989553202
C61	18.8953496093	-7.6187459515	52.0642422822
N62	18.1289476500	-7.2836249540	53.1716758415
C63	20.1007134056	-8.1080711352	52.5069312656
H64	20.9646764724	-8.4726145310	51.9727519995
C65	18.8484611413	-7.5596032357	54.2474500152
H66	18.5570826959	-7.3681958180	55.2687153409
N67	20.0460662025	-8.0733841048	53.8908576570
H68	20.7916354053	-8.2659082707	54.5434592399
H69	7.0031950685	-10.5083516812	56.8997530649
C70	6.1750549227#	-9.8249630431#	56.6900008240#
H71	5.5309554013#	-10.2322280970#	56.0424860473#

H72	5.6932324655#	-9.5371413109#	57.6127204949#
S73	7.9426077649	-7.6095001197	56.7291986720
H74	12.1264263317	-2.6444573119	58.3799866172
H75	18.2706544311	-4.3790092816	54.8691659480
C76	18.3931460809	-2.3116742436	55.5597821477
O77	18.6459925413	-3.4910470879	56.0280858603
O78	18.5809231091	-1.2370485271	56.1595446711
C79	21.6641091826	-4.0432471188	51.4699547348
O80	22.2698668125	-3.8569374105	50.4323003334
O81	22.3208169348#	-4.2658019493#	52.6398596084#
H82	15.7024655277#	-0.1996319899#	61.4912314327#
C83	14.8549940156#	-0.0829906632#	60.8320018961#
H84	13.9165015893#	-0.2867338795#	61.3260937711#
H85	14.8180708736	0.9907717448	60.5793015430
C86	15.0599852422#	-0.8751946648#	59.5441990241#
H87	14.2650793546	-0.6473095108	58.8220326245
H88	15.0117682634	-1.9495556907	59.7403952698
C89	16.4216115572	-0.5417111372	58.8926695657
H90	16.4879838320	0.5486737627	58.7520616862
H91	16.4928760693	-0.9755630544	57.8898281192
C92	17.6644755689	-0.9554738680	59.7032633549
O93	17.7131618830	-0.9570626061	60.9399188291
N94	18.7377739540	-1.2930147016	58.9490122025
H95	19.5637968773	-1.5893381581	59.4508743777
H96	18.7292548178	-1.3335644702	57.9171571353
C97	10.6519927785#	0.0100027476#	53.3460013887#
H98	11.1636988392#	0.9592721398#	53.2872698575#
H99	9.5863320770#	0.1850135100#	53.3579373762#
H100	10.9202036964	-0.5837450435	52.4710166698
C101	11.0886205181	-0.6386742962	54.6734543234
H102	10.7381345737	-1.6790870568	54.7006772385
C103	10.4387706218	0.1084913197	55.8474452278
H104	9.3435673524	0.0481048493	55.8038027838
H105	10.7155214555	1.1724305548	55.8317794246
H106	10.7589702159	-0.3007904331	56.8107083953
C107	12.6163563131	-0.6345776303	54.8156581926
H108	13.1115253343	-1.1488297259	53.9855674920
H109	12.9314957905	-1.1393336249	55.7339726124
H110	12.9955917515	0.3969338441	54.8464889205
H111	13.4550781737#	-12.2303881815#	53.1883143494#
C112	13.9110012482#	-11.6210558839#	52.4219863367#
H113	14.7349284229	-11.0502253140	52.8507284434
H114	14.2287806697	-12.2221166833	51.5638725288
N115	12.7900087207	-10.7604057390	52.0200687054
H116	12.1147473072	-10.5527160616	52.7571668423
C117	12.6620570494	-10.0681947197	50.8990168551
N118	13.7286020703	-9.9239839611	50.0633806726
H119	13.5404428584	-9.3297939698	49.2632188868
H120	14.5655044369	-9.6016006268	50.5571021310
N121	11.4521893046	-9.5845049002	50.5420768740

H122	10.7327021020	-9.6322506907	51.2740662974
H123	11.4563100749	-8.6540006335	50.1168756941
H124	19.2578798279	-8.2308451285	61.6852076051
C125	19.5978936631#	-7.2298790758#	61.9900506455#
H126	20.6355962209	-7.3281384729	62.3253850818
C127	18.7859666877	-6.7806517699	63.2007614700
O128	19.2886732917	-6.5605694830	64.3005502862
C129	19.4918712020#	-6.2717987123#	60.7990631897#
H130	19.8178749024	-5.2689469131	61.0971605873
H131	18.4467275501	-6.1564928449	60.4964609420
C132	20.2939993701	-6.7252998633	59.5598313439
H133	20.1334772355	-7.7923069070	59.3671069062
H134	21.3723487798	-6.6137604360	59.7478352782
C135	20.0040099460	-6.0264172972	58.2185959684
O136	20.1913850537	-6.5965380019	57.1546958991
O137	19.5856853375	-4.7681214943	58.3439047295
N138	17.4471995104	-6.6771196676	62.9497447626
H139	17.1478057418	-6.7196638944	61.9843875618
C140	16.5368853066#	-6.0058845214#	63.8530404783#
H141	15.5884510354	-6.5575582103	63.8634595169
H142	16.9767330416	-6.0684486034	64.8515775289
C143	16.2649349810	-4.5311598433	63.4663848431
H144	17.1734500120	-3.9435065430	63.6403716120
H145	15.4881175451	-4.1406878751	64.1380558131
C146	15.8471219435	-4.4261429588	62.0173288223
C147	14.5726621338	-4.8464776649	61.6038157297
H148	13.8293647757	-5.1277305700	62.3483977190
C149	16.7773577667	-4.0497163973	61.0410932490
H150	17.7454008180	-3.6646651528	61.3454701366
C151	14.2589106195	-4.9335758559	60.2470474326
H152	13.2815506935	-5.2929180426	59.9433600395
C153	16.4697378481	-4.1461701645	59.6807847113
H154	17.2098725697	-3.8676643199	58.9393098704
C155	15.2159701332	-4.6055919555	59.2802158205
H156	14.9947157206	-4.7077487613	58.2243948524
H157	9.0037560112#	0.7050733875#	60.7752693404#
C158	8.1269805196#	0.1560798876#	60.4649991672#
H159	7.2836721012#	0.4592419830#	61.0677701552#
H160	10.6161214091	-6.8118974960	58.0394143702
H161	18.2592786336#	-9.4987524909#	50.1505400185#
C162	17.5839748466#	-8.6559205001#	50.1520273747#
H163	17.2773626673#	-8.4419890562#	49.1387987870#
H164	23.2694209952	-4.2204655969	52.4175996527
H165	10.7358390261	-5.4009109494	50.5687768334
H166	10.0389571310	-8.6232515313	56.8677892758
H167	12.7647715732	-4.8307387861	57.7539802662
C168	6.6346249941	-8.5965797086	55.9100047147
H169	6.9944925641	-8.9365816973	54.9299132070
H170	5.7685080635	-7.9484580878	55.7269688891
N171	11.0512593001	-8.4567842044	56.9043705056

N172	11.2350814448	-7.0536072714	57.2595092540
H173	7.1493939905#	-5.8354099003#	59.8218943251#
C174	6.9590151493#	-4.8009333292#	59.5000035522#
H175	5.9039259952#	-4.4903550137#	59.5181449656#
H176	7.4904337346	-4.1752909056	60.2313039626
C177	7.4450525833	-4.4337285267	58.0925472480
H178	8.4461103344	-4.8388778950	57.8902712233
H179	7.5293766072	-3.3410157783	58.0355814269
O180	6.5268291270	-4.8190394517	57.0826914182
H181	6.8229371544	-5.7066490851	56.7921615879
H182	12.2133670302	-6.9989314074	57.6076246783
H183	13.8296363735	-8.3168621548	58.2461069531
O184	13.8753190760	-7.3345067684	58.1912800615
H185	11.4686881904	-9.0612052781	57.6183593836
H186	14.4921467640	-7.1456346866	57.4542883185
O187	13.3372986368	-10.0605094575	58.2568842478
H188	14.0903079102	-10.5382223230	58.6404608285
H189	13.4897385005	-10.1175184754	57.2657400056

The N_2H_4 product structure in E_6 without water
Energies: E= -8031,021926 solv = -0.209069, disp = --241,79 Z_0 = 913.17

Mo1	16.4571835288	-5.8044889346	53.2295522217
Fe2	9.0587189795	-7.1309151438	54.8118137807
Fe3	11.4754599597	-5.6141387358	55.4706209295
Fe4	11.0449050340	-6.8515685589	53.1019283779
Fe5	12.0016594535	-8.5799720696	54.9744127937
Fe6	14.5445541482	-7.9237512863	54.3002033023
Fe7	14.1449152451	-5.3964866725	55.0730753674
Fe8	13.3884271765	-5.8445761193	52.5458467831
C9	17.7687558878	-2.2043112288	54.2004397738
H10	17.9326054049	-1.1955390786	53.8161684311
H11	16.6870248807	-2.3325935933	54.3309927359
C12	18.2659912849	-3.2643400496	53.1919200389
C13	19.7365957382	-2.9904837124	52.8045087759
H14	20.3579355593	-3.1467561834	53.6913019207
H15	19.8173431637	-1.9386705237	52.5093281326
C16	20.2313882275	-3.8784257381	51.6428056138
H17	19.7805881281	-3.5700044782	50.6983258253
H18	19.9441780835	-4.9163986583	51.8462925048
C19	17.4764500892	-3.3843411161	51.8512148117
O20	17.1348463512	-4.6034940474	51.5412693349
O21	17.3493611623	-2.3896260052	51.1371880363
O22	18.1677916810	-4.5458055469	53.8424877309
C23	12.6242776155	-6.6777576170	54.2533917616
S24	12.9647029623	-4.0580807978	56.6819241331
S25	16.1066127020	-6.5720812268	55.5675692054
S26	9.6093987701	-5.0021614953	54.0609066996
S27	13.8202977767	-9.8768194612	55.3667905357

S28	14.6811020753	-3.9710695734	53.2437544617
S29	9.9451915569	-8.9495739596	53.5984092290
S30	15.0610978849	-7.4880107670	52.0176203218
S31	11.7273264249	-6.2994047120	50.7242096711
H32	13.6351742266#	-2.1902104904#	49.0425559871#
C33	13.2979243721#	-2.0241175025#	50.0550234852#
H34	13.8728573548	-2.6930209718	50.7008722559
H35	13.4997129112	-0.9880054787	50.3492323177
N36	11.8658818841	-2.3325907876	50.1399226466
H37	11.2775631572	-1.9845999495	49.3961424767
C38	11.2143919939	-2.7934467295	51.2231551743
N39	11.8049054026	-3.0364566102	52.3748593640
H40	11.2799980786	-3.5738660235	53.0758525080
H41	12.8278992699	-3.0398282797	52.5156004851
N42	9.8574587331	-2.9556222947	51.1410255794
H43	9.4870424793	-3.2099897519	50.2341935144
H44	9.4433849612	-3.4827430155	51.9156163695
H45	7.9301611064	0.3873688835	59.4126925740
C46	8.3372603365	-1.3515262729	60.6921734158
H47	8.2853917971	-1.5590434252	61.7677840597
H48	7.5153364501	-1.9049584456	60.2215568744
C49	9.6621321761	-1.8433764676	60.2053080313
N50	10.7299970308	-1.9753770833	61.0726553235
C51	10.0561970777	-2.1469726920	58.9222432166
H52	9.5263480506	-2.1805195656	57.9833033278
C53	11.7446714741	-2.3554974593	60.3178219814
H54	12.7465122782	-2.5685653751	60.6635419019
N55	11.3917838703	-2.4609938055	59.0089367500
H56	19.2187254422	-4.4003174052	57.4485505827
H57	16.7290997161	-8.8385348171	50.7963327688
C58	18.4038018772	-7.4330483261	50.6196067322
H59	17.7991881704	-6.5237761304	50.5717188254
H60	19.2416384870	-7.2933492877	49.9218055011
C61	18.9572276737	-7.5722159992	52.0031694524
N62	18.2239594738	-7.2213755207	53.1268373576
C63	20.1785655096	-8.0471479919	52.4153090341
H64	21.0272677532	-8.4169630028	51.8607790004
C65	18.9774664748	-7.4758290246	54.1849593088
H66	18.7170360964	-7.2736285607	55.2123114121
N67	20.1664994818	-7.9881336917	53.7995043992
H68	20.9295881643	-8.1770566891	54.4322926613
H69	6.9715674059	-10.5409196546	56.9199162381
C70	6.1750546478#	-9.8249632719#	56.6900008256#
H71	5.5309554076#	-10.2322281456#	56.0424860716#
H72	5.6932325146#	-9.5371412375#	57.6127204976#
S73	8.0437502290	-7.7134618995	56.8682888847
H74	11.9671292425	-2.7900859412	58.2318111562
H75	18.3684389284	-4.3488311230	54.8368242944
C76	18.4059939945	-2.3127134057	55.6161730504
O77	18.7207443513	-3.5017480991	56.0203652827

O78	18.5322208426	-1.2685925808	56.2824502312
C79	21.7217721403	-3.8347654515	51.4971482799
O80	22.3758209214	-3.4823476618	50.5346101506
O81	22.3208170453#	-4.2658019772#	52.6398596054#
H82	15.7024655274#	-0.1996319908#	61.4912314330#
C83	14.8549938258#	-0.0829907223#	60.8320019011#
H84	13.9165015893#	-0.2867338795#	61.3260937710#
H85	14.8257084877	0.9912018562	60.5831034254
C86	15.0599853887#	-0.8751945629#	59.5441990368#
H87	14.3514547835	-0.5307419748	58.7805104212
H88	14.8397842310	-1.9348440964	59.7175033155
C89	16.4885332513	-0.7777782712	58.9966957947
H90	16.8714560560	0.2493316979	59.0936965738
H91	16.5228358926	-0.9990210285	57.9240756545
C92	17.4643053142	-1.7248458909	59.7076151223
O93	17.1859765382	-2.2898464701	60.7734370192
N94	18.6597721843	-1.8752377612	59.0821012263
H95	19.2135790758	-2.6611823380	59.4025134954
H96	18.7381542144	-1.6391127093	58.0802040622
C97	10.6519927128#	0.0100028760#	53.3460013492#
H98	11.1636988578#	0.9592721269#	53.2872698117#
H99	9.5863320692#	0.1850134571#	53.3579374457#
H100	10.9228870519	-0.5862180203	52.4741610769
C101	11.0823585132	-0.6314365786	54.6798016330
H102	10.7417401196	-1.6752948620	54.7045958829
C103	10.4090785708	0.1100184274	55.8446042342
H104	9.3151767233	0.0385252753	55.7854057625
H105	10.6750872370	1.1768292680	55.8319808001
H106	10.7198210333	-0.2958366464	56.8126264734
C107	12.6082766995	-0.6088828462	54.8414528995
H108	13.1205794623	-1.1189680876	54.0193262153
H109	12.9187755186	-1.1067139709	55.7653991379
H110	12.9742317145	0.4275271857	54.8738483969
H111	13.4550780017#	-12.2303882915#	53.1883141596#
C112	13.9110015086#	-11.6210557495#	52.4219865947#
H113	14.7493647886	-11.0695652102	52.8518091224
H114	14.2070554607	-12.2140916556	51.5520589185
N115	12.7989755741	-10.7322033722	52.0567278217
H116	12.1482813055	-10.5151538189	52.8135079987
C117	12.6601950676	-10.0200205504	50.9485466917
N118	13.7146025090	-9.8701807873	50.0997139368
H119	13.5174673727	-9.2653590865	49.3097180546
H120	14.5609929851	-9.5577442573	50.5839722248
N121	11.4481874780	-9.5234732515	50.6191287406
H122	10.7424858611	-9.5772080757	51.3638685917
H123	11.4505562885	-8.5854237947	50.2087555855
H124	19.1837820233	-8.2113641757	61.7189338375
C125	19.5978935964#	-7.2298791783#	61.9900504864#
H126	20.6415191229	-7.3774575401	62.2867862145
C127	18.8496279650	-6.6533947730	63.1923737682

O128	19.4089542598	-6.0940362625	64.1317964266
C129	19.4918714603#	-6.2717985978#	60.7990632384#
H130	19.9105961883	-5.2974889412	61.0761991305
H131	18.4369952692	-6.0869795279	60.5711002298
C132	20.1815459537	-6.7978784484	59.5260558582
H133	19.8796652272	-7.8346770700	59.3313499344
H134	21.2714927176	-6.8337840920	59.6688005798
C135	19.9434381845	-6.0587427362	58.1983749580
O136	20.2501492849	-6.5710671072	57.1330552728
O137	19.4118444345	-4.8453261732	58.3339936605
N138	17.4934239852	-6.7874902262	63.0875120872
H139	17.1338832193	-7.1773978915	62.2253587761
C140	16.5368852431#	-6.0058845498#	63.8530406976#
H141	15.8737307499	-6.6852848119	64.4073654985
H142	17.1062936927	-5.4284767680	64.5852821488
C143	15.6905527994	-5.0754426305	62.9436663800
H144	16.2881909049	-4.2060958528	62.6503259870
H145	14.8439072066	-4.7019563245	63.5364693645
C146	15.2029978723	-5.7763881972	61.6848589129
C147	14.3489567993	-6.8917156318	61.7434151648
H148	13.9500005074	-7.2164200914	62.7032489202
C149	15.6868404142	-5.3704014591	60.4298324990
H150	16.3320918179	-4.4967492393	60.3709211831
C151	14.0238005239	-7.6057723407	60.5862856874
H152	13.3843695268	-8.4832236167	60.6580011192
C153	15.3609513682	-6.0813355750	59.2689500243
H154	15.7648749277	-5.7788731571	58.3064373713
C155	14.5438556016	-7.2144280997	59.3466803625
H156	14.3592483857	-7.8050649516	58.4513467654
H157	9.0037559933#	0.7050734487#	60.7752692828#
C158	8.1269804566#	0.1560799507#	60.4649994646#
H159	7.2836720549#	0.4592420318#	61.0677700661#
H160	11.0365558824	-6.6802591767	58.2407690293
H161	18.2592785184#	-9.4987525833#	50.1505400648#
C162	17.5839749123#	-8.6559204782#	50.1520271034#
H163	17.2773627023#	-8.4419890655#	49.1387987742#
H164	23.2793807805	-4.1931628483	52.4753239759
H165	10.7359524977	-5.3855005319	50.6533459420
H166	10.2202645537	-8.4537191955	57.0305216450
H167	13.3123270169	-4.7347216931	57.8036992150
C168	6.7302558801	-8.6157922975	55.9479469096
H169	7.1330468156	-8.9564140245	54.9862145708
H170	5.9141487269	-7.9152349770	55.7323497037
N171	11.2402826250	-8.3248457590	57.0209581808
N172	11.5070876255	-6.9417722065	57.3692043921
H173	7.1493940303#	-5.8354099090#	59.8218942743#
C174	6.9590151221#	-4.8009332891#	59.5000036303#
H175	5.9039259956#	-4.4903550125#	59.5181449576#
H176	7.4995434115	-4.1661494978	60.2158132317
C177	7.4299526525	-4.4666013624	58.0798887651

H178	8.4548774520	-4.8192792238	57.8995314379
H179	7.4454789182	-3.3746289528	57.9713023020
O180	6.5440846058	-4.9586525670	57.0877105601
H181	6.8904696674	-5.8486100945	56.8654689045
H182	12.5105727287	-6.8784903610	57.5397794242
H183	11.6730053934	-8.9691906301	57.6862275946

The N_2H_4 structure in E_7 with two waters
Energies: E= -8184.477879 solv = -0.210156, disp = --247,51 Z_0 = 948.63

Mo1	16.3820543283	-5.7285431603	53.2695754830
Fe2	9.1930817110	-6.9999180794	55.1040231488
Fe3	11.3551757849	-5.3694928335	55.1223674498
Fe4	11.0118770364	-6.9933983856	52.9513784074
Fe5	11.9435919574	-8.3931480987	55.1540660149
Fe6	14.3906315578	-7.6398618610	54.2341651021
Fe7	14.0244451985	-5.1733946981	55.0880781550
Fe8	13.3086037886	-5.7739321496	52.4764342103
C9	17.7883168751	-2.1239205051	54.1957886579
H10	17.9988576357	-1.1309679124	53.7938557407
H11	16.7011960489	-2.2021101807	54.3209149589
C12	18.2482303604	-3.2242051581	53.2154776691
C13	19.7295003669	-3.0158262294	52.8262560488
H14	20.3442280450	-3.1759954008	53.7169365425
H15	19.8481429254	-1.9731175707	52.5115138341
C16	20.1954296639	-3.9408620646	51.6830384926
H17	19.7254690004	-3.6617859456	50.7385342360
H18	19.9102689776	-4.9714779135	51.9226184500
C19	17.4544719197	-3.3412696707	51.8818306029
O20	17.0782457417	-4.5601497192	51.5963006187
O21	17.3410201082	-2.3614748938	51.1488084628
O22	18.0953576620	-4.4850342915	53.8949759033
C23	12.6352820238	-6.6015408804	54.1857326278
S24	12.6476131110	-4.1126715344	56.7658772026
S25	15.9691580235	-6.4538462600	55.5683128238
S26	9.4754192611	-5.0967252295	53.6341533137
S27	13.9274444521	-9.7033410940	55.0380624423
S28	14.6060766040	-3.8870622024	53.2549708842
S29	9.9916412699	-8.9644569701	53.7038970723
S30	15.0592195013	-7.3314067198	51.9833933169
S31	11.7499224030	-6.2611752574	50.6214697797
H32	13.6351744723#	-2.1902108297#	49.0425561244#
C33	13.2979238940#	-2.0241171785#	50.0550234725#
H34	13.8153979513	-2.7415558071	50.6956681646
H35	13.5567083587	-1.0090642058	50.3750935729
N36	11.8474196871	-2.2477471592	50.0908494273
H37	11.3019388278	-1.8636013371	49.3323847730
C38	11.1412496938	-2.7556627531	51.1162072852
N39	11.6757824607	-3.0497032885	52.2842128896

H40	11.1101259887	-3.6246790396	52.9301501687
H41	12.6764748871	-2.9784116746	52.4904621338
N42	9.7958050406	-2.9312537587	50.9616266756
H43	9.4736993611	-3.1631327596	50.0306416708
H44	9.3530148561	-3.4864101272	51.7055098619
H45	7.9326469084	0.3898979861	59.4124976494
C46	8.3469859731	-1.3539847073	60.6925791266
H47	8.1677106558	-1.5765537668	61.7514539809
H48	7.6006425416	-1.9188323500	60.1218485838
C49	9.7333790471	-1.8234881457	60.3748536614
N50	10.7010603875	-1.8663887462	61.3622151848
C51	10.2716253020	-2.2177817903	59.1709676864
H52	9.8479498675	-2.3505503536	58.1879847568
C53	11.7990002513	-2.2798387956	60.7566469734
H54	12.7577386040	-2.4523173450	61.2259697160
N55	11.5943980366	-2.4974880453	59.4307783117
H56	19.2142567716	-4.3325751768	57.5039256294
H57	16.7239509038	-8.8638754929	50.7845947179
C58	18.3672912186	-7.4217950864	50.6550746544
H59	17.7523890763	-6.5208211016	50.5847231039
H60	19.2266077236	-7.2676372345	49.9874640327
C61	18.8782399763	-7.5473490898	52.0599272608
N62	18.1383960073	-7.1522196680	53.1693986889
C63	20.0827613883	-8.0385488698	52.5021965518
H64	20.9307001864	-8.4379638023	51.9674216688
C65	18.8747709465	-7.4008052354	54.2422089656
H66	18.6136535974	-7.1698114274	55.2630493029
N67	20.0552532857	-7.9467822490	53.8834772520
H68	20.8059616976	-8.1347929747	54.5317993327
H69	6.9566279640	-10.5557212562	56.9294189568
C70	6.1750554069#	-9.8249629540#	56.6900013053#
H71	5.5309554802#	-10.2322278715#	56.0424858272#
H72	5.6932323192#	-9.5371413423#	57.6127204285#
S73	8.0925332727	-7.8218038145	56.9951101098
H74	12.2609082762	-2.8423556369	58.7462663555
H75	18.2912020816	-4.2661265296	54.8952737671
C76	18.4113371697	-2.2349305552	55.6171749719
O77	18.6646342033	-3.4319462980	56.0427871416
O78	18.5812172351	-1.1880897741	56.2651470402
C79	21.6833396091	-3.9096348938	51.4924790499
O80	22.3039145568	-3.6274232671	50.4863241478
O81	22.3208168638#	-4.2658019448#	52.6398596022#
H82	15.7024655395#	-0.1996319687#	61.4912314212#
C83	14.8549939674#	-0.0829907232#	60.8320019243#
H84	13.9165015893#	-0.2867338795#	61.3260937710#
H85	14.8218460963	0.9908942824	60.5805695040
C86	15.0599852768#	-0.8751946136#	59.5441989747#
H87	14.3286123785	-0.5579479698	58.7900365432
H88	14.8816993333	-1.9423354195	59.7249824500
C89	16.4764535998	-0.7174203339	58.9749649531

H90	16.7828146690	0.3394241108	59.0047515990
H91	16.5152214278	-1.0013966465	57.9173527545
C92	17.5303374430	-1.5385523290	59.7321901177
O93	17.3301556787	-2.0173946977	60.8541236189
N94	18.7133319893	-1.6718280090	59.0775351983
H95	19.3378727085	-2.3720931670	59.4585660592
H96	18.7614599829	-1.5034628008	58.0619753216
C97	10.6519929066#	0.0100026708#	53.3460012827#
H98	11.1636987957#	0.9592721627#	53.2872698476#
H99	9.5863320799#	0.1850135230#	53.3579374384#
H100	10.9190410583	-0.5795451598	52.4673262767
C101	11.0729280314	-0.6600187645	54.6674628341
H102	10.7339245523	-1.7047767545	54.6569629664
C103	10.3901002521	0.0413789804	55.8515929835
H104	9.2970211538	-0.0265600826	55.7788663132
H105	10.6588434042	1.1070596948	55.8819921937
H106	10.6900273458	-0.4025793052	56.8065581445
C107	12.5967734585	-0.6429875299	54.8390892440
H108	13.1098833601	-1.1204334730	53.9981806603
H109	12.9010643312	-1.1745358204	55.7468489464
H110	12.9651265706	0.3902771811	54.9125829039
H111	13.4550782118#	-12.2303881005#	53.1883144366#
C112	13.9110011658#	-11.6210558695#	52.4219861717#
H113	14.7799780306	-11.1013988231	52.8271150559
H114	14.1609082442	-12.2185785638	51.5395191841
N115	12.8165425251	-10.7038099350	52.0897764089
H116	12.1821421694	-10.4579357311	52.8547241667
C117	12.6470217671	-10.0321049841	50.9628944536
N118	13.6785448104	-9.9061161531	50.0797519925
H119	13.4652922223	-9.3244416626	49.2769549054
H120	14.5484603201	-9.6117107794	50.5250975699
N121	11.4248001841	-9.5514750985	50.6478290491
H122	10.7329872317	-9.6065467085	51.4083062588
H123	11.4054344822	-8.6276689154	50.2066421457
H124	19.2163243616	-8.2196641450	61.6999217289
C125	19.5978939533#	-7.2298787978#	61.9900509001#
H126	20.6387653042	-7.3574082870	62.3055546710
C127	18.8175929349	-6.7214857264	63.1998625972
O128	19.3546100509	-6.3659961467	64.2474526731
C129	19.4918709268#	-6.2717989826#	60.7990629592#
H130	19.8867028764	-5.2894052239	61.0836283184
H131	18.4386354219	-6.1000747192	60.5549673088
C132	20.1968876058	-6.7646881208	59.5205397346
H133	19.9230475875	-7.8057605194	59.3093279359
H134	21.2879921211	-6.7702308106	59.6595926901
C135	19.9286927105	-6.0053838086	58.2098198547
O136	20.1947032235	-6.5014339862	57.1249612351
O137	19.4190627081	-4.7856985508	58.3781206327
N138	17.4716144396	-6.6941988709	62.9896037366
H139	17.1259124331	-6.9284646380	62.0672858694

C140	16.5368850635#	-6.0058847801#	63.8530402503#
H141	15.7954529388	-6.7199899020	64.2393319077
H142	17.1097234363	-5.6269608271	64.7034611003
C143	15.8061987985	-4.8590323378	63.1184413825
H144	16.5157832413	-4.0534602577	62.9003435740
H145	15.0444307921	-4.4531850237	63.7982934487
C146	15.1827082731	-5.3458960559	61.8277031549
C147	14.0078289532	-6.1143283413	61.8306144785
H148	13.4750818672	-6.2788640838	62.7664862062
C149	15.8417835360	-5.1236969730	60.6073169403
H150	16.7225680163	-4.4863834928	60.5894690818
C151	13.5168334165	-6.6669495503	60.6467744852
H152	12.6025415995	-7.2534282307	60.6473016490
C153	15.3573476395	-5.6869061820	59.4226024189
H154	15.8774577903	-5.5179483622	58.4837502167
C155	14.2038405216	-6.4743632654	59.4449680451
H156	13.8326151152	-6.9390420934	58.5369248466
H157	9.0037560028#	0.7050734267#	60.7752692956#
C158	8.1269805061#	0.1560798500#	60.4649992204#
H159	7.2836720924#	0.4592419776#	61.0677701458#
H160	12.9498520296	-9.0932744051	58.1131818785
H161	18.2592787434#	-9.4987524028#	50.1505399005#
C162	17.5839747942#	-8.6559205505#	50.1520276929#
H163	17.2773626050#	-8.4419890969#	49.1387987971#
H164	23.2735811131	-4.2118429823	52.4383225559
H165	10.2848571407	-8.8723252173	57.1258387130
H166	12.5185430105	-5.1259558033	57.6492612196
H167	7.1953946384	-8.9320900853	55.0293781087
C168	6.7749900675	-8.6174446194	55.9922460897
H169	5.9892231065	-7.8802181106	55.7832613991
H170	8.5128207966	-4.8192555437	57.9719697474
N171	11.2459502744	-9.2243393705	57.0451314941
N172	11.9600410281	-8.8552128208	58.2465902413
H173	7.1493939960#	-5.8354098649#	59.8218944361#
C174	6.9590151241#	-4.8009334723#	59.5000035165#
H175	5.9039260039#	-4.4903549799#	59.5181448735#
H176	7.4927928397	-4.1711347277	60.2264154857
C177	7.4758207088	-4.4778415340	58.0951881086
H178	7.4747934194	-3.3877786531	57.9620999910
H179	6.9667313343	-5.9180414256	56.9361707891
O180	6.6447063766	-5.0022154444	57.0721015293
H181	11.1925563776	-10.2427663275	56.9302517292
H182	10.5727453678	-6.5505092459	56.1515364723
H183	10.7569010369	-5.3483658851	50.6028127504
H184	11.6351567641	-9.4440056032	59.0170020920
O185	14.8553081708	-9.7930906349	58.1818324311
H186	14.4504442630	-10.6622386424	57.9813761562
H187	14.9778615918	-9.4171204648	57.2878651061
O188	13.2918214695	-12.0041132351	57.1287858057
H189	13.9200281337	-12.6880489980	56.8494041196

H190	13.3411185431	-11.3456608926	56.3902748264

The N_2H_4 structure in E_8 with two waters

Energies: E= -8185.060093 solv = -0.207658, disp = --253,89 Z_0 = 955.07

Mo1	16.4319963208	-5.7423797253	53.2007044983
Fe2	9.2424432076	-6.9170590109	55.1549658998
Fe3	11.4432663303	-5.3120157134	55.2702112536
Fe4	10.9888676230	-6.7996475258	53.0311011304
Fe5	11.9584160090	-8.2986930886	55.1049660287
Fe6	14.4821953868	-7.7950603533	54.0198650024
Fe7	14.0829405266	-5.4066081487	55.0747568600
Fe8	13.3396673144	-5.7359525006	52.4677583817
C9	17.7670349166	-2.1488950944	54.2386885891
H10	17.9533677605	-1.1448831614	53.8530274837
H11	16.6820366923	-2.2587061545	54.3600757030
C12	18.2547900978	-3.2197285915	53.2384562206
C13	19.7370020996	-2.9804939247	52.8674423467
H14	20.3478667710	-3.1769416127	53.7536011964
H15	19.8503217537	-1.9238204200	52.6014296403
C16	20.2161456615	-3.8482665646	51.6839756276
H17	19.7568114640	-3.5184282692	50.7510014535
H18	19.9282065147	-4.8896858795	51.8673989709
C19	17.4791663139	-3.3130405662	51.8904066745
O20	17.1294167966	-4.5274091404	51.5623975478
O21	17.3703455554	-2.3125985450	51.1841095041
O22	18.1168689890	-4.5014448452	53.8863670823
C23	12.6215082853	-6.5835689950	54.2004394378
S24	12.9559066293	-3.9885168788	56.6471517722
S25	16.0272739867	-6.6446396074	55.5081561685
S26	9.5026717282	-4.9481257499	53.8220576932
S27	13.8298230679	-9.8913075197	55.0677401020
S28	14.6523484636	-3.9230185841	53.2741536904
S29	9.9909872874	-8.8633541338	53.7371685953
S30	15.0516447728	-7.2978570914	51.8216222582
S31	11.6948595425	-6.2491754738	50.6293404109
H32	13.6351744687#	-2.1902108066#	49.0425561195#
C33	13.2979239164#	-2.0241171950#	50.0550234606#
H34	13.8404183206	-2.7214435718	50.6981825751
H35	13.5319631868	-0.9996495348	50.3653466191
N36	11.8566373955	-2.2869266669	50.1073938702
H37	11.2930005127	-1.9176892911	49.3549739818
C38	11.1756987110	-2.7597288435	51.1659967050
N39	11.7413370627	-3.0290383880	52.3250051694
H40	11.1826971089	-3.5678071450	53.0035075985
H41	12.7542940959	-2.9913039484	52.5006697587
N42	9.8237031864	-2.9189671581	51.0539808448
H43	9.4705665273	-3.1517995496	50.1346016143
H44	9.3968279838	-3.4628971172	51.8152517245

H45	7.9308125670	0.3842792417	59.4120368980
C46	8.3400181678	-1.3505946988	60.6983703833
H47	8.2892975945	-1.5543160344	61.7746925581
H48	7.5193624599	-1.9078806340	60.2300843537
C49	9.6666058031	-1.8381612605	60.2124319816
N50	10.7277541152	-1.9965737872	61.0836646715
C51	10.0692857837	-2.1058930882	58.9244804771
H52	9.5444238838	-2.1175196866	57.9822059592
C53	11.7475380898	-2.3576079698	60.3261876897
H54	12.7450235101	-2.5872183787	60.6735229499
N55	11.4033166758	-2.4252666905	59.0126184681
H56	19.1474860305	-4.3399964036	57.5094896543
H57	16.7275357966	-8.8420966593	50.7933273242
C58	18.3980155515	-7.4270455767	50.6128633125
H59	17.7896087728	-6.5209535810	50.5507981886
H60	19.2382697480	-7.2929113227	49.9172482315
C61	18.9423658746	-7.5480704966	52.0002412538
N62	18.2048551180	-7.1721097616	53.1125247339
C63	20.1572368330	-8.0272059103	52.4261338003
H64	21.0054106281	-8.4138975527	51.8824780669
C65	18.9516606942	-7.4156872977	54.1786857209
H66	18.6907058572	-7.1961299599	55.2020132834
N67	20.1380273679	-7.9439380293	53.8084266350
H68	20.8937813611	-8.1345321851	54.4496802227
H69	6.9535351017	-10.5591001581	56.9297987645
C70	6.1750552586#	-9.8249630233#	56.6900012275#
H71	5.5309554680#	-10.2322279174#	56.0424858682#
H72	5.6932323562#	-9.5371413176#	57.6127204401#
S73	8.0905114958	-7.8341797549	57.0013396002
H74	11.9849920959	-2.7193657726	58.2279925654
H75	18.2958154057	-4.3104629670	54.8887860856
C76	18.3929743341	-2.2700020925	55.6558238943
O77	18.6331083432	-3.4711219105	56.0763604017
O78	18.5818047836	-1.2263091072	56.3065104947
C79	21.7050415972	-3.8049620570	51.5183395019
O80	22.3442006017	-3.4311185108	50.5542514230
O81	22.3208168937#	-4.2658019519#	52.6398596037#
H82	15.7024655397#	-0.1996319682#	61.4912314210#
C83	14.8549939542#	-0.0829907267#	60.8320019243#
H84	13.9165015893#	-0.2867338795#	61.3260937710#
H85	14.8233085318	0.9908845166	60.5811441464
C86	15.0599852820#	-0.8751946070#	59.5441989786#
H87	14.3385091502	-0.5487338317	58.7849911137
H88	14.8674626088	-1.9399616708	59.7203923290
C89	16.4831067989	-0.7378692448	58.9866092473
H90	16.8144173026	0.3106049674	59.0338039359
H91	16.5236761306	-1.0060271169	57.9249809689
C92	17.5126304445	-1.5975560835	59.7340636421
O93	17.2785365183	-2.1202321799	60.8309434618
N94	18.7092061575	-1.7199815082	59.1030799276

H95	19.3098755204	-2.4498709425	59.4672573541
H96	18.7730276336	-1.5304165754	58.0902292866
C97	10.6519928842#	0.0100026886#	53.3460012892#
H98	11.1636988006#	0.9592721601#	53.2872698489#
H99	9.5863320789#	0.1850135165#	53.3579374372#
H100	10.9230990600	-0.5872762011	52.4758907628
C101	11.0847518567	-0.6257570478	54.6814611840
H102	10.7435323250	-1.6693331393	54.7134684973
C103	10.4165910208	0.1239907171	55.8437956222
H104	9.3224728271	0.0538552151	55.7883109818
H105	10.6843257486	1.1903203276	55.8234498762
H106	10.7294595182	-0.2758392777	56.8135937210
C107	12.6111621082	-0.6022072148	54.8361850029
H108	13.1196369123	-1.1340016843	54.0251133080
H109	12.9243134513	-1.0771241385	55.7711230839
H110	12.9792403208	0.4339483378	54.8416757831
H111	13.4550782648#	-12.2303881279#	53.1883144464#
C112	13.9110010384#	-11.6210558494#	52.4219861691#
H113	14.7911568787	-11.1135143856	52.8161842071
H114	14.1439374682	-12.2090464759	51.5289227439
N115	12.8089562427	-10.6893701074	52.1201073487
H116	12.1321895812	-10.5254536051	52.8665835019
C117	12.6154109819	-9.9731183858	51.0200619219
N118	13.6312254549	-9.8161635526	50.1251950167
H119	13.4055446099	-9.1868191399	49.3620015072
H120	14.5048638278	-9.5264760398	50.5704668533
N121	11.3902307723	-9.4828196382	50.7526400142
H122	10.7216295146	-9.5206387730	51.5364559457
H123	11.3646785599	-8.5586831846	50.3110056248
H124	19.2129408770	-8.2196168536	61.7042573756
C125	19.5978938892#	-7.2298788799#	61.9900508205#
H126	20.6386842718	-7.3585489289	62.3041916131
C127	18.8214014144	-6.7084968096	63.1961677831
O128	19.3600314810	-6.3003276245	64.2218481009
C129	19.4918710336#	-6.2717989020#	60.7990630222#
H130	19.8942428878	-5.2916369918	61.0810525351
H131	18.4372712427	-6.0938091028	60.5657130362
C132	20.1807845764	-6.7671651012	59.5141394946
H133	19.8992964231	-7.8064706305	59.3038111089
H134	21.2731056532	-6.7786205085	59.6407151860
C135	19.9009944253	-6.0011591949	58.2085182194
O136	20.1910172173	-6.4786741413	57.1225994637
O137	19.3501247993	-4.8014364726	58.3850011589
N138	17.4670925351	-6.7315293900	63.0110518628
H139	17.1246024944	-6.9809033446	62.0920167782
C140	16.5368850898#	-6.0058847199#	63.8530403706#
H141	15.7695459338	-6.6969855950	64.2295810178
H142	17.1083309314	-5.6425142069	64.7107781528
C143	15.8540537777	-4.8371470731	63.1060310178
H144	16.5915760667	-4.0607625704	62.8768528943

H145	15.1090632203	-4.3933292548	63.7803717520
C146	15.1988232532	-5.3053302912	61.8229510483
C147	14.0239028639	-6.0717814954	61.8493216132
H148	13.5272945336	-6.2649705657	62.7985683662
C149	15.8059214743	-5.0419348252	60.5834853363
H150	16.6802786636	-4.3986421556	60.5486799635
C151	13.4762472213	-6.5755885810	60.6674155648
H152	12.5487432283	-7.1425809466	60.7011805191
C153	15.2696990030	-5.5567745384	59.3997977190
H154	15.7430581291	-5.3490145512	58.4440888839
C155	14.1093228372	-6.3336866980	59.4418263208
H156	13.7029672542	-6.7390287488	58.5211270301
H157	9.0037559992#	0.7050734323#	60.7752692959#
C158	8.1269805209#	0.1560799221#	60.4649992508#
H159	7.2836720866#	0.4592419632#	61.0677701449#
H160	11.3006727639	-7.4239340260	57.8997432752
H161	18.2592787657#	-9.4987523851#	50.1505399050#
C162	17.5839749178#	-8.6559205669#	50.1520275804#
H163	17.2773625510#	-8.4419891057#	49.1387988115#
H164	23.2769609770	-4.1910266890	52.4622881554
H165	10.1008228321	-9.1961514386	56.7671542576
H166	12.8939170501	-4.7847090706	57.7364275335
C167	6.7786950405	-8.6203730097	55.9938373990
H168	7.2074269217	-8.9272571897	55.0330667532
H169	5.9958609793	-7.8799543679	55.7857330170
N170	11.1104675996	-9.1461537841	56.9651576060
N171	11.3204810031	-8.4059803395	58.1912934699
H172	7.1493940210#	-5.8354098639#	59.8218944249#
C173	6.9590151079#	-4.8009335040#	59.5000035038#
H174	5.9039260045#	-4.4903549781#	59.5181448806#
H175	7.4967515072	-4.1653716260	60.2176931828
C176	7.4582759889	-4.4869693551	58.0862274651
H177	8.4978985603	-4.8139597794	57.9492386861
H178	7.4412098915	-3.3989023945	57.9435679506
O179	6.6199905523	-5.0366673986	57.0818019097
H180	6.9666596259	-5.9423555182	56.9349159456
H181	14.6142135788	-9.7370911290	56.1964656753
H182	11.4878917404	-10.0848636340	57.0880377523
H183	10.5475381777	-6.4044177547	56.3325765162
H184	10.6902872697	-5.3496993343	50.5647816612
H185	10.4906364651	-8.5241046006	58.7790438561
O186	13.6765501396	-9.5204058755	59.3496490236
H187	12.8813519409	-9.0882118772	58.9391456312
H188	13.9478035402	-8.8885563913	60.0339789802
O189	15.7727893415	-9.4689007548	57.5237265922
H190	15.0616713916	-9.4284420200	58.2079884635
H191	15.9244319840	-8.5538994537	57.2215305188

The N_2H_4 TS structure for proton transfer in E_8 with two waters in **Figure 7**

Energies: E= -8185.041365 solv = -0.205744, disp = --256,44 Z_0 = 953.67

Mo1	16.3465020000	-5.8223330000	53.2747170000
Fe2	9.0886990000	-6.7633900000	55.1362750000
Fe3	11.3044070000	-5.2564430000	55.1632220000
Fe4	10.8673590000	-6.8226820000	52.9956200000
Fe5	11.7570070000	-8.3963290000	54.9968410000
Fe6	14.3118240000	-7.9309270000	54.1396810000
Fe7	13.9456000000	-5.5253780000	55.1764440000
Fe8	13.2731160000	-5.8027590000	52.5275930000
C9	17.7015710000	-2.2097590000	54.2801420000
H10	17.8769910000	-1.2138440000	53.8690590000
H11	16.6183740000	-2.3269810000	54.4130960000
C12	18.1840430000	-3.2985740000	53.3001350000
C13	19.6674150000	-3.0728030000	52.9232500000
H14	20.2790220000	-3.2428820000	53.8150450000
H15	19.7816940000	-2.0248690000	52.6240290000
C16	20.1449680000	-3.9811680000	51.7705360000
H17	19.6409510000	-3.7204090000	50.8384470000
H18	19.9111640000	-5.0228070000	52.0180840000
C19	17.3985800000	-3.3950110000	51.9601030000
O20	17.0361470000	-4.6048200000	51.6345370000
O21	17.2906370000	-2.3935680000	51.2551680000
O22	18.0390890000	-4.5696410000	53.9635770000
C23	12.4671170000	-6.6191330000	54.2140640000
S24	12.7726810000	-3.9706670000	56.6110380000
S25	15.9128370000	-6.7269400000	55.6034460000
S26	9.4074970000	-4.8704220000	53.7124140000
S27	13.5272430000	-9.9269930000	55.1621830000
S28	14.5712710000	-4.0073230000	53.3750220000
S29	9.6781480000	-8.7430220000	53.7200720000
S30	14.9710320000	-7.4311530000	51.9412620000
S31	11.6731270000	-6.2490990000	50.6376850000
H32	13.6351740000	-2.1902110000	49.0425560000
C33	13.2979240000	-2.0241170000	50.0550230000
H34	13.8105300000	-2.7488440000	50.6923930000
H35	13.5622730000	-1.0122450000	50.3812410000
N36	11.8509220000	-2.2433980000	50.0812680000
H37	11.3082480000	-1.8593240000	49.3214250000
C38	11.1479210000	-2.7240230000	51.1206640000
N39	11.6982670000	-3.0098560000	52.2827850000
H40	11.1235530000	-3.5472900000	52.9493080000
H41	12.7089170000	-2.9973150000	52.4683400000
N42	9.7963190000	-2.8631290000	50.9867610000
H43	9.4586900000	-3.0940110000	50.0610310000
H44	9.3532040000	-3.4087300000	51.7385140000
H45	7.9311380000	0.3794060000	59.4109640000
C46	8.3589040000	-1.3494930000	60.7063400000
H47	8.2696310000	-1.5541090000	61.7800270000

H48	7.5697580000	-1.9234130000	60.2059540000
C49	9.7173830000	-1.8050210000	60.2756180000
N50	10.7516830000	-1.9169580000	61.1864920000
C51	10.1778330000	-2.0659550000	59.0052300000
H52	9.6896440000	-2.1077740000	58.0437350000
C53	11.8135220000	-2.2403630000	60.4705090000
H54	12.8048360000	-2.4296920000	60.8567790000
N55	11.5200560000	-2.3306390000	59.1450220000
H56	19.1348280000	-4.3306250000	57.5325230000
H57	16.7195850000	-8.8553830000	50.7821490000
C58	18.3781790000	-7.4304680000	50.6529850000
H59	17.7624660000	-6.5285580000	50.5987810000
H60	19.2326610000	-7.2734310000	49.9797490000
C61	18.8973030000	-7.5724970000	52.0507620000
N62	18.1313760000	-7.2419500000	53.1598880000
C63	20.1204720000	-8.0213860000	52.4879610000
H64	20.9893970000	-8.3688800000	51.9501720000
C65	18.8710210000	-7.4795750000	54.2312370000
H66	18.5861850000	-7.2815040000	55.2525350000
N67	20.0793600000	-7.9650460000	53.8711340000
H68	20.8373580000	-8.1222750000	54.5185060000
H69	6.9793640000	-10.5343770000	56.9152430000
C70	6.1750550000	-9.8249630000	56.6900010000
H71	5.5309550000	-10.2322280000	56.0424860000
H72	5.6932320000	-9.5371410000	57.6127200000
S73	7.9247310000	-7.6788130000	57.0014760000
H74	12.1338340000	-2.5846190000	58.3732040000
H75	18.2112340000	-4.3682040000	54.9586450000
C76	18.3468460000	-2.2993410000	55.6877480000
O77	18.5729040000	-3.4904760000	56.1407220000
O78	18.5660740000	-1.2401090000	56.3029980000
C79	21.6230760000	-3.8766130000	51.5385360000
O80	22.1947720000	-3.5052860000	50.5325590000
O81	22.3208170000	-4.2658020000	52.6398600000
H82	15.7024660000	-0.1996320000	61.4912310000
C83	14.8549940000	-0.0829910000	60.8320020000
H84	13.9165020000	-0.2867340000	61.3260940000
H85	14.8211060000	0.9907010000	60.5804030000
C86	15.0599850000	-0.8751950000	59.5441990000
H87	14.3273670000	-0.5629880000	58.7897980000
H88	14.8902500000	-1.9433670000	59.7251840000
C89	16.4757380000	-0.7080560000	58.9740260000
H90	16.7663950000	0.3534140000	58.9860390000
H91	16.5202340000	-1.0086800000	57.9213900000
C92	17.5429920000	-1.4980050000	59.7452230000
O93	17.3479720000	-1.9640150000	60.8742900000
N94	18.7297650000	-1.6200260000	59.0982390000
H95	19.3704410000	-2.2927260000	59.5004970000
H96	18.7776850000	-1.4792940000	58.0760650000
C97	10.6519930000	0.0100030000	53.3460010000

H98	11.1636990000	0.9592720000	53.2872700000
H99	9.5863320000	0.1850140000	53.3579370000
H100	10.9223070000	-0.5875260000	52.4761150000
C101	11.0986290000	-0.6213130000	54.6791900000
H102	10.7618590000	-1.6655140000	54.7207310000
C103	10.4434430000	0.1324400000	55.8470750000
H104	9.3489590000	0.0556410000	55.8098710000
H105	10.7046020000	1.2002170000	55.8167400000
H106	10.7749880000	-0.2588030000	56.8143130000
C107	12.6276530000	-0.5920480000	54.8142590000
H108	13.1273090000	-1.1210700000	53.9959460000
H109	12.9540820000	-1.0684770000	55.7438670000
H110	12.9924370000	0.4454840000	54.8157300000
H111	13.4550780000	-12.2303880000	53.1883140000
C112	13.9110010000	-11.6210560000	52.4219860000
H113	14.7920310000	-11.1131750000	52.8172020000
H114	14.1405180000	-12.1996800000	51.5228140000
N115	12.8093660000	-10.6927400000	52.1435350000
H116	12.2854850000	-10.4301240000	52.9830420000
C117	12.5634330000	-9.9977630000	51.0452420000
N118	13.5270490000	-9.8713790000	50.0849830000
H119	13.2468380000	-9.2793720000	49.3102700000
H120	14.4131530000	-9.5449540000	50.4786250000
N121	11.3322190000	-9.4929190000	50.8422990000
H122	10.6867400000	-9.5517260000	51.6417180000
H123	11.2873620000	-8.5714150000	50.3989850000
H124	19.2288060000	-8.2240040000	61.6977100000
C125	19.5978940000	-7.2298790000	61.9900510000
H126	20.6370570000	-7.3460250000	62.3134890000
C127	18.8070820000	-6.7288700000	63.1927930000
O128	19.3347590000	-6.3730570000	64.2422420000
C129	19.4918710000	-6.2717990000	60.7990630000
H130	19.8784620000	-5.2868660000	61.0862930000
H131	18.4390130000	-6.1070460000	60.5493850000
C132	20.2041100000	-6.7555170000	59.5255730000
H133	19.9449450000	-7.8001600000	59.3128160000
H134	21.2950340000	-6.7448690000	59.6635870000
C135	19.9172280000	-5.9915580000	58.2214320000
O136	20.2155470000	-6.4702820000	57.1386380000
O137	19.3524830000	-4.8004640000	58.4034040000
N138	17.4546620000	-6.7130860000	62.9850030000
H139	17.1221090000	-6.9078520000	62.0497020000
C140	16.5368850000	-6.0058850000	63.8530400000
H141	15.7138110000	-6.6763170000	64.1363320000
H142	17.0967190000	-5.7607330000	64.7588030000
C143	15.9530750000	-4.7262100000	63.2112910000
H144	16.7546620000	-4.0014940000	63.0342070000
H145	15.2553520000	-4.2774470000	63.9308440000
C146	15.2487990000	-5.0379350000	61.9106230000
C147	13.9753860000	-5.6192290000	61.9057060000

H148	13.4429360000	-5.7587080000	62.8442660000
C149	15.9030950000	-4.8276920000	60.6853440000
H150	16.8551720000	-4.3074930000	60.6771650000
C151	13.3749410000	-6.0031100000	60.7038130000
H152	12.3637370000	-6.4054600000	60.7139640000
C153	15.3195290000	-5.2315120000	59.4840030000
H154	15.8283970000	-5.0727280000	58.5372290000
C155	14.0581810000	-5.8299060000	59.4947810000
H156	13.6181820000	-6.1345090000	58.5524210000
H157	9.0037560000	0.7050730000	60.7752690000
C158	8.1269810000	0.1560800000	60.4649990000
H159	7.2836720000	0.4592420000	61.0677700000
H160	11.0544080000	-7.1977610000	57.6052180000
H161	18.2592790000	-9.4987520000	50.1505400000
C162	17.5839750000	-8.6559210000	50.1520280000
H163	17.2773630000	-8.4419890000	49.1387990000
H164	23.2601750000	-4.1455780000	52.4062130000
H165	9.8277150000	-9.0755010000	56.7928540000
H166	12.4586350000	-4.6896720000	57.7084600000
C167	6.7038790000	-8.5892660000	55.9871710000
H168	7.1713920000	-8.8700680000	55.0380550000
H169	5.8670920000	-7.9164160000	55.7594250000
N170	10.8485790000	-9.0388340000	56.9308010000
N171	11.1035950000	-8.1404560000	58.0328170000
H172	7.1493940000	-5.8354100000	59.8218940000
C173	6.9590150000	-4.8009330000	59.5000030000
H174	5.9039260000	-4.4903550000	59.5181450000
H175	7.4856430000	-4.1749370000	60.2358930000
C176	7.4578590000	-4.4312530000	58.0986510000
H177	8.4678060000	-4.8191740000	57.9105920000
H178	7.5154150000	-3.3377530000	58.0322330000
O179	6.5613160000	-4.8392070000	57.0769780000
H180	6.8032860000	-5.7712780000	56.8925250000
H181	14.7162360000	-9.5845740000	56.8226880000
H182	11.2321690000	-9.9563700000	57.1550290000
H183	10.3365050000	-6.1496010000	56.3636350000
H184	10.6998230000	-5.3170350000	50.5533760000
H185	10.3240500000	-8.1865040000	58.6995440000
O186	13.2610700000	-8.8363740000	59.1313350000
H187	12.2822770000	-8.4555250000	58.5978010000
H188	13.5316240000	-8.1452100000	59.7664830000
O189	15.1728990000	-9.1717770000	57.6284060000
H190	14.1298650000	-8.9487470000	58.4301680000
H191	15.5236340000	-8.3403500000	57.2223780000

The N_2H_5 product structure after proton transfer in E_8 with two waters
Energies: E= -8185.047400 solv = -0.207842, disp = --256,28 Z_0 = 956.52

Mo1	16.3226931935	-5.9101091435	53.3038594443

Fe2	9.0608312579	-6.7306140076	55.1504242820
Fe3	11.3283556847	-5.2850196229	55.2436361015
Fe4	10.8347637062	-6.7794522184	53.0306920006
Fe5	11.6919719647	-8.4485249398	54.9397063008
Fe6	14.2697586006	-8.0076655455	54.2156380594
Fe7	13.9480734127	-5.5884464283	55.2279061583
Fe8	13.2743145828	-5.8570675841	52.5675324138
C9	17.7040101190	-2.2942793112	54.2525147757
H10	17.8854322051	-1.3021802605	53.8348449684
H11	16.6204811820	-2.4008954402	54.3893046050
C12	18.1772631747	-3.3932126161	53.2750987624
C13	19.6580377063	-3.1678814672	52.8865357332
H14	20.2750515889	-3.3134687651	53.7784689377
H15	19.7610940050	-2.1250774645	52.5665201486
C16	20.1413024835	-4.0922146846	51.7502462111
H17	19.6322007710	-3.8576786402	50.8139925649
H18	19.9166685635	-5.1311864062	52.0174145337
C19	17.3794250883	-3.4998671681	51.9409599099
O20	16.9930342654	-4.7087127503	51.6409168298
O21	17.2780566096	-2.5082715649	51.2196543256
O22	18.0344133254	-4.6601269570	53.9499340826
C23	12.4346496278	-6.6540536699	54.2511343904
S24	12.8342877536	-4.0447768626	56.6999330652
S25	15.8980123710	-6.8183600597	55.6308226581
S26	9.4347217496	-4.8106793476	53.8068905704
S27	13.4018560682	-9.9938514297	55.1693904679
S28	14.5684054465	-4.0706182692	53.4238601407
S29	9.5907059360	-8.6918658747	53.7040905972
S30	14.9314885196	-7.5290046068	52.0035439122
S31	11.6502245760	-6.2481028972	50.6801294453
H32	13.6351744528#	-2.1902108168#	49.0425561160#
C33	13.2979239165#	-2.0241171932#	50.0550234964#
H34	13.8260332137	-2.7405241457	50.6892345032
H35	13.5550108724	-1.0084438413	50.3761775999
N36	11.8551791672	-2.2586527525	50.1020096022
H37	11.2962122577	-1.8647822190	49.3588306740
C38	11.1742055601	-2.7190226550	51.1658083204
N39	11.7503704924	-3.0082984536	52.3142300952
H40	11.1843761948	-3.5330174188	52.9963281396
H41	12.7667868498	-3.0282625533	52.4730707454
N42	9.8170396910	-2.8352701407	51.0685644670
H43	9.4485828409	-3.0551359098	50.1519672464
H44	9.3817224447	-3.3697688591	51.8325999579
H45	7.9308748416	0.3896107639	59.4131013316
C46	8.3537560663	-1.3518773661	60.6869493915
H47	8.2598938858	-1.5726544551	61.7568928782
H48	7.5650771784	-1.9168179107	60.1755434317
C49	9.7123388724	-1.8097673883	60.2580059505
N50	10.7279091038	-1.9712572010	61.1825998912
C51	10.1908907613	-2.0381056816	58.9879635724

H52	9.7198405199	-2.0386026660	58.0177460037
C53	11.7950340449	-2.2934969901	60.4747462105
H54	12.7755914711	-2.5173713614	60.8707069908
N55	11.5253691451	-2.3336879610	59.1427312573
H56	19.1487238691	-4.3699045869	57.5011179602
H57	16.7170510720	-8.8599253004	50.7772973780
C58	18.3788137698	-7.4407974925	50.6693591743
H59	17.7672646280	-6.5357783698	50.6286266253
H60	19.2385150235	-7.2762665112	50.0046802016
C61	18.8854798921	-7.6179130464	52.0676282682
N62	18.1039642588	-7.3304824147	53.1778473987
C63	20.1069458975	-8.0713304899	52.5041785075
H64	20.9844946265	-8.3947854897	51.9656885557
C65	18.8320853061	-7.5992261622	54.2496999610
H66	18.5326503969	-7.4353908611	55.2730519931
N67	20.0484435818	-8.0630427499	53.8883643920
H68	20.8010787003	-8.2335195328	54.5389755683
H69	6.9909444161	-10.5226181320	56.9078946223
C70	6.1750553505#	-9.8249629466#	56.6900012068#
H71	5.5309554572#	-10.2322279120#	56.0424858756#
H72	5.6932323518#	-9.5371413123#	57.6127204363#
S73	7.8687899993	-7.6478877147	57.0060592377
H74	12.1543882735	-2.5992357253	58.3869042522
H75	18.2246348011	-4.4524702644	54.9401107157
C76	18.3466428356	-2.3755353181	55.6629382812
O77	18.5921719924	-3.5652555890	56.1109452242
O78	18.5436541409	-1.3154103630	56.2850770446
C79	21.6186735418	-3.9895610950	51.5075106309
O80	22.1866942456	-3.7139599728	50.4683601309
O81	22.3208168301#	-4.2658019349#	52.6398595832#
H82	15.7024655398#	-0.1996319678#	61.4912314208#
C83	14.8549940149#	-0.0829907180#	60.8320019287#
H84	13.9165015893#	-0.2867338795#	61.3260937710#
H85	14.8233957392	0.9908619221	60.5813633710
C86	15.0599852328#	-0.8751946259#	59.5441989576#
H87	14.3438848697	-0.5412941694	58.7831241901
H88	14.8586529830	-1.9388575122	59.7180764272
C89	16.4853526763	-0.7488974456	58.9892028958
H90	16.8260625922	0.2962780859	59.0435204476
H91	16.5240911801	-1.0112298223	57.9261225786
C92	17.5056254625	-1.6228060617	59.7329146138
O93	17.2731415161	-2.1301532709	60.8375617909
N94	18.6916881469	-1.7738127236	59.0889823610
H95	19.2853328116	-2.5103571521	59.4515398488
H96	18.7475760119	-1.5947860284	58.0734602769
C97	10.6519929088#	0.0100026472#	53.3460012824#
H98	11.1636987909#	0.9592721661#	53.2872698615#
H99	9.5863320812#	0.1850135307#	53.3579374302#
H100	10.9232541510	-0.5872463306	52.4766847263
C101	11.0836677048	-0.6252114017	54.6819847344

H102	10.7315195043	-1.6646587322	54.7200112856
C103	10.4276569488	0.1379843666	55.8426042003
H104	9.3326918811	0.0745431781	55.7949113528
H105	10.7023808142	1.2024473101	55.8129583838
H106	10.7449464999	-0.2560206188	56.8132789833
C107	12.6105969642	-0.6180303697	54.8323209475
H108	13.1100237207	-1.1496351524	54.0159529876
H109	12.9208007189	-1.1039792348	55.7626736930
H110	12.9901608036	0.4140926792	54.8440514316
H111	13.4550782819#	-12.2303881093#	53.1883144711#
C112	13.9110010066#	-11.6210558726#	52.4219861339#
H113	14.7803241776	-11.0963812944	52.8216311532
H114	14.1581130726	-12.2019368855	51.5285861123
N115	12.7975842659	-10.7082016868	52.1264150067
H116	12.2805330016	-10.4365990974	52.9692638125
C117	12.5529744097	-10.0135356005	51.0279837224
N118	13.5134959273	-9.8939410229	50.0637535015
H119	13.2363041155	-9.2978928621	49.2909096420
H120	14.4035510507	-9.5771756120	50.4566702029
N121	11.3238542564	-9.4975077298	50.8249713798
H122	10.6708565835	-9.5477775495	51.6171053807
H123	11.2850711053	-8.5757507630	50.3813341570
H124	19.2212294328	-8.2212768963	61.6989923615
C125	19.5978939153#	-7.2298788053#	61.9900508609#
H126	20.6377824378	-7.3526881303	62.3093233451
C127	18.8133215485	-6.7207293468	63.1957825807
O128	19.3458273873	-6.3340704885	64.2328847093
C129	19.4918709150#	-6.2717989652#	60.7990630046#
H130	19.8769060127	-5.2855662571	61.0831271147
H131	18.4387673118	-6.1118047655	60.5485933257
C132	20.2089686871	-6.7670996474	59.5298088457
H133	19.9632299111	-7.8182077736	59.3349694784
H134	21.2996084950	-6.7400291906	59.6688004021
C135	19.9128440856	-6.0295496481	58.2129472989
O136	20.1791524203	-6.5349470330	57.1334456158
O137	19.3788304687	-4.8223300248	58.3795035521
N138	17.4591276051	-6.7296007471	63.0009961563
H139	17.1207450093	-6.9540947040	62.0739699728
C140	16.5368851470#	-6.0058847552#	63.8530403578#
H141	15.7420701167	-6.6854644500	64.1910973637
H142	17.1062382886	-5.6928692018	64.7318267398
C143	15.9018310556	-4.7871633979	63.1443571933
H144	16.6723494430	-4.0424552963	62.9185473801
H145	15.1893246735	-4.3241748668	63.8402517845
C146	15.2046005116	-5.2035527439	61.8682206499
C147	13.9618292872	-5.8503227773	61.9068070195
H148	13.4432049019	-5.9658050853	62.8567525187
C149	15.8386550741	-5.0289910504	60.6259348091
H150	16.7633689379	-4.4622100023	60.5798995404
C151	13.3773135613	-6.3344663813	60.7344190180

H152	12.3907524444	-6.7923990396	60.7814133982
C153	15.2664486236	-5.5276042198	59.4533751330
H154	15.7591620837	-5.3924808600	58.4945660799
C155	14.0396207295	-6.1930381869	59.5094239528
H156	13.6114184033	-6.5813494035	58.5919898293
H157	9.0037560010#	0.7050734218#	60.7752693096#
C158	8.1269805246#	0.1560799089#	60.4649991866#
H159	7.2836720947#	0.4592419437#	61.0677701658#
H160	10.8544482184	-7.2442193836	57.4950954304
H161	18.2592788010#	-9.4987523567#	50.1505398750#
C162	17.5839748858#	-8.6559205865#	50.1520276884#
H163	17.2773625477#	-8.4419890980#	49.1387988141#
H164	23.2589934346	-4.1670805387	52.3920577581
H165	9.6963709977	-9.1939784707	56.6650418669
H166	12.5820119806	-4.7777609265	57.8057687243
C167	6.6702404700	-8.5760306718	55.9879445634
H168	7.1412702898	-8.8366876067	55.0353024872
H169	5.8174216619	-7.9208443671	55.7692842509
N170	10.7076720376	-9.1299762520	56.8487657863
N171	10.8567427825	-8.2018858819	57.9460976959
H172	7.1493940091#	-5.8354098568#	59.8218944545#
C173	6.9590151337#	-4.8009335101#	59.5000034520#
H174	5.9039260015#	-4.4903549887#	59.5181448758#
H175	7.4831081716	-4.1784415637	60.2405489860
C176	7.4581794868	-4.4157575297	58.1024200170
H177	8.4625076542	-4.8119195036	57.9025489679
H178	7.5312900569	-3.3225687927	58.0542342027
O179	6.5509164297	-4.7905070314	57.0763518312
H180	6.7700806977	-5.7239693785	56.8789975307
H181	14.6742260346	-9.7537523702	56.9066879623
H182	11.1169062158	-10.0247459432	57.1198158899
H183	10.3335182323	-6.1950787534	56.4343757375
H184	10.7141339441	-5.2811780009	50.5789159889
H185	10.0356331574	-8.2385029969	58.5656800004
O186	12.9695813492	-9.1884281181	59.2502094652
H187	11.7596852179	-8.4476613139	58.4723620656
H188	13.2906538935	-8.6481220750	59.9907303276
O189	15.1142932138	-9.4074533956	57.7280986564
H190	13.7857502332	-9.2670601221	58.6511471393
H191	15.4705403262	-8.5565571993	57.3986038332

The N-N TS structure for N_2H_5 in E_8 with two waters in **Figure 8**
Energies: E= -8185.030065 solv = -0.203929, disp = --253.55 Z_0 = 954.08

Mo1	16.4228970000	-5.8101520000	53.3339930000
Fe2	9.1771760000	-6.7976330000	55.2467330000
Fe3	11.4230210000	-5.3325780000	55.3010160000

Fe4	10.9778970000	-6.8533200000	53.0957810000
Fe5	11.8919620000	-8.4244090000	55.1398410000
Fe6	14.4704720000	-7.9489660000	54.2038000000
Fe7	14.0716460000	-5.5915850000	55.3220590000
Fe8	13.3745540000	-5.8415530000	52.6538070000
C9	17.7742080000	-2.1624980000	54.3440600000
H10	17.9600320000	-1.1692790000	53.9311500000
H11	16.6913590000	-2.2589490000	54.4949700000
C12	18.2201500000	-3.2579510000	53.3548890000
C13	19.6977410000	-3.0566310000	52.9422510000
H14	20.3261410000	-3.2242630000	53.8223130000
H15	19.8196830000	-2.0140260000	52.6278550000
C16	20.1345830000	-3.9854680000	51.7908690000
H17	19.6224520000	-3.7240930000	50.8635230000
H18	19.8752160000	-5.0178190000	52.0502750000
C19	17.4002110000	-3.3422070000	52.0365660000
O20	17.0494680000	-4.5527860000	51.7000780000
O21	17.2509550000	-2.3310190000	51.3538550000
O22	18.0757880000	-4.5263620000	54.0242880000
C23	12.5687250000	-6.6977540000	54.3335480000
S24	12.8770900000	-4.0759290000	56.7814490000
S25	16.0568450000	-6.7721900000	55.6411480000
S26	9.5452340000	-4.9183980000	53.8141310000
S27	13.6422400000	-9.8945110000	55.1993630000
S28	14.6021730000	-4.0340980000	53.5158810000
S29	9.8539300000	-8.7871580000	53.8331710000
S30	15.0574120000	-7.4363900000	51.9961150000
S31	11.7779390000	-6.2588030000	50.7445370000
H32	13.6351740000	-2.1902110000	49.0425560000
C33	13.2979240000	-2.0241170000	50.0550230000
H34	13.8396120000	-2.7275350000	50.6922970000
H35	13.5371700000	-1.0022320000	50.3699400000
N36	11.8592600000	-2.2858520000	50.1040020000
H37	11.2951890000	-1.9251840000	49.3483260000
C38	11.1858980000	-2.7644290000	51.1649860000
N39	11.7647880000	-3.0214050000	52.3200640000
H40	11.2177370000	-3.5634840000	53.0057970000
H41	12.7776470000	-2.9942420000	52.4820760000
N42	9.8361830000	-2.9341220000	51.0614460000
H43	9.4777950000	-3.1662350000	50.1442140000
H44	9.4166110000	-3.4793030000	51.8263550000
H45	7.9298280000	0.3872090000	59.4123270000
C46	8.3594280000	-1.3525410000	60.6938030000
H47	8.1958710000	-1.5742720000	61.7553060000
H48	7.6128040000	-1.9247800000	60.1304230000
C49	9.7466830000	-1.8072330000	60.3560940000
N50	10.7266590000	-1.8605830000	61.3306840000
C51	10.2794930000	-2.1543240000	59.1341570000
H52	9.8487190000	-2.2642300000	58.1507720000
C53	11.8259530000	-2.2350230000	60.7018750000

H54	12.7908270000	-2.4074240000	61.1576250000
N55	11.6092530000	-2.4199900000	59.3715650000
H56	19.2870450000	-4.2769790000	57.5579940000
H57	16.7212050000	-8.8586260000	50.7826890000
C58	18.3717410000	-7.4260970000	50.6534100000
H59	17.7409240000	-6.5334500000	50.6273770000
H60	19.2060540000	-7.2456490000	49.9610780000
C61	18.9289500000	-7.5791550000	52.0357840000
N62	18.2242480000	-7.1911580000	53.1665200000
C63	20.1429940000	-8.0842840000	52.4361860000
H64	20.9735830000	-8.4826390000	51.8734930000
C65	18.9894260000	-7.4490560000	54.2158820000
H66	18.7532840000	-7.2272670000	55.2452630000
N67	20.1571780000	-8.0008330000	53.8181090000
H68	20.9219310000	-8.2146870000	54.4406970000
H69	6.9699720000	-10.5430430000	56.9222300000
C70	6.1750550000	-9.8249630000	56.6900010000
H71	5.5309550000	-10.2322280000	56.0424860000
H72	5.6932320000	-9.5371410000	57.6127200000
S73	7.9691770000	-7.7357500000	57.0397230000
H74	12.2758110000	-2.7063250000	58.6579010000
H75	18.2764600000	-4.3264190000	55.0183310000
C76	18.4389980000	-2.2628270000	55.7418970000
O77	18.6782070000	-3.4586090000	56.1763200000
O78	18.6579120000	-1.2112770000	56.3680150000
C79	21.6107590000	-3.9348800000	51.5280200000
O80	22.1698560000	-3.6557380000	50.4855880000
O81	22.3208170000	-4.2658020000	52.6398600000
H82	15.7024660000	-0.1996320000	61.4912310000
C83	14.8549940000	-0.0829910000	60.8320020000
H84	13.9165020000	-0.2867340000	61.3260940000
H85	14.8237740000	0.9913300000	60.5828130000
C86	15.0599850000	-0.8751950000	59.5441990000
H87	14.3456390000	-0.5403210000	58.7818240000
H88	14.8568460000	-1.9386520000	59.7200360000
C89	16.4847610000	-0.7515310000	58.9904020000
H90	16.8143060000	0.2982100000	59.0025380000
H91	16.5285990000	-1.0552570000	57.9382530000
C92	17.5134660000	-1.5853820000	59.7674730000
O93	17.2580260000	-2.1201060000	60.8524840000
N94	18.7349540000	-1.6670380000	59.1771390000
H95	19.3411040000	-2.3854840000	59.5548220000
H96	18.8210890000	-1.4822020000	58.1657990000
C97	10.6519930000	0.0100030000	53.3460010000
H98	11.1636990000	0.9592720000	53.2872700000
H99	9.5863320000	0.1850140000	53.3579370000
H100	10.9205140000	-0.5811730000	52.4705580000
C101	11.0819960000	-0.6480960000	54.6736640000
H102	10.7445870000	-1.6927140000	54.6820290000
C103	10.4059580000	0.0673020000	55.8535750000

H104	9.3122840000	-0.0048130000	55.7924690000
H105	10.6711650000	1.1344160000	55.8670030000
H106	10.7168930000	-0.3620370000	56.8126310000
C107	12.6081150000	-0.6226820000	54.8374620000
H108	13.1237070000	-1.1053340000	54.0008480000
H109	12.9189370000	-1.1457110000	55.7477350000
H110	12.9700740000	0.4139020000	54.8997200000
H111	13.4550780000	-12.2303880000	53.1883140000
C112	13.9110010000	-11.6210560000	52.4219860000
H113	14.7999470000	-11.1240650000	52.8144080000
H114	14.1319140000	-12.1992980000	51.5204300000
N115	12.8185210000	-10.6783750000	52.1483310000
H116	12.2655990000	-10.4313970000	52.9716350000
C117	12.5857430000	-9.9813010000	51.0474340000
N118	13.5632780000	-9.8499550000	50.1048020000
H119	13.2997770000	-9.2547330000	49.3268740000
H120	14.4502510000	-9.5408600000	50.5100750000
N121	11.3556370000	-9.4776640000	50.8222810000
H122	10.7024720000	-9.5387980000	51.6136760000
H123	11.3261450000	-8.5431910000	50.4012140000
H124	19.2400600000	-8.2269930000	61.6946540000
C125	19.5978940000	-7.2298790000	61.9900510000
H126	20.6372930000	-7.3367400000	62.3169200000
C127	18.8040000000	-6.7368900000	63.1929960000
O128	19.3308860000	-6.4063920000	64.2522040000
C129	19.4918710000	-6.2717990000	60.7990630000
H130	19.7871540000	-5.2630160000	61.1099850000
H131	18.4451010000	-6.1839090000	60.4886070000
C132	20.3150070000	-6.6936100000	59.5700040000
H133	20.1737520000	-7.7583340000	59.3518350000
H134	21.3889990000	-6.5639960000	59.7702540000
C135	20.0069270000	-5.9603570000	58.2561690000
O136	20.2075250000	-6.4871570000	57.1735300000
O137	19.5398340000	-4.7256620000	58.4293330000
N138	17.4569790000	-6.6923760000	62.9727560000
H139	17.1211320000	-6.8880850000	62.0386540000
C140	16.5368850000	-6.0058850000	63.8530400000
H141	15.7593560000	-6.7050570000	64.1923020000
H142	17.1158400000	-5.6992730000	64.7279710000
C143	15.8626230000	-4.7848820000	63.1835990000
H144	16.6176310000	-4.0296820000	62.9403100000
H145	15.1720330000	-4.3412140000	63.9133660000
C146	15.1254690000	-5.1967180000	61.9289330000
C147	13.8968510000	-5.8674800000	62.0062030000
H148	13.4110970000	-5.9910750000	62.9726380000
C149	15.7179770000	-5.0125120000	60.6690140000
H150	16.6326140000	-4.4313770000	60.5956900000
C151	13.2918230000	-6.3777120000	60.8562330000
H152	12.3293560000	-6.8798460000	60.9263080000
C153	15.1245400000	-5.5356820000	59.5178320000

H154	15.5891310000	-5.3958740000	58.5451430000
C155	13.9194970000	-6.2348790000	59.6141670000
H156	13.4777390000	-6.6585760000	58.7187450000
H157	9.0037560000	0.7050730000	60.7752690000
C158	8.1269810000	0.1560800000	60.4649990000
H159	7.2836720000	0.4592420000	61.0677700000
H160	11.0432070000	-7.4098360000	58.1304620000
H161	18.2592790000	-9.4987520000	50.1505400000
C162	17.5839750000	-8.6559210000	50.1520280000
H163	17.2773630000	-8.4419890000	49.1387990000
H164	23.2576950000	-4.1981950000	52.3776050000
H165	9.9777890000	-9.2325050000	56.6824490000
H166	12.6115460000	-4.8410160000	57.8618490000
C167	6.7343310000	-8.5961550000	55.9971410000
H168	7.2060380000	-8.8843660000	55.0517090000
H169	5.9161250000	-7.9028730000	55.7630500000
N170	11.0040590000	-9.2064260000	56.7276240000
N171	11.0114090000	-8.3610880000	58.4937070000
H172	7.1493940000	-5.8354100000	59.8218940000
C173	6.9590150000	-4.8009340000	59.5000030000
H174	5.9039260000	-4.4903550000	59.5181450000
H175	7.4844910000	-4.1757740000	60.2379000000
C176	7.4721080000	-4.4410820000	58.1021270000
H177	8.4930870000	-4.8133310000	57.9421900000
H178	7.5097660000	-3.3475940000	58.0173680000
O179	6.6043910000	-4.8843580000	57.0696150000
H180	6.8562240000	-5.8206520000	56.9200210000
H181	14.9302850000	-9.6716190000	57.0338500000
H182	11.3671230000	-10.1168180000	57.0178090000
H183	10.4526910000	-6.2610250000	56.4471970000
H184	10.7897210000	-5.3411510000	50.6867360000
H185	10.1375540000	-8.5400480000	58.9814830000
O186	13.3699460000	-9.4170390000	59.6500290000
H187	11.8773260000	-8.7001730000	58.9564140000
H188	13.6736890000	-8.7814130000	60.3172500000
O189	15.4101990000	-9.3651230000	57.8412560000
H190	14.1039740000	-9.3992340000	58.9730410000
H191	15.7268960000	-8.4897460000	57.5461920000

The NH_2 product structure after N-N cleavage in E_8
Energies: E= -7975.705195 solv = -0.201346, disp = --237.41 Z_0 = 897.83

Mo1	16.5103748855	-5.6305326137	53.2683216176
Fe2	10.0120670509	-7.7338201542	56.5414068291
Fe3	11.3223279916	-5.3038376167	55.5196557458
Fe4	10.9705715146	-7.0171681839	53.5920575599
Fe5	12.4684672109	-8.4418913308	55.3490985709
Fe6	14.6855696340	-7.6048289933	54.2873177532
Fe7	14.1610450250	-5.2048019395	55.1389719745

Fe8	13.2743325368	-5.8987303956	52.5896451833
C9	17.8027693474	-2.0334113083	54.2309676316
H10	17.9686515432	-1.0217398932	53.8560852991
H11	16.7200255461	-2.1626090231	54.3573265152
C12	18.3061866030	-3.0884374198	53.2207417937
C13	19.7777992964	-2.8189323894	52.8314158690
H14	20.3980444902	-2.9903541487	53.7165843667
H15	19.8684675919	-1.7649650414	52.5476705168
C16	20.2614682970	-3.7012847129	51.6592649799
H17	19.8359372550	-3.3551688862	50.7158948486
H18	19.9344217622	-4.7320238903	51.8359318372
C19	17.5028866101	-3.2062823995	51.8918337336
O20	17.1358882145	-4.4269331877	51.6015979080
O21	17.3700812031	-2.2218225200	51.1693773204
O22	18.2088474639	-4.3685552259	53.8708982499
C23	12.8259675927	-6.7068495659	54.4572149016
S24	12.9665995987	-4.0997212445	56.9105544790
S25	16.2070122078	-6.3441820520	55.5770066690
S26	9.7853251632	-5.0321851170	53.7385497639
S27	14.3211878288	-9.6834616042	55.1599793463
S28	14.6057090530	-3.9375642661	53.2898306090
S29	10.3754849271	-9.1151919060	54.3441650182
S30	15.1802864140	-7.2694394942	52.0380959867
S31	11.9935004332	-6.0968410510	50.5318518221
H32	13.6351742804#	-2.1902108415#	49.0425560624#
C33	13.2979239566#	-2.0241171916#	50.0550237169#
H34	13.8195346655	-2.7443934920	50.6887943096
H35	13.5534692524	-1.0079186064	50.3749924497
N36	11.8560430044	-2.2586090580	50.0851954440
H37	11.3135632395	-2.0006096770	49.2743026789
C38	11.1781699664	-2.7777873752	51.1189383905
N39	11.7268654248	-2.9693117107	52.3004071607
H40	11.2173319209	-3.6032197219	52.9512863548
H41	12.7292643881	-2.8701569795	52.4635494406
N42	9.8443532389	-3.0380918546	50.9666118226
H43	9.5793514846	-3.3939749819	50.0558029169
H44	9.4559436302	-3.5939091974	51.7395305159
H45	7.9334543069	0.3851366470	59.4116894467
C46	8.3377741314	-1.3544962370	60.7008543127
H47	8.1566592022	-1.5724991813	61.7603551121
H48	7.5916695576	-1.9189362081	60.1302125073
C49	9.7185025985	-1.8330354989	60.3813412368
N50	10.6981746695	-1.8583875715	61.3576944082
C51	10.2417999043	-2.2432659408	59.1769509809
H52	9.8041586996	-2.3901495389	58.2018987624
C53	11.7873391765	-2.2833189653	60.7453901560
H54	12.7521879454	-2.4462798121	61.2051021213
N55	11.5665173966	-2.5209744838	59.4254551602
H56	19.2380966120	-4.3243012440	57.5038303117
H57	16.7267248062	-8.8558129634	50.7901013450

C58	18.3728833274	-7.4130774705	50.6211127764
H59	17.7425416812	-6.5216144459	50.5705214784
H60	19.2000085806	-7.2515996983	49.9155775945
C61	18.9441011333	-7.5206372718	52.0009472161
N62	18.2877123202	-7.0360452918	53.1232191748
C63	20.1344685696	-8.0754568280	52.4037781104
H64	20.9266911899	-8.5515796129	51.8469882410
C65	19.0580719902	-7.2899056637	54.1704709521
H66	18.8630352520	-7.0112687500	55.1943401554
N67	20.1813301652	-7.9278506823	53.7790171577
H68	20.9391960579	-8.1686887222	54.4003940614
H69	6.9092333436	-10.5960850603	56.9577675343
C70	6.1750558632#	-9.8249627632#	56.6900012582#
H71	5.5309553798#	-10.2322276921#	56.0424858143#
H72	5.6932323286#	-9.5371413468#	57.6127204349#
S73	7.9778887407	-8.0024585517	57.5565414023
H74	12.2278045807	-2.8654657318	58.7344696900
H75	18.4109323925	-4.1640414254	54.8771417368
C76	18.4360019291	-2.1568423575	55.6473380303
O77	18.7306275113	-3.3551338499	56.0440085381
O78	18.5800571423	-1.1206868816	56.3180197834
C79	21.7561233601	-3.7069096303	51.5354138714
O80	22.4354566658	-3.2933690280	50.6165494161
O81	22.3208167060#	-4.2658019333#	52.6398595966#
H82	15.7024655335#	-0.1996319797#	61.4912314270#
C83	14.8549940177#	-0.0829906878#	60.8320019102#
H84	13.9165015893#	-0.2867338795#	61.3260937710#
H85	14.8237513049	0.9909198040	60.5820462280
C86	15.0599852104#	-0.8751946515#	59.5441989690#
H87	14.3407573950	-0.5429444176	58.7851873019
H88	14.8601781205	-1.9389074838	59.7202199411
C89	16.4822679815	-0.7447827264	58.9869813652
H90	16.8199688489	0.3015327344	59.0324754898
H91	16.5205454494	-1.0130396161	57.9252861869
C92	17.5061991611	-1.6130377968	59.7308573595
O93	17.2583628707	-2.1659277406	60.8088933627
N94	18.7149325845	-1.7086079214	59.1162633720
H95	19.3142944190	-2.4464061616	59.4663081423
H96	18.7898255209	-1.4876204695	58.1121839689
C97	10.6519928910#	0.0100025324#	53.3460010909#
H98	11.1636988178#	0.9592721578#	53.2872699589#
H99	9.5863320881#	0.1850135700#	53.3579374863#
H100	10.9184398470	-0.5796790807	52.4690596341
C101	11.0681706045	-0.6646535656	54.6682039380
H102	10.7374337249	-1.7115409570	54.6470294880
C103	10.3700788402	0.0217128618	55.8523073926
H104	9.2783700917	-0.0544415234	55.7688021248
H105	10.6296875338	1.0893588379	55.8947116834
H106	10.6642479239	-0.4280624379	56.8069150818
C107	12.5909453114	-0.6382763618	54.8565672350

H108	13.1211926650	-1.1036930398	54.0192109530
H109	12.8894890359	-1.1745554670	55.7639178604
H110	12.9496698406	0.3973024697	54.9444426808
H111	13.4550780839#	-12.2303879923#	53.1883144463#
C112	13.9110013049#	-11.6210559987#	52.4219861260#
H113	14.8032777362	-11.1327233348	52.8190498362
H114	14.1204583867	-12.2019768926	51.5192163759
N115	12.8228847988	-10.6664352188	52.1654082476
H116	12.2017187135	-10.4813356580	52.9562962635
C117	12.6669717061	-9.8523957902	51.1308133873
N118	13.6749128051	-9.6796504198	50.2308353866
H119	13.4761973957	-8.9621737191	49.5403854697
H120	14.5770677089	-9.5048046536	50.6728224517
N121	11.4768821234	-9.2591270973	50.9177422313
H122	10.7968562113	-9.3824023615	51.6698122727
H123	11.5316946466	-8.2714657454	50.6026907890
H124	19.2000469019	-8.2164361960	61.7116899160
C125	19.5978939089#	-7.2298787609#	61.9900508685#
H126	20.6401818445	-7.3669751318	62.2963922264
C127	18.8359479569	-6.6829135129	63.1958051411
O128	19.3882010541	-6.2193320413	64.1904549654
C129	19.4918709150#	-6.2717990526#	60.7990629492#
H130	19.8795942917	-5.2878343717	61.0879333182
H131	18.4374654520	-6.1095093604	60.5543422295
C132	20.2096230937	-6.7573701409	59.5265418223
H133	19.9434125953	-7.7989402831	59.3082117225
H134	21.2993116771	-6.7584924174	59.6764321436
C135	19.9540608117	-5.9954249692	58.2155610122
O136	20.2480434879	-6.4834370518	57.1351708894
O137	19.4239816685	-4.7828491943	58.3779477357
N138	17.4822275931	-6.7411103284	63.0359283529
H139	17.1300180021	-7.0516307304	62.1394525475
C140	16.5368850864#	-6.0058847553#	63.8530404357#
H141	15.8391059970	-6.7121364172	64.3254370849
H142	17.1093373288	-5.5232474538	64.6489909235
C143	15.7337311865	-4.9654050601	63.0298464501
H144	16.3699975617	-4.1034765995	62.8028529221
H145	14.9058255902	-4.6066235394	63.6562601908
C146	15.2190132207	-5.5542943624	61.7294106882
C147	14.2118000775	-6.5327034062	61.7094097674
H148	13.7074727735	-6.8078842036	62.6341761735
C149	15.8362067904	-5.1992358057	60.5180975311
H150	16.5849970436	-4.4113833667	60.5171099821
C151	13.8567137179	-7.1665254510	60.5151866396
H152	13.0800704831	-7.9276218262	60.5183071981
C153	15.4877394007	-5.8356800513	59.3235984431
H154	15.9809653586	-5.5644709536	58.3948107788
C155	14.5083976016	-6.8332841528	59.3222627506
H156	14.2777886939	-7.3547516910	58.3975086645
H157	9.0037560265#	0.7050733753#	60.7752693193#

C158	8.1269804207#	0.1560798469#	60.4649991883#
H159	7.2836721050#	0.4592420325#	61.0677701356#
H161	18.2592787788#	-9.4987523744#	50.1505399120#
C162	17.5839746111#	-8.6559206771#	50.1520277116#
H163	17.2773626429#	-8.4419890564#	49.1387987944#
H164	23.2842008203	-4.2118015441	52.4975452039
H166	12.2392239751	-7.6872773526	57.8664173451
H167	13.0130806838	-5.0908969577	57.8284535968
C168	6.9145180352	-8.6051099154	56.1678537679
H169	7.5322726390	-8.8411034348	55.2972027588
H170	6.2138710660	-7.8112354279	55.8829248122
N171	11.7998045563	-8.3700398096	57.2534248636
H173	7.1493939764#	-5.8354098452#	59.8218945107#
C174	6.9590152844#	-4.8009335482#	59.5000032947#
H175	5.9039259738#	-4.4903550797#	59.5181448269#
H176	7.4839536513	-4.1666116773	60.2295079389
C177	7.4968051070	-4.4832438303	58.1021855523
H178	8.5548520580	-4.7653298171	58.0183486631
H179	7.4340834859	-3.3988177763	57.9366544427
O180	6.7343239452	-5.0884345118	57.0701634288
H181	7.0935561961	-5.9972537803	57.0099056810
H182	10.7692712088	-5.7379798188	50.9821609512
H183	11.8670915818	-9.2868531756	57.6936052922
H184	10.3685231962	-5.9902470553	56.7809696835

The E_8 TS structure in **Figure 10**
Energies: E= -7975.683911 solv = -0.201775, disp = --234.24 Z_0 = 896.38

Mo1	16.4912970000	-5.8067090000	53.2842630000
Fe2	9.6629450000	-6.9694180000	56.5293770000
Fe3	11.7172010000	-5.3954800000	55.9414260000
Fe4	10.8497970000	-6.4840100000	53.6213590000
Fe5	12.0243270000	-8.5014330000	54.9660580000
Fe6	14.5137560000	-7.7120990000	54.4681670000
Fe7	14.2692560000	-5.2397890000	55.0589050000
Fe8	13.2610830000	-6.1454660000	52.5659390000
C9	17.7593180000	-2.1611280000	54.2849400000
H10	17.9180730000	-1.1548050000	53.8927880000
H11	16.6789350000	-2.2929060000	54.4273270000
C12	18.2532630000	-3.2292230000	53.2866450000
C13	19.7243580000	-2.9673490000	52.8869020000
H14	20.3517890000	-3.1325140000	53.7679650000
H15	19.8150850000	-1.9157110000	52.5932090000
C16	20.1960850000	-3.8596130000	51.7189090000
H17	19.7235220000	-3.5565340000	50.7833330000
H18	19.9159290000	-4.8968940000	51.9323830000
C19	17.4616640000	-3.3619560000	51.9555770000
O20	17.1885130000	-4.5998380000	51.6318270000

O21	17.2655740000	-2.3731130000	51.2554950000
O22	18.1493610000	-4.5024870000	53.9464920000
C23	12.7251880000	-6.6365460000	54.5417180000
S24	13.4441630000	-3.9783830000	56.9320000000
S25	16.1823860000	-6.5303650000	55.6069940000
S26	9.6451960000	-4.9806470000	54.8527810000
S27	13.8821040000	-9.7514540000	55.2387320000
S28	14.6298440000	-4.1276020000	53.1131670000
S29	10.4288150000	-8.9489470000	53.1874680000
S30	15.1373530000	-7.5504870000	52.1975140000
S31	11.3698050000	-6.4629130000	51.1420460000
H32	13.6351740000	-2.1902110000	49.0425560000
C33	13.2979240000	-2.0241170000	50.0550240000
H34	13.9701430000	-2.5655980000	50.7262390000
H35	13.3211800000	-0.9545150000	50.2916440000
N36	11.9280680000	-2.5610940000	50.1370600000
H37	11.3530800000	-2.4634270000	49.3124430000
C38	11.2692290000	-3.0215670000	51.2121820000
N39	11.7773780000	-3.0050450000	52.4311820000
H40	11.3198370000	-3.5729580000	53.1482350000
H41	12.7791240000	-2.8737430000	52.5901150000
N42	9.9734220000	-3.4400670000	51.0270000000
H43	9.9051740000	-4.1332440000	50.2838130000
H44	9.5811800000	-3.8557530000	51.8721350000
H45	7.9309890000	0.3873630000	59.4122450000
C46	8.3434900000	-1.3544580000	60.6958980000
H47	8.1753410000	-1.5699640000	61.7585170000
H48	7.5866280000	-1.9166660000	60.1373270000
C49	9.7156100000	-1.8501330000	60.3565670000
N50	10.7287660000	-1.8264900000	61.2982130000
C51	10.1868050000	-2.3700630000	59.1717410000
H52	9.7127050000	-2.5840600000	58.2267200000
C53	11.7843110000	-2.3317380000	60.6865860000
H54	12.7609540000	-2.4819980000	61.1255110000
N55	11.5094690000	-2.6702320000	59.3981640000
H56	19.2540360000	-4.3377630000	57.5097770000
H57	16.7321130000	-8.8387090000	50.8011900000
C58	18.4004590000	-7.4328960000	50.6274210000
H59	17.7912750000	-6.5263500000	50.5946530000
H60	19.2278280000	-7.2815220000	49.9202200000
C61	18.9772420000	-7.5763310000	52.0027480000
N62	18.3012310000	-7.1568950000	53.1392000000
C63	20.1881400000	-8.0971110000	52.3911390000
H64	21.0017100000	-8.5190850000	51.8211030000
C65	19.0792880000	-7.4103460000	54.1802840000
H66	18.8726210000	-7.1624780000	55.2101420000
N67	20.2284380000	-7.9900640000	53.7703560000
H68	20.9972640000	-8.2161680000	54.3835700000
H69	6.9217980000	-10.5830980000	56.9617180000
C70	6.1750560000	-9.8249630000	56.6900010000

H71	5.5309550000	-10.2322280000	56.0424860000
H72	5.6932320000	-9.5371410000	57.6127200000
S73	7.7187930000	-7.7291610000	57.5168620000
H74	12.1355510000	-3.0894620000	58.7149320000
H75	18.3486610000	-4.2958460000	54.9501540000
C76	18.4167360000	-2.2654050000	55.6918570000
O77	18.6995340000	-3.4630260000	56.1006120000
O78	18.5916220000	-1.2243300000	56.3435880000
C79	21.6817950000	-3.8109180000	51.5297260000
O80	22.2992720000	-3.4365440000	50.5526860000
O81	22.3208170000	-4.2658020000	52.6398600000
H82	15.7024660000	-0.1996320000	61.4912310000
C83	14.8549940000	-0.0829910000	60.8320020000
H84	13.9165020000	-0.2867340000	61.3260940000
H85	14.8281050000	0.9922200000	60.5870110000
C86	15.0599850000	-0.8751950000	59.5441990000
H87	14.3657270000	-0.5148200000	58.7749990000
H88	14.8130510000	-1.9294510000	59.7130110000
C89	16.4906560000	-0.8137850000	59.0050050000
H90	16.8966150000	0.2062570000	59.0751790000
H91	16.5198640000	-1.0632220000	57.9378330000
C92	17.4491390000	-1.7669550000	59.7302550000
O93	17.1276610000	-2.3982770000	60.7412350000
N94	18.6917050000	-1.8390330000	59.1754200000
H95	19.2415690000	-2.6335410000	59.4810900000
H96	18.7965050000	-1.5808340000	58.1848040000
C97	10.6519930000	0.0100020000	53.3460010000
H98	11.1636990000	0.9592720000	53.2872700000
H99	9.5863320000	0.1850140000	53.3579380000
H100	10.9205570000	-0.5845600000	52.4704850000
C101	11.0731590000	-0.6553370000	54.6736040000
H102	10.7470950000	-1.7037360000	54.6655840000
C103	10.3744550000	0.0310210000	55.8580930000
H104	9.2828460000	-0.0404850000	55.7714600000
H105	10.6387850000	1.0972150000	55.9060360000
H106	10.6636420000	-0.4265450000	56.8108070000
C107	12.5974020000	-0.6160230000	54.8590940000
H108	13.1313600000	-1.0622260000	54.0127110000
H109	12.9067730000	-1.1571910000	55.7597470000
H110	12.9456610000	0.4222920000	54.9549620000
H111	13.4550780000	-12.2303880000	53.1883140000
C112	13.9110010000	-11.6210560000	52.4219860000
H113	14.6705770000	-10.9931930000	52.8962640000
H114	14.3490030000	-12.2566080000	51.6447350000
N115	12.8370600000	-10.8187700000	51.8413780000
H116	12.0743430000	-10.5095120000	52.4652000000
C117	12.9398570000	-10.1283610000	50.7170880000
N118	14.1281710000	-10.0568160000	50.0604780000
H119	14.1211500000	-9.4483000000	49.2504480000
H120	14.9216000000	-9.8634510000	50.6679710000

N121	11.8375940000	-9.5767620000	50.1548680000
H122	11.0142120000	-9.6481600000	50.7567070000
H123	11.9477030000	-8.5960390000	49.8781520000
H124	19.2064160000	-8.2179550000	61.7082320000
C125	19.5978940000	-7.2298790000	61.9900510000
H126	20.6406320000	-7.3607150000	62.2979780000
C127	18.8339690000	-6.6855830000	63.1944290000
O128	19.3898410000	-6.2079190000	64.1798740000
C129	19.4918710000	-6.2717990000	60.7990630000
H130	19.8355120000	-5.2751770000	61.0986940000
H131	18.4391660000	-6.1486840000	60.5252480000
C132	20.2641700000	-6.7364320000	59.5520250000
H133	20.0733910000	-7.7971370000	59.3506700000
H134	21.3479450000	-6.6546540000	59.7229650000
C135	19.9665600000	-6.0126130000	58.2316450000
O136	20.1882650000	-6.5429180000	57.1540900000
O137	19.4851730000	-4.7811460000	58.3862860000
N138	17.4798880000	-6.7541370000	63.0436160000
H139	17.1230360000	-7.0841820000	62.1555610000
C140	16.5368850000	-6.0058850000	63.8530400000
H141	15.8789520000	-6.7022150000	64.3928240000
H142	17.1195590000	-5.4537130000	64.5947340000
C143	15.6749680000	-5.0465960000	62.9923760000
H144	16.2950300000	-4.2201950000	62.6279110000
H145	14.9014410000	-4.6168950000	63.6427340000
C146	15.0459960000	-5.7636860000	61.8124990000
C147	13.9665380000	-6.6449560000	61.9826450000
H148	13.5076150000	-6.7467830000	62.9648280000
C149	15.6053760000	-5.6326260000	60.5313240000
H150	16.4044530000	-4.9114940000	60.3751210000
C151	13.4723890000	-7.3897360000	60.9099380000
H152	12.6277470000	-8.0580140000	61.0591280000
C153	15.1290400000	-6.3965030000	59.4595940000
H154	15.5917890000	-6.3049090000	58.4799400000
C155	14.0630050000	-7.2807040000	59.6472520000
H156	13.6959890000	-7.8695430000	58.8107640000
H157	9.0037560000	0.7050730000	60.7752690000
C158	8.1269800000	0.1560800000	60.4649990000
H159	7.2836720000	0.4592420000	61.0677700000
H160	18.2592790000	-9.4987520000	50.1505400000
C161	17.5839740000	-8.6559210000	50.1520280000
H162	17.2773630000	-8.4419890000	49.1387990000
H163	23.2731150000	-4.1847890000	52.4454200000
H164	11.3961070000	-8.5983310000	57.4563050000
H165	13.8207000000	-4.8588210000	57.8873810000
C166	6.8766900000	-8.5974580000	56.1209440000
H167	7.5908190000	-8.8746570000	55.3377880000
H168	6.1477410000	-7.9131940000	55.6696670000
N169	10.8614000000	-8.6189660000	56.5869580000
H170	7.1493940000	-5.8354100000	59.8218950000

C171	6.9590150000	-4.8009340000	59.5000030000
H172	5.9039260000	-4.4903550000	59.5181450000
H173	7.4854020000	-4.1749100000	60.2359960000
C174	7.4411520000	-4.4107980000	58.0993180000
H175	8.4615640000	-4.7704930000	57.9093950000
H176	7.4688300000	-3.3150500000	58.0374120000
O177	6.5503840000	-4.8277590000	57.0769750000
H178	6.7392360000	-5.7846340000	56.9764180000
H179	10.9154110000	-7.8241850000	51.9800580000
H180	10.3099720000	-9.4777050000	56.5978720000
H181	10.9047910000	-5.9601730000	57.4180840000

The E_8 product structure after the TS in **Figure 10**
Energies: E= -7975.700570 solv = -0.193900, disp = --236.30 Z_0 = 898.38

Mo1	16.4619898546	-5.8133304129	53.2352309374
Fe2	9.5852959549	-6.9218156869	56.4684036216
Fe3	11.5656460241	-5.3774982386	55.6827130627
Fe4	10.8661064868	-6.3807104484	53.2532827852
Fe5	11.9247299952	-8.4231436456	54.9908055423
Fe6	14.3919665281	-7.7170011093	54.3201883693
Fe7	14.1474622708	-5.2401289484	54.9363275886
Fe8	13.2411544181	-6.1002986330	52.3348094353
C9	17.7260073534	-2.1946934586	54.1985529254
H10	17.8838726524	-1.1872226939	53.8090969216
H11	16.6443076930	-2.3345029091	54.3212562442
C12	18.2482885549	-3.2610128013	53.2105898847
C13	19.7258005259	-2.9879766210	52.8428849728
H14	20.3370930018	-3.1672546680	53.7322821261
H15	19.8168598831	-1.9300802128	52.5731733870
C16	20.2271672335	-3.8504357152	51.6642759115
H17	19.7752824763	-3.5247613946	50.7261972788
H18	19.9466161380	-4.8943193735	51.8444866378
C19	17.4850182013	-3.4059945326	51.8619883855
O20	17.1788251878	-4.6425281315	51.5659728310
O21	17.3390717309	-2.4298617906	51.1309639583
O22	18.1364247697	-4.5339492299	53.8756053027
C23	12.6070486427	-6.6456825460	54.3410372471
S24	13.2917408939	-3.9721118575	56.7600516206
S25	16.0379208072	-6.5428545128	55.5334970958
S26	9.4652170644	-5.1550407461	54.5934497522
S27	13.7770423890	-9.6892808480	55.2316063010
S28	14.6229568836	-4.1241432425	53.0204775637
S29	10.2205223154	-9.7205587422	53.7476884188
S30	15.1179029555	-7.5272992911	52.0996168046
S31	11.3523372402	-6.3230087458	50.9723703789
H32	13.6351741995#	-2.1902105078#	49.0425559809#
C33	13.2979242668#	-2.0241175639#	50.0550236550#

H34	13.9557600878	-2.5888872049	50.7210596314
H35	13.3530042866	-0.9567980680	50.2971625826
N36	11.9150178549	-2.5169431161	50.1448326288
H37	11.3422834849	-2.4511830758	49.3152555099
C38	11.2610031208	-2.9621605723	51.2243675007
N39	11.7773784559	-2.9259512172	52.4417957646
H40	11.3465003396	-3.5207318589	53.1509143669
H41	12.7781490154	-2.7820418744	52.5851743651
N42	9.9631824522	-3.3838803991	51.0408946949
H43	9.9495307233	-4.1868230104	50.4048539243
H44	9.5413038039	-3.6968319883	51.9163899060
H45	7.9316363442	0.3821175125	59.4113119501
C46	8.3384542664	-1.3515820778	60.7047232138
H47	8.2408128769	-1.5502231076	61.7790920271
H48	7.5378003552	-1.9113514041	60.2070004949
C49	9.6785535879	-1.8666193853	60.2820813229
N50	10.7270383278	-1.9322092288	61.1819150449
C51	10.0896067762	-2.3139821971	59.0474346808
H52	9.5742154289	-2.4493192865	58.1100667050
C53	11.7439514495	-2.4195484100	60.4956630301
H54	12.7325349246	-2.6221080661	60.8840730059
N55	11.4112444685	-2.6609156205	59.1995751809
H56	19.1928614546	-4.3870846756	57.4679986299
H57	16.7311088151	-8.8489946665	50.7947358606
C58	18.4051478096	-7.4378242886	50.6269240502
H59	17.8077610493	-6.5246272027	50.5766960189
H60	19.2452142657	-7.3017212452	49.9316301249
C61	18.9579809718	-7.5797354149	52.0113840729
N62	18.2425529456	-7.2005505622	53.1381307964
C63	20.1709334490	-8.0771726320	52.4209950561
H64	21.0071515137	-8.4711642360	51.8644543227
C65	18.9995314890	-7.4574306850	54.1937451230
H66	18.7610750274	-7.2349945897	55.2222164070
N67	20.1724380051	-8.0000208912	53.8036367517
H68	20.9342063763	-8.2084027138	54.4321254490
H69	6.9177916295	-10.5869472667	56.9601120240
C70	6.1750551700#	-9.8249629507#	56.6900008657#
H71	5.5309553835#	-10.2322281189#	56.0424860788#
H72	5.6932324990#	-9.5371412167#	57.6127204830#
S73	7.7440865184	-7.7808248289	57.5459688042
H74	11.9991234944	-3.0574341970	58.4700589462
H75	18.3293044101	-4.3181145143	54.8803129510
C76	18.3562031000	-2.2911572317	55.6192479720
O77	18.6556686267	-3.4847346558	56.0302816376
O78	18.4960238514	-1.2470388373	56.2763726182
C79	21.7175589751	-3.7956284504	51.5157701745
O80	22.3645509360	-3.4078036337	50.5630485085
O81	22.3208168069#	-4.2658019565#	52.6398596131#
H82	15.7024655131#	-0.1996320169#	61.4912314468#
C83	14.8549938727#	-0.0829906515#	60.8320018651#

H84	13.9165015892#	-0.2867338796#	61.3260937710#
H85	14.8269425083	0.9914782138	60.5841288938
C86	15.0599853417#	-0.8751946134#	59.5441990775#
H87	14.3582424537	-0.5237249499	58.7776843915
H88	14.8282327937	-1.9329010468	59.7142595528
C89	16.4904201451	-0.7889680410	59.0038100345
H90	16.8833155395	0.2341029546	59.1038046656
H91	16.5251562510	-1.0074738519	57.9306010902
C92	17.4588871079	-1.7463983486	59.7101321942
O93	17.1730717485	-2.3389067498	60.7563028982
N94	18.6681259488	-1.8697710462	59.0976314150
H95	19.2198513548	-2.6622975130	59.4048894428
H96	18.7426599138	-1.6245426123	58.1006173137
C97	10.6519925619#	0.0100027909#	53.3460013450#
H98	11.1636989448#	0.9592720843#	53.2872698826#
H99	9.5863320827#	0.1850135445#	53.3579373790#
H100	10.9225999448	-0.5867343423	52.4747373866
C101	11.0671717980	-0.6410871650	54.6821242388
H102	10.7545550498	-1.6935220409	54.6796866535
C103	10.3438500796	0.0477621321	55.8492296518
H104	9.2551277359	-0.0495849343	55.7534517699
H105	10.5838108043	1.1202005148	55.8801459144
H106	10.6336731188	-0.3861510694	56.8123291577
C107	12.5872546870	-0.5808216416	54.8892031718
H108	13.1401031763	-1.0376189826	54.0607378981
H109	12.8875975048	-1.0997608316	55.8053649351
H110	12.9210005877	0.4635268912	54.9690023510
H111	13.4550780078#	-12.2303881957#	53.1883142395#
C112	13.9110014738#	-11.6210558262#	52.4219864472#
H113	14.6770935706	-10.9881787121	52.8768970870
H114	14.3227991006	-12.2418611272	51.6196778935
N115	12.7770892558	-10.8386510458	51.9254922085
H116	12.0047901422	-10.6844840486	52.5864285753
C117	12.7364269288	-10.0352472887	50.8730181411
N118	13.8475165697	-9.8080976231	50.1230859232
H119	13.7116880733	-9.1010170712	49.4083501627
H120	14.6780617668	-9.6029609099	50.6776155594
N121	11.5541122457	-9.5028029166	50.4895618871
H122	10.7990895741	-9.7188374560	51.1413562985
H123	11.5691859009	-8.4769195482	50.3261133086
H124	19.1925451970	-8.2137892490	61.7139070437
C125	19.5978937322#	-7.2298789825#	61.9900506276#
H126	20.6406135812	-7.3715406847	62.2926664380
C127	18.8405558849	-6.6689839027	63.1936725267
O128	19.3979492744	-6.1479708162	64.1562320143
C129	19.4918712562#	-6.2717988555#	60.7990631515#
H130	19.8976514985	-5.2928932219	61.0791111127
H131	18.4366194664	-6.0978558968	60.5669089502
C132	20.1890906469	-6.7836041058	59.5242071780
H133	19.9185842690	-7.8295134242	59.3336194006

H134	21.2809172536	-6.7833194893	59.6574423039
C135	19.9123367994	-6.0517880838	58.2014310369
O136	20.1705810432	-6.5705261186	57.1258421590
O137	19.4029780900	-4.8297511423	58.3479681425
N138	17.4847932117	-6.7761468730	63.0667563988
H139	17.1267377637	-7.1276355757	62.1872577577
C140	16.5368852325#	-6.0058846124#	63.8530406696#
H141	15.8592966119	-6.6909923551	64.3820730047
H142	17.1126237387	-5.4598404041	64.6042825842
C143	15.7089054097	-5.0375408677	62.9690838311
H144	16.3417835401	-4.2056362148	62.6420697682
H145	14.9091237999	-4.6143520645	63.5917276728
C146	15.1266933992	-5.7288207254	61.7482757541
C147	14.0740032887	-6.6528908186	61.8591820101
H148	13.6087348074	-6.8229253763	62.8287848759
C149	15.6929529629	-5.5103060718	60.4812783253
H150	16.4729290638	-4.7604787906	60.3732053853
C151	13.6121475894	-7.3519156494	60.7411637159
H152	12.7920673522	-8.0578547367	60.8461372209
C153	15.2429401687	-6.2201766889	59.3619033822
H154	15.7036216135	-6.0554943174	58.3915587754
C155	14.2047572893	-7.1481051496	59.4899169618
H156	13.8687900874	-7.7068015411	58.6201586728
H157	9.0037560289#	0.7050733872#	60.7752692906#
C158	8.1269803621#	0.1560798163#	60.4649993325#
H159	7.2836721017#	0.4592420650#	61.0677701150#
H160	10.5493233227	-9.2817440669	56.9617666445
H161	18.2592786163#	-9.4987525050#	50.1505400854#
C162	17.5839744213#	-8.6559206467#	50.1520273648#
H163	17.2773628825#	-8.4419889546#	49.1387987430#
H164	23.2790956805	-4.1825582305	52.4781010831
H165	9.5412829687	-8.7509849541	53.0900289995
H166	13.5657083301	-4.8816532265	57.7237918895
C168	6.8890996318	-8.6048560653	56.1320579249
H169	7.6036435202	-8.8911296165	55.3532223984
H170	6.1736140548	-7.9015802441	55.6885267649
H171	11.5636707162	-8.1118637068	57.5297592543
N172	10.9692492633	-8.3776145766	56.7440519142
H173	7.1493940090#	-5.8354099042#	59.8218943024#
C174	6.9590154294#	-4.8009333139#	59.5000033092#
H175	5.9039259954#	-4.4903550095#	59.5181448941#
H176	7.4948229270	-4.1684463567	60.2223309863
C177	7.4252055024	-4.4385439192	58.0865716743
H178	8.4519298373	-4.7826344624	57.9000466117
H179	7.4294230533	-3.3454099282	57.9922328846
O180	6.5354669751	-4.9050279111	57.0839578493
H181	6.7265558277	-5.8651766030	57.0271538703
H183	10.7614693288	-5.7429927680	57.2113947083

The E_8 TS structure in **Figure 11**

Energies: E= -7975.690095 solv = -0.198321, disp = --238.46 Z_0 = 897.60

Mo1	16.5894810000	-5.6906690000	53.2641290000
Fe2	10.0209620000	-6.6719790000	56.6663340000
Fe3	11.8590170000	-5.1444320000	55.7608730000
Fe4	10.9286890000	-6.8225890000	53.5472700000
Fe5	12.3130620000	-8.2720230000	55.4847370000
Fe6	14.6668640000	-7.6389080000	54.4272940000
Fe7	14.4467620000	-5.1995070000	55.0879930000
Fe8	13.3147240000	-5.9994140000	52.6369380000
C9	17.8279950000	-2.0749520000	54.2951720000
H10	17.9706460000	-1.0567470000	53.9277050000
H11	16.7496820000	-2.2263550000	54.4317860000
C12	18.3392180000	-3.1093320000	53.2700250000
C13	19.8015920000	-2.8091620000	52.8679520000
H14	20.4356560000	-2.9945270000	53.7401350000
H15	19.8772430000	-1.7480230000	52.6069030000
C16	20.2739560000	-3.6594210000	51.6673330000
H17	19.8519160000	-3.2797760000	50.7355610000
H18	19.9320940000	-4.6902290000	51.8115650000
C19	17.5408820000	-3.2342440000	51.9395220000
O20	17.2655080000	-4.4664940000	51.6038420000
O21	17.3449570000	-2.2355020000	51.2515630000
O22	18.2725720000	-4.3990430000	53.9009930000
C23	12.9067550000	-6.5566160000	54.6129970000
S24	13.6185540000	-3.7645930000	56.8375420000
S25	16.3627620000	-6.4517890000	55.5775550000
S26	9.6497880000	-5.1125560000	54.8552520000
S27	14.1063670000	-9.6391750000	55.3933130000
S28	14.7305070000	-4.0018850000	53.1729350000
S29	10.4322140000	-9.0514540000	54.2546610000
S30	15.2031430000	-7.3921470000	52.1544790000
S31	11.3965210000	-6.1875340000	51.3183370000
H32	13.6351750000	-2.1902110000	49.0425560000
C33	13.2979240000	-2.0241170000	50.0550230000
H34	13.9531390000	-2.5964560000	50.7176650000
H35	13.3700460000	-0.9589020000	50.3031500000
N36	11.9126650000	-2.5023120000	50.1485660000
H37	11.3313970000	-2.3939770000	49.3302480000
C38	11.2726340000	-2.9410160000	51.2415990000
N39	11.8294090000	-2.9404350000	52.4412680000
H40	11.3851660000	-3.5156190000	53.1568530000
H41	12.8469600000	-2.8874900000	52.5541260000
N42	9.9496030000	-3.2860420000	51.1125440000
H43	9.8070840000	-3.9083040000	50.3203530000
H44	9.6105120000	-3.7774640000	51.9399470000

H45	7.9297160000	0.3904920000	59.4128690000
C46	8.3442240000	-1.3522370000	60.6898680000
H47	8.2565220000	-1.5610060000	61.7631220000
H48	7.5366520000	-1.9050110000	60.1931560000
C49	9.6830200000	-1.8617480000	60.2512770000
N50	10.7215700000	-1.9820650000	61.1553530000
C51	10.1046690000	-2.2458170000	58.9968120000
H52	9.6028990000	-2.3163620000	58.0445670000
C53	11.7435230000	-2.4371380000	60.4528090000
H54	12.7282300000	-2.6641800000	60.8384480000
N55	11.4220380000	-2.6082080000	59.1426970000
H56	19.2898710000	-4.3166860000	57.5074330000
H57	16.7328160000	-8.8391600000	50.8019990000
C58	18.3998600000	-7.4179740000	50.5929440000
H59	17.7770760000	-6.5204990000	50.5628460000
H60	19.2033440000	-7.2668930000	49.8580590000
C61	19.0207270000	-7.5264660000	51.9503400000
N62	18.3885610000	-7.0640270000	53.0943780000
C63	20.2341750000	-8.0594220000	52.3132260000
H64	21.0196860000	-8.5148030000	51.7296120000
C65	19.1940890000	-7.3064980000	54.1171340000
H66	19.0219740000	-7.0351140000	55.1473970000
N67	20.3194210000	-7.9183200000	53.6873790000
H68	21.1000570000	-8.1504550000	54.2827170000
H69	6.8577750000	-10.6429210000	56.9567770000
C70	6.1750560000	-9.8249620000	56.6900010000
H71	5.5309550000	-10.2322280000	56.0424860000
H72	5.6932320000	-9.5371410000	57.6127210000
S73	8.5352400000	-8.5714430000	57.1686850000
H74	12.0194330000	-2.9772050000	58.4037670000
H75	18.4748370000	-4.2143550000	54.9027550000
C76	18.4780410000	-2.1976640000	55.7047240000
O77	18.8100030000	-3.3903050000	56.0844620000
O78	18.5976310000	-1.1671820000	56.3883360000
C79	21.7657450000	-3.6846360000	51.5430170000
O80	22.4525100000	-3.2716070000	50.6294570000
O81	22.3208170000	-4.2658020000	52.6398600000
H82	15.7024660000	-0.1996320000	61.4912310000
C83	14.8549940000	-0.0829910000	60.8320020000
H84	13.9165020000	-0.2867340000	61.3260940000
H85	14.8284760000	0.9922930000	60.5871390000
C86	15.0599850000	-0.8751950000	59.5441990000
H87	14.3688890000	-0.5149990000	58.7721800000
H88	14.8099730000	-1.9292860000	59.7138830000
C89	16.4928920000	-0.8155560000	59.0122620000
H90	16.9052650000	0.2005300000	59.0995630000
H91	16.5278940000	-1.0501710000	57.9421410000
C92	17.4391270000	-1.7846230000	59.7316180000
O93	17.0995030000	-2.4337800000	60.7264360000
N94	18.6875420000	-1.8523670000	59.1925050000

H95	19.2255010000	-2.6603230000	59.4844370000
H96	18.8036310000	-1.5726510000	58.2076890000
C97	10.6519930000	0.0100030000	53.3460010000
H98	11.1636990000	0.9592720000	53.2872700000
H99	9.5863320000	0.1850140000	53.3579380000
H100	10.9255170000	-0.5938880000	52.4812390000
C101	11.0777150000	-0.6222080000	54.6886790000
H102	10.7645160000	-1.6745880000	54.7097600000
C103	10.3709690000	0.0946650000	55.8501140000
H104	9.2795930000	0.0041500000	55.7706060000
H105	10.6170770000	1.1662930000	55.8558630000
H106	10.6719950000	-0.3189100000	56.8189350000
C107	12.6008070000	-0.5576590000	54.8734950000
H108	13.1393780000	-1.0486020000	54.0564490000
H109	12.9136750000	-1.0424940000	55.8039640000
H110	12.9371410000	0.4885860000	54.9103530000
H111	13.4550780000	-12.2303880000	53.1883140000
C112	13.9110010000	-11.6210560000	52.4219860000
H113	14.8254690000	-11.1637770000	52.8097800000
H114	14.0946350000	-12.1870220000	51.5047150000
N115	12.8453320000	-10.6337740000	52.2071830000
H116	12.2470450000	-10.4281510000	53.0132640000
C117	12.6358860000	-9.8544760000	51.1542810000
N118	13.6269280000	-9.6655860000	50.2341510000
H119	13.3718340000	-8.9886670000	49.5221160000
H120	14.5117790000	-9.4012700000	50.6727390000
N121	11.4230250000	-9.3078200000	50.9589190000
H122	10.7779810000	-9.4680540000	51.7382250000
H123	11.4246260000	-8.2882680000	50.7369540000
H124	19.1910080000	-8.2136540000	61.7152690000
C125	19.5978940000	-7.2298780000	61.9900510000
H126	20.6418980000	-7.3723410000	62.2885390000
C127	18.8476350000	-6.6670760000	63.1961090000
O128	19.4100280000	-6.1483600000	64.1566550000
C129	19.4918700000	-6.2717990000	60.7990630000
H130	19.8789340000	-5.2882620000	61.0896040000
H131	18.4378120000	-6.1118970000	60.5508380000
C132	20.2182480000	-6.7649150000	59.5367380000
H133	19.9391320000	-7.8020640000	59.3137240000
H134	21.3057050000	-6.7821680000	59.7021380000
C135	19.9895810000	-5.9970160000	58.2258610000
O136	20.2928410000	-6.4888550000	57.1506190000
O137	19.4718380000	-4.7804390000	58.3847090000
N138	17.4929040000	-6.7662870000	63.0688460000
H139	17.1361860000	-7.1321100000	62.1952140000
C140	16.5368850000	-6.0058850000	63.8530400000
H141	15.8946650000	-6.6966380000	64.4184100000
H142	17.1069990000	-5.4149040000	64.5742730000
C143	15.6559670000	-5.0979630000	62.9540330000
H144	16.2422210000	-4.2389710000	62.6108250000

H145	14.8323240000	-4.7103830000	63.5690130000
C146	15.1295480000	-5.8512130000	61.7445970000
C147	14.2054100000	-6.8999640000	61.8770450000
H148	13.7678680000	-7.1115330000	62.8517350000
C149	15.6630900000	-5.5866370000	60.4723560000
H150	16.3529610000	-4.7539280000	60.3530740000
C151	13.8544970000	-7.6873850000	60.7777880000
H152	13.1443810000	-8.5020450000	60.9021530000
C153	15.3200480000	-6.3795280000	59.3715530000
H154	15.7618350000	-6.1808570000	58.3980350000
C155	14.4258940000	-7.4436030000	59.5243300000
H156	14.1899760000	-8.0756600000	58.6719720000
H157	9.0037560000	0.7050730000	60.7752690000
C158	8.1269800000	0.1560800000	60.4649990000
H159	7.2836720000	0.4592420000	61.0677700000
H160	18.2592790000	-9.4987520000	50.1505400000
C161	17.5839740000	-8.6559210000	50.1520280000
H162	17.2773630000	-8.4419890000	49.1387990000
H163	23.2847110000	-4.2279500000	52.4968880000
H164	12.1210210000	-7.4650850000	57.9646090000
H165	13.8139350000	-4.6471480000	57.8466860000
C166	6.9968910000	-8.6563960000	56.1584740000
H167	7.3121230000	-8.7906860000	55.1197830000
H168	6.4875810000	-7.6959090000	56.2588810000
N169	11.4990670000	-7.9680170000	57.3336070000
H170	7.1493940000	-5.8354100000	59.8218940000
C171	6.9590150000	-4.8009330000	59.5000030000
H172	5.9039260000	-4.4903550000	59.5181450000
H173	7.4825770000	-4.1643590000	60.2246160000
C174	7.5772690000	-4.5372570000	58.1330330000
H175	8.6485880000	-4.7793850000	58.1656140000
H176	7.4906450000	-3.4650670000	57.8951490000
O177	6.9289630000	-5.3158210000	57.1252380000
H178	7.4781370000	-5.2338540000	56.3203050000
H179	9.3289380000	-9.1075730000	56.1729550000
H180	11.2650160000	-8.8521400000	57.7871700000
H181	11.0509670000	-5.3618830000	57.3750800000

The E_8 product structure after the TS in **Figure 11**
Energies: E= -7975.694040 solv = -0.197142, disp = --238.82 Z_0 = 899.88

Mo1	16.4707376162	-5.7971899063	53.2754307602
Fe2	9.7301739161	-6.9026320894	56.0917753954
Fe3	11.5783043294	-5.1185993095	55.6091795008
Fe4	10.9023864870	-6.6025035359	53.3224983559
Fe5	12.0498185896	-8.1929782710	55.3560247360
Fe6	14.4453014891	-7.7048906099	54.3637102350
Fe7	14.2447413666	-5.2248672374	55.0573517810
Fe8	13.2716683679	-5.9745938416	52.4776751211

C9	17.7972561184	-2.1852147263	54.2114725884
H10	17.9731202865	-1.1808541927	53.8210316416
H11	16.7134857852	-2.3014466642	54.3384652200
C12	18.2891653463	-3.2600343736	53.2162113991
C13	19.7589088963	-3.0022725619	52.8136574838
H14	20.3873036633	-3.1639676937	53.6941621803
H15	19.8491672477	-1.9516299202	52.5164429745
C16	20.2279591301	-3.8976440835	51.6475252109
H17	19.7737423884	-3.5847336680	50.7061800962
H18	19.9230378161	-4.9306862901	51.8519864729
C19	17.4978776113	-3.3991983141	51.8813992513
O20	17.1715577678	-4.6303498425	51.5903676870
O21	17.3531024837	-2.4200231406	51.1527965217
O22	18.1833090730	-4.5314801213	53.8827800502
C23	12.7053293137	-6.5324225815	54.4544599539
S24	13.3403361830	-3.8877016745	56.8361180606
S25	16.1252011347	-6.5561442291	55.5808395592
S26	9.5401619534	-4.8829704890	54.4781192625
S27	13.7475466531	-9.6941233699	55.2641306128
S28	14.6907023754	-4.0356738838	53.1734818497
S29	9.9801035151	-8.6509498997	54.2575843177
S30	15.1031628149	-7.4757324782	52.1286339340
S31	11.4341611438	-6.1625288118	51.0441595310
H32	13.6351745263#	-2.1902109839#	49.0425561678#
C33	13.2979237535#	-2.0241170250#	50.0550234193#
H34	13.9567029341	-2.5938367334	50.7163285938
H35	13.3651854890	-0.9580494403	50.3010523962
N36	11.9145648796	-2.5070622456	50.1528151426
H37	11.3442181683	-2.4623287022	49.3209304909
C38	11.2717111500	-2.9358577539	51.2479129441
N39	11.8173905097	-2.9064001972	52.4525356075
H40	11.3492507746	-3.4546834464	53.1789955820
H41	12.8311436570	-2.8425089254	52.5731040133
N42	9.9557953345	-3.3103951945	51.1092937016
H43	9.8752847629	-4.0387098772	50.3980026117
H44	9.5932570819	-3.7124668085	51.9789893911
H45	7.9319451177	0.3851065401	59.4119577065
C46	8.3351003736	-1.3521871016	60.7011527457
H47	8.2357125336	-1.5560467082	61.7741412922
H48	7.5310850820	-1.9050226840	60.1989562729
C49	9.6779892042	-1.8633052015	60.2817163122
N50	10.6923407366	-2.0216984759	61.2076604273
C51	10.1313322663	-2.1989552301	59.0258344324
H52	9.6554749117	-2.2296109354	58.0584067613
C53	11.7296267033	-2.4576661173	60.5156915364
H54	12.7016747509	-2.7073580993	60.9184669743
N55	11.4435978029	-2.5713481125	59.1921141340
H56	19.2500523720	-4.3683522925	57.4755827337
H57	16.7250376256	-8.8503977789	50.7898532364
C58	18.3936458331	-7.4301674908	50.6252066591

H59	17.7815766127	-6.5261687635	50.5845093957
H60	19.2258630379	-7.2784238334	49.9233751715
C61	18.9613628661	-7.5671908419	52.0038165777
N62	18.2571517088	-7.1934038339	53.1390203966
C63	20.1853756971	-8.0522394987	52.3964126023
H64	21.0182730591	-8.4365622353	51.8280454576
C65	19.0294651700	-7.4472410544	54.1838362324
H66	18.7996997320	-7.2296440842	55.2152595723
N67	20.2032153797	-7.9789560609	53.7791952942
H68	20.9757700801	-8.1774609207	54.3973935665
H69	6.9281830658	-10.5871866927	56.9278628862
C70	6.1750562591#	-9.8249624695#	56.6900010212#
H71	5.5309553412#	-10.2322279166#	56.0424859937#
H72	5.6932323907#	-9.5371414460#	57.6127204981#
S73	7.9846664042	-7.9373108184	57.4838286157
H74	12.0529668506	-2.9159830799	58.4506587093
H75	18.3908946855	-4.3214644144	54.8783531959
C76	18.4232811999	-2.2885105340	55.6337651766
O77	18.7383107478	-3.4781730093	56.0401370255
O78	18.5388402809	-1.2456893440	56.3004706175
C79	21.7162158469	-3.8935551667	51.4808695560
O80	22.3626488971	-3.6238045450	50.4873120471
O81	22.3208166961#	-4.2658019549#	52.6398596201#
H82	15.7024655401#	-0.1996319673#	61.4912314204#
C83	14.8549940396#	-0.0829906917#	60.8320019163#
H84	13.9165015892#	-0.2867338796#	61.3260937710#
H85	14.8270931870	0.9916935223	60.5845249630
C86	15.0599851885#	-0.8751946593#	59.5441989628#
H87	14.3603315837	-0.5225593797	58.7762562639
H88	14.8258504762	-1.9324396662	59.7137151724
C89	16.4909760146	-0.7924745297	59.0055586654
H90	16.8824717070	0.2321756990	59.0943610443
H91	16.5269893644	-1.0235891620	57.9349908487
C92	17.4595711511	-1.7412379016	59.7230898017
O93	17.1706748260	-2.3253265056	60.7738783968
N94	18.6699992939	-1.8661583496	59.1153508502
H95	19.2212199496	-2.6575665659	59.4266583887
H96	18.7505550069	-1.6222277558	58.1172291134
C97	10.6519929146#	0.0100026536#	53.3460010658#
H98	11.1636988006#	0.9592721635#	53.2872699055#
H99	9.5863320895#	0.1850135763#	53.3579375146#
H100	10.9258143617	-0.5945463904	52.4833294021
C101	11.0667647592	-0.6210723384	54.6918468485
H102	10.7507865980	-1.6721734849	54.7101279945
C103	10.3482963125	0.0976074084	55.8448347280
H104	9.2585882689	-0.0062581343	55.7608969334
H105	10.5824558230	1.1719773850	55.8435161674
H106	10.6492942348	-0.3048697546	56.8181918548
C107	12.5869599755	-0.5612958001	54.8941907267
H108	13.1331055822	-1.0488176753	54.0800068703

H109	12.8865435329	-1.0538330715	55.8251697583
H110	12.9262941303	0.4835659803	54.9418548135
H111	13.4550784025#	-12.2303882132#	53.1883144604#
C112	13.9110008158#	-11.6210557649#	52.4219862188#
H113	14.8486781383	-11.1885722341	52.7800989424
H114	14.0469044946	-12.1754710581	51.4903188743
N115	12.8549837087	-10.6169341091	52.2648323869
H116	12.4212516948	-10.3404074685	53.1488777530
C117	12.5598679782	-9.8611031359	51.2148278799
N118	13.4556451319	-9.7286273936	50.1900819094
H119	13.1510483618	-9.0566339742	49.4923823879
H120	14.3903193833	-9.4960163594	50.5262393529
N121	11.3523208136	-9.2885261813	51.1214220251
H122	10.7509506095	-9.4031180788	51.9430132745
H123	11.3437446187	-8.2878783529	50.8150535606
H124	19.1968506004	-8.2149280018	61.7114496798
C125	19.5978943810#	-7.2298783766#	61.9900511014#
H126	20.6406838046	-7.3685476657	62.2943275407
C127	18.8379099751	-6.6777404025	63.1950942871
O128	19.3925345794	-6.1819331933	64.1728105261
C129	19.4918705178#	-6.2717993225#	60.7990627399#
H130	19.8782064424	-5.2868068056	61.0857420154
H131	18.4381630996	-6.1139587713	60.5492791176
C132	20.2200367432	-6.7718983095	59.5372765030
H133	19.9526860427	-7.8152594360	59.3288902441
H134	21.3078417360	-6.7752899350	59.7003129921
C135	19.9822404239	-6.0259708676	58.2143788046
O136	20.2975622565	-6.5244983755	57.1447550831
O137	19.4428876708	-4.8169570059	58.3584735066
N138	17.4826496441	-6.7647765028	63.0542335442
H139	17.1268090670	-7.0981165266	62.1668576476
C140	16.5368848888#	-6.0058851248#	63.8530402489#
H141	15.8694111174	-6.6999101437	64.3838962574
H142	17.1150999194	-5.4620451364	64.6042288933
C143	15.6888115137	-5.0364324364	62.9842202607
H144	16.3077829901	-4.1912594041	62.6647851678
H145	14.8869486255	-4.6322553945	63.6167977890
C146	15.1073668236	-5.7191651090	61.7567465704
C147	14.0693616929	-6.6604338352	61.8601719474
H148	13.6034651028	-6.8420742934	62.8275938550
C149	15.6704304656	-5.4825766417	60.4909254434
H150	16.4349236442	-4.7163400258	60.3880540913
C151	13.6292266823	-7.3672663947	60.7377328720
H152	12.8247830079	-8.0922375026	60.8377114976
C153	15.2423425931	-6.2004411256	59.3681066650
H154	15.7022999166	-6.0242479840	58.3994524944
C155	14.2264290509	-7.1537753589	59.4896014990
H156	13.9126815305	-7.7245894055	58.6194537533
H157	9.0037560457#	0.7050733645#	60.7752692845#
C158	8.1269802833#	0.1560797007#	60.4649992347#

H159	7.2836721311#	0.4592420839#	61.0677701464#
H160	18.2592788545#	-9.4987523137#	50.1505398710#
C161	17.5839741936#	-8.6559209134#	50.1520279756#
H162	17.2773629374#	-8.4419888806#	49.1387987422#
H163	23.2784515530	-4.2344371623	52.4573347245
H164	11.7219268978	-7.4080870847	57.8531521625
H165	13.4595452158	-4.8336824013	57.7968747166
C166	6.8524217285	-8.5672679504	56.1714402846
H167	7.4413328177	-8.7233731371	55.2646656694
H168	6.1424161395	-7.7522777965	56.0047367655
N169	11.2431882232	-8.0039198359	57.1806860184
H170	7.1493940387#	-5.8354098625#	59.8218944181#
C171	6.9590151759#	-4.8009333262#	59.5000033748#
H172	5.9039259860#	-4.4903550437#	59.5181449229#
H173	7.4766969458	-4.1691657065	60.2337795245
C174	7.5804158750	-4.4970051049	58.1401009262
H175	8.6443679651	-4.7634096582	58.1480197584
H176	7.5163312150	-3.4134931535	57.9541299440
O177	6.9057588766	-5.2007166731	57.0903764052
H178	7.4501138904	-5.0851762834	56.2853744095
H179	8.6811593849	-9.0913980971	57.5933598308
H180	11.0840151736	-8.9143372645	57.6154415350
H181	10.5797403528	-5.5376870636	57.0133490595

The E_8 TS structure in **Figure 12**
Energies: E= -7975.689313 solv = -0.193907, disp = --235.95 Z_0 = 897.61

Mo1	16.4270270000	-5.7300360000	53.3144570000
Fe2	9.3972250000	-6.8106230000	55.5854290000
Fe3	11.4470160000	-5.1754200000	55.5111780000
Fe4	10.8902290000	-6.5045270000	53.0936240000
Fe5	11.8427660000	-8.1489370000	55.1789570000
Fe6	14.3335290000	-7.6440910000	54.3051790000
Fe7	14.1464780000	-5.2048990000	55.0738180000
Fe8	13.3043900000	-5.8532170000	52.4262230000
C9	17.8124020000	-2.1152450000	54.3524220000
H10	18.0005740000	-1.1149340000	53.9579010000
H11	16.7285250000	-2.2136150000	54.4948370000
C12	18.2683680000	-3.1981410000	53.3527320000
C13	19.7466070000	-2.9848780000	52.9495790000
H14	20.3735640000	-3.1922810000	53.8218300000
H15	19.8746870000	-1.9308280000	52.6788760000
C16	20.1785370000	-3.8650340000	51.7576430000
H17	19.6906170000	-3.5409040000	50.8372870000
H18	19.8867350000	-4.9017400000	51.9593120000
C19	17.4722930000	-3.2940500000	52.0210770000
O20	17.1630980000	-4.5186220000	51.6838590000
O21	17.3113460000	-2.2911940000	51.3317390000
O22	18.1194060000	-4.4707840000	54.0075270000

C23	12.6166910000	-6.4900820000	54.3504300000
S24	13.1003650000	-4.0361270000	56.9179680000
S25	16.0137430000	-6.5601700000	55.5898980000
S26	9.4934790000	-4.7586580000	54.2376150000
S27	13.5622050000	-9.6427240000	55.1285290000
S28	14.6516920000	-3.9391110000	53.2524200000
S29	9.8413580000	-8.5379230000	53.9019260000
S30	15.0981160000	-7.3934600000	52.1008100000
S31	11.5585310000	-6.0412970000	50.8817300000
H32	13.6351750000	-2.1902110000	49.0425560000
C33	13.2979240000	-2.0241170000	50.0550230000
H34	13.9034440000	-2.6510730000	50.7149140000
H35	13.4309890000	-0.9711360000	50.3276640000
N36	11.8854040000	-2.4157160000	50.0993250000
H37	11.3587570000	-2.3666900000	49.2400500000
C38	11.2037570000	-2.8669970000	51.1588450000
N39	11.7188280000	-2.8740490000	52.3764100000
H40	11.2187750000	-3.4193700000	53.0841230000
H41	12.7265360000	-2.8055850000	52.5284480000
N42	9.8860700000	-3.2088580000	50.9788250000
H43	9.7872980000	-3.8700460000	50.2108110000
H44	9.4970640000	-3.6487660000	51.8175440000
H45	7.9320570000	0.3895060000	59.4126140000
C46	8.3465400000	-1.3559060000	60.6935470000
H47	8.1352690000	-1.5827950000	61.7456480000
H48	7.6203640000	-1.9196810000	60.0963120000
C49	9.7409230000	-1.8290080000	60.4162130000
N50	10.6845370000	-1.8545390000	61.4269230000
C51	10.3074520000	-2.2493730000	59.2326000000
H52	9.9099340000	-2.3947030000	58.2399160000
C53	11.7941980000	-2.2865750000	60.8575540000
H54	12.7406670000	-2.4500380000	61.3540010000
N55	11.6205070000	-2.5332630000	59.5313580000
H56	19.2867940000	-4.2954800000	57.5422450000
H57	16.7190630000	-8.8631540000	50.7802460000
C58	18.3634950000	-7.4238640000	50.6598430000
H59	17.7346680000	-6.5311040000	50.6182150000
H60	19.2066600000	-7.2469690000	49.9774950000
C61	18.9036140000	-7.5649840000	52.0511980000
N62	18.2137700000	-7.1199430000	53.1702770000
C63	20.0965310000	-8.1051750000	52.4690740000
H64	20.9121800000	-8.5493680000	51.9191280000
C65	18.9667460000	-7.3793350000	54.2279180000
H66	18.7429260000	-7.1214480000	55.2511750000
N67	20.1121570000	-7.9857160000	53.8478400000
H68	20.8653070000	-8.2140920000	54.4794500000
H69	6.9299130000	-10.5809680000	56.9414580000
C70	6.1750560000	-9.8249620000	56.6900010000
H71	5.5309550000	-10.2322280000	56.0424860000
H72	5.6932320000	-9.5371410000	57.6127210000

S73	7.9372980000	-7.7635500000	57.3217480000
H74	12.3042550000	-2.8905150000	58.8682840000
H75	18.3252320000	-4.2795540000	55.0042580000
C76	18.4669350000	-2.2363730000	55.7576520000
O77	18.7184460000	-3.4393030000	56.1677840000
O78	18.6664340000	-1.1987660000	56.4104190000
C79	21.6593930000	-3.8360880000	51.5333350000
O80	22.2577180000	-3.4963640000	50.5317490000
O81	22.3208170000	-4.2658020000	52.6398600000
H82	15.7024660000	-0.1996320000	61.4912310000
C83	14.8549940000	-0.0829910000	60.8320020000
H84	13.9165020000	-0.2867340000	61.3260940000
H85	14.8279280000	0.9920980000	60.5867000000
C86	15.0599850000	-0.8751950000	59.5441990000
H87	14.3698410000	-0.5122120000	58.7723060000
H88	14.8119710000	-1.9296690000	59.7130800000
C89	16.4934930000	-0.8153810000	59.0117940000
H90	16.8924460000	0.2082220000	59.0659830000
H91	16.5312080000	-1.0828270000	57.9493340000
C92	17.4520640000	-1.7503710000	59.7607790000
O93	17.1197130000	-2.3752590000	60.7730930000
N94	18.7028770000	-1.8143610000	59.2275610000
H95	19.2569140000	-2.5994890000	59.5494090000
H96	18.8257410000	-1.5574320000	58.2374410000
C97	10.6519930000	0.0100030000	53.3460010000
H98	11.1636990000	0.9592720000	53.2872700000
H99	9.5863320000	0.1850140000	53.3579380000
H100	10.9253910000	-0.5909580000	52.4810180000
C101	11.0653600000	-0.6325550000	54.6881490000
H102	10.7433210000	-1.6817630000	54.6966270000
C103	10.3529130000	0.0747100000	55.8521980000
H104	9.2621310000	-0.0109720000	55.7633400000
H105	10.6036870000	1.1451300000	55.8736980000
H106	10.6451420000	-0.3526100000	56.8185490000
C107	12.5868210000	-0.5815790000	54.8902810000
H108	13.1331210000	-1.0560690000	54.0685650000
H109	12.8840670000	-1.0910730000	55.8136380000
H110	12.9302220000	0.4608970000	54.9566550000
H111	13.4550780000	-12.2303880000	53.1883140000
C112	13.9110010000	-11.6210560000	52.4219860000
H113	14.8135650000	-11.1407310000	52.8100200000
H114	14.1199420000	-12.2075020000	51.5226930000
N115	12.8355140000	-10.6605310000	52.1593020000
H116	12.3599600000	-10.3564950000	53.0128030000
C117	12.6740240000	-9.8609090000	51.1083760000
N118	13.6444760000	-9.7780960000	50.1502930000
H119	13.4520820000	-9.0598810000	49.4591450000
H120	14.5754610000	-9.6534370000	50.5426600000
N121	11.5184680000	-9.2064070000	50.9393920000
H122	10.8418550000	-9.2953650000	51.7025750000

H123	11.5772890000	-8.2090670000	50.6357810000
H124	19.2118760000	-8.2195790000	61.7058160000
C125	19.5978940000	-7.2298780000	61.9900510000
H126	20.6402820000	-7.3573100000	62.3003280000
C127	18.8291690000	-6.6971400000	63.1955910000
O128	19.3797340000	-6.2527510000	64.1996810000
C129	19.4918710000	-6.2717990000	60.7990630000
H130	19.8370230000	-5.2757050000	61.0986840000
H131	18.4395390000	-6.1454410000	60.5237390000
C132	20.2643900000	-6.7356960000	59.5527440000
H133	20.0599150000	-7.7915400000	59.3396680000
H134	21.3482240000	-6.6697150000	59.7300520000
C135	19.9796580000	-5.9914370000	58.2410060000
O136	20.1915490000	-6.5135660000	57.1574100000
O137	19.5202610000	-4.7549790000	58.4130980000
N138	17.4764540000	-6.7382060000	63.0268290000
H139	17.1221400000	-7.0425010000	62.1287830000
C140	16.5368850000	-6.0058850000	63.8530400000
H141	15.8824070000	-6.7104040000	64.3868280000
H142	17.1228280000	-5.4647720000	64.6003730000
C143	15.6730260000	-5.0341560000	63.0106260000
H144	16.2937060000	-4.2025270000	62.6596880000
H145	14.9009670000	-4.6135810000	63.6691930000
C146	15.0388200000	-5.7254710000	61.8170230000
C147	13.9946170000	-6.6504300000	61.9777150000
H148	13.5729780000	-6.8172340000	62.9679140000
C149	15.5522960000	-5.5150950000	60.5272370000
H150	16.3311430000	-4.7696970000	60.3836090000
C151	13.4894070000	-7.3573390000	60.8848520000
H152	12.6766090000	-8.0656550000	61.0285630000
C153	15.0598650000	-6.2345170000	59.4327100000
H154	15.4791570000	-6.0736130000	58.4430310000
C155	14.0293640000	-7.1621860000	59.6090720000
H156	13.6541140000	-7.7231200000	58.7565350000
H157	9.0037560000	0.7050730000	60.7752690000
C158	8.1269800000	0.1560800000	60.4649990000
H159	7.2836720000	0.4592420000	61.0677700000
H160	18.2592790000	-9.4987520000	50.1505400000
C161	17.5839740000	-8.6559210000	50.1520280000
H162	17.2773630000	-8.4419890000	49.1387990000
H163	23.2684770000	-4.2055990000	52.4171020000
H164	11.2873000000	-7.6806940000	57.7116370000
H165	13.1506860000	-5.0589160000	57.8018890000
C166	6.8439270000	-8.5988740000	56.0977270000
H167	7.4287260000	-8.8372040000	55.2060750000
H168	6.1054010000	-7.8352390000	55.8301860000
N169	10.9556320000	-8.3186360000	56.9872810000
H170	7.1493940000	-5.8354100000	59.8218940000
C171	6.9590150000	-4.8009330000	59.5000030000
H172	5.9039260000	-4.4903550000	59.5181450000

H173	7.4729520000	-4.1781480000	60.2451340000
C174	7.5444700000	-4.4485350000	58.1357620000
H175	8.5427920000	-4.8861350000	58.0206040000
H176	7.6554720000	-3.3537340000	58.0730490000
O177	6.6772450000	-4.8899600000	57.0875310000
H178	7.2043060000	-4.8818320000	56.2687250000
H179	9.1562260000	-8.4872040000	57.2500560000
H180	11.2169410000	-9.2660560000	57.2730100000
H181	10.3424830000	-5.7749160000	56.7572250000

The E_8 product structure after the TS in **Figure 12**

Energies: E= -7975.742790 solv = -0.195816, disp = --239.98 Z_0 = 901.52

Mo1	16.3137018908	-5.7675737912	53.2147636910
Fe2	8.9286822560	-6.5463530951	55.4018640956
Fe3	11.2900471520	-5.2107213459	55.3020763034
Fe4	10.7156800074	-6.4645477459	52.8164887922
Fe5	11.6527041589	-7.9666919124	55.0144400455
Fe6	14.1443993941	-7.6326183820	54.0947299026
Fe7	13.9829754061	-5.2043477053	54.8791065178
Fe8	13.1877814288	-5.7907825503	52.1827114702
C9	17.7742613019	-2.1925412468	54.2051379305
H10	17.9979884322	-1.2003986419	53.8086896282
H11	16.6848097073	-2.2614411941	54.3192593395
C12	18.2312760229	-3.2961232825	53.2264232991
C13	19.7226742217	-3.1187262090	52.8546405260
H14	20.3262588820	-3.3053399090	53.7475506772
H15	19.8689339779	-2.0743265558	52.5569962750
C16	20.1747574459	-4.0374970130	51.7009414516
H17	19.6923427620	-3.7580769328	50.7631164577
H18	19.8913415410	-5.0700136157	51.9369370074
C19	17.4635074725	-3.3929391576	51.8762707281
O20	17.0977215377	-4.6090687328	51.5679343685
O21	17.3702461156	-2.4033794065	51.1545616625
O22	18.0363089886	-4.5564384050	53.8969315252
C23	12.4868425621	-6.4215217963	54.1061231798
S24	12.9322519685	-4.0380521023	56.7081947406
S25	15.7999989673	-6.5823218380	55.4728092527
S26	9.4028935929	-4.6670891833	53.9740278718
S27	13.3168119236	-9.5693035195	55.0100780612
S28	14.5852845996	-3.9372914415	53.0938673259
S29	9.6847067131	-8.3790810945	53.8226038226
S30	14.9773628581	-7.3693026329	51.9251771358
S31	11.4594323623	-6.0527074709	50.6358666989
H32	13.6351744779#	-2.1902108308#	49.0425561266#
C33	13.2979238817#	-2.0241171818#	50.0550234428#
H34	13.8647246378	-2.6880134501	50.7120385687
H35	13.4657415156	-0.9811236150	50.3451809065
N36	11.8708111748	-2.3616737662	50.0572104853

H37	11.3718352322	-2.2999296700	49.1820524165
C38	11.1518065957	-2.8240372972	51.0846236485
N39	11.6290330770	-2.8522081055	52.3159192051
H40	11.0960154678	-3.3851781567	53.0114100434
H41	12.6262613493	-2.7514119629	52.5043666518
N42	9.8444945728	-3.1684792268	50.8446880833
H43	9.7928922790	-3.8609053329	50.0976560250
H44	9.4044408380	-3.5750247644	51.6754073902
H45	7.9321719224	0.3877549647	59.4124528137
C46	8.3372012739	-1.3545700216	60.6948171781
H47	8.2049208042	-1.5694990177	61.7620509289
H48	7.5571898236	-1.9110788930	60.1617060044
C49	9.6973415872	-1.8491167746	60.3125270259
N50	10.6989090424	-1.9578002449	61.2604536050
C51	10.1804519959	-2.2055578482	59.0741914126
H52	9.7192344709	-2.2796020022	58.1017424163
C53	11.7611808898	-2.3765950189	60.5985733103
H54	12.7320746202	-2.5893308879	61.0245398451
N55	11.5018396434	-2.5338865314	59.2731857697
H56	19.1845023538	-4.3807572306	57.4747665798
H57	16.7177399572	-8.8603046652	50.7784889918
C58	18.3751350046	-7.4330448109	50.6593086521
H59	17.7705413741	-6.5260274157	50.5822205142
H60	19.2467478852	-7.2890562653	50.0059643279
C61	18.8553664803	-7.5716367953	52.0711230689
N62	18.0705573177	-7.2120287658	53.1578239815
C63	20.0555254421	-8.0451514942	52.5440355119
H64	20.9287654593	-8.4179179928	52.0312943995
C65	18.7758272984	-7.4613885546	54.2502540783
H66	18.4746452872	-7.2505197722	55.2638587487
N67	19.9799630149	-7.9766964705	53.9253285178
H68	20.7139119336	-8.1489795695	54.5970438070
H69	6.9468816926	-10.5637777777	56.9373603413
C70	6.1750559329#	-9.8249625926#	56.6900011828#
H71	5.5309553907#	-10.2322279091#	56.0424859396#
H72	5.6932323434#	-9.5371414510#	57.6127204751#
S73	8.0092728725	-7.8147584893	57.1634226491
H74	12.1341667393	-2.8621943712	58.5474495794
H75	18.2334747844	-4.3543162349	54.8943310810
C76	18.3838853857	-2.3134525447	55.6317622238
O77	18.6106760968	-3.5160692610	56.0592840901
O78	18.5727739522	-1.2705597901	56.2813827236
C79	21.6576491347	-4.0188402346	51.4796409224
O80	22.2557878195	-3.8370899170	50.4371665317
O81	22.3208167502#	-4.2658019498#	52.6398596166#
H82	15.7024655435#	-0.1996319612#	61.4912314171#
C83	14.8549940075#	-0.0829907185#	60.8320019277#
H84	13.9165015893#	-0.2867338795#	61.3260937710#
H85	14.8250278434	0.9910467120	60.5826806893
C86	15.0599852146#	-0.8751946303#	59.5441989570#

H87	14.3501652333	-0.5319282152	58.7811761245
H88	14.8431342739	-1.9359509198	59.7173009601
C89	16.4877389316	-0.7670867197	58.9949512121
H90	16.8532677669	0.2680550369	59.0714308225
H91	16.5236821499	-1.0075441761	57.9263487559
C92	17.4831509851	-1.6830455866	59.7198528062
O93	17.2204471825	-2.2385224397	60.7934788617
N94	18.6823273457	-1.8168048772	59.0948161844
H95	19.2555073576	-2.5811807299	59.4321172354
H96	18.7546942149	-1.6000529652	58.0892024497
C97	10.6519929043#	0.0100027017#	53.3460011269#
H98	11.1636988102#	0.9592721552#	53.2872698554#
H99	9.5863320840#	0.1850135418#	53.3579375205#
H100	10.9221962369	-0.5864464845	52.4773648120
C101	11.0732525386	-0.6343976408	54.6821502227
H102	10.7447197024	-1.6819066009	54.6950229351
C103	10.3759516164	0.0815029670	55.8492458533
H104	9.2846203514	-0.0050746646	55.7721010772
H105	10.6278208272	1.1518143942	55.8587331946
H106	10.6778459941	-0.3376224699	56.8153203459
C107	12.5966273730	-0.5928006210	54.8666723615
H108	13.1311742874	-1.0820702486	54.0452443211
H109	12.9010313087	-1.0927325376	55.7925242568
H110	12.9486726320	0.4475831900	54.9154468167
H111	13.4550783285#	-12.2303881632#	53.1883144562#
C112	13.9110009166#	-11.6210558003#	52.4219861865#
H113	14.8003120394	-11.1196260193	52.8143808930
H114	14.1419101612	-12.2134605809	51.5326076861
N115	12.8217781155	-10.6818664633	52.1384898372
H116	12.3966323851	-10.3307394413	53.0079355271
C117	12.6464557584	-9.9078257950	51.0727874147
N118	13.5921692397	-9.8527021673	50.0870340540
H119	13.3832908728	-9.1564001272	49.3783466442
H120	14.5331209797	-9.7188658730	50.4506425652
N121	11.4891769777	-9.2515881306	50.9134911928
H122	10.8178538325	-9.3234556608	51.6815823238
H123	11.5282691444	-8.2780131472	50.5493487755
H124	19.2010894924	-8.2163819146	61.7100379053
C125	19.5978942105#	-7.2298785647#	61.9900510010#
H126	20.6401428526	-7.3660460900	62.2966132591
C127	18.8342356982	-6.6854609149	63.1957920007
O128	19.3851997944	-6.2160585091	64.1881046373
C129	19.4918707024#	-6.2717991637#	60.7990628381#
H130	19.8827547925	-5.2881274250	61.0836626401
H131	18.4370134254	-6.1077076397	60.5558815562
C132	20.2073118600	-6.7706090527	59.5290827865
H133	19.9661551506	-7.8237768728	59.3402629398
H134	21.2981019556	-6.7387723497	59.6672651550
C135	19.9121092227	-6.0470924852	58.2050731083
O136	20.1526714596	-6.5754833913	57.1297972980

O137	19.4104421506	-4.8230616786	58.3540614605
N138	17.4792438901	-6.7514282224	63.0401283466
H139	17.1260760538	-7.0618806600	62.1438002180
C140	16.5368849531#	-6.0058849891#	63.8530402938#
H141	15.8411428956	-6.7045511902	64.3395139881
H142	17.1133213517	-5.5106946238	64.6381844662
C143	15.7352442319	-4.9771954944	63.0150430360
H144	16.3836029089	-4.1364206243	62.7466436662
H145	14.9314920116	-4.5798853516	63.6498277845
C146	15.1647478868	-5.5847975614	61.7448650049
C147	14.1267993406	-6.5309012753	61.7822728028
H148	13.6620568788	-6.7840686855	62.7336942286
C149	15.7315322212	-5.2602936412	60.5006246265
H150	16.5056156511	-4.4984042630	60.4553984641
C151	13.6828167453	-7.1522447637	60.6120086742
H152	12.8801141308	-7.8843434326	60.6618386005
C153	15.2939890531	-5.8852840085	59.3280261385
H154	15.7469980049	-5.6373670736	58.3720177570
C155	14.2746535786	-6.8406507544	59.3826604563
H156	13.9651384351	-7.3444646512	58.4706731685
H157	9.0037560385#	0.7050733743#	60.7752692874#
C158	8.1269803497#	0.1560797534#	60.4649991920#
H159	7.2836721317#	0.4592420427#	61.0677701680#
H161	18.2592788128#	-9.4987523472#	50.1505398838#
C162	17.5839744299#	-8.6559207585#	50.1520278604#
H163	17.2773628257#	-8.4419889684#	49.1387987575#
H164	23.2681796284	-4.2392399167	52.4088744855
H166	11.4949110426	-7.2936765834	57.7103594558
H167	13.0698366426	-5.0503450221	57.5944637848
C168	6.8021497694	-8.6154072459	56.0316127622
H169	7.3119365582	-8.9111070960	55.1100276505
H170	6.0231317020	-7.8890639318	55.7650222396
N171	11.3677676352	-8.1445828023	57.1646565605
H173	7.1493940293#	-5.8354098663#	59.8218944113#
C174	6.9590151295#	-4.8009334150#	59.5000033802#
H175	5.9039259930#	-4.4903550211#	59.5181449407#
H176	7.4917996859	-4.1692685563	60.2250718347
C177	7.4731143688	-4.4756811990	58.0964775248
H178	8.5124989392	-4.8052250412	57.9678205373
H179	7.4550136513	-3.3894800901	57.9486590526
O180	6.6422613931	-5.0181613914	57.0753260522
H181	6.8398652065	-5.9809795149	57.0897167347
H182	10.3999777832	-8.4514648533	57.3066249560
H183	12.0152576227	-8.8656024171	57.4804495491
H184	10.1785516028	-5.8287796749	56.5050060563
